# Mindfulness‐Based Programmes for Work Performance: A Systematic Review and Meta‐Analysis of Randomised Controlled Trials

**DOI:** 10.1002/smi.70123

**Published:** 2025-12-05

**Authors:** Maris Vainre, Tim Dalgleish, Tia Bendriss‐Otiko, Molly Butler, Amelia Kirkpatrick, Nana Kosugiyama, Fabiana Mariscotti, Candelaria Martinez‐Sosa, Athina Sideri, Sebastian Sönksen, Tim Wood, Caitlin Hitchcock, Julieta Galante

**Affiliations:** ^1^ Medical Research Council Cognition and Brain Sciences Unit University of Cambridge Cambridge UK; ^2^ Institute of Psychology University of Tartu Tartu Estonia; ^3^ School of Psychological Sciences University of Melbourne Victoria Australia; ^4^ Contemplative Studies Centre University of Melbourne Victoria Australia; ^5^ Cambridgeshire and Peterborough NHS Foundation Trust Cambridge UK; ^6^ North East London NHS Foundation Trust Rainham UK; ^7^ Department of Psychiatry University of Cambridge Cambridge UK; ^8^ Norfolk and Suffolk NHS Foundation Trust Norwich UK

**Keywords:** meta‐analysis, mindfulness, systematic review, work performance

## Abstract

Employers and universities globally subsidise access to mindfulness‐based programmes (MBPs) for their employees and students to improve work performance, despite unclear evidence. This paper offers the highest quality synthesis of MBPs' impact on work performance in academic and occupational settings to date (PROSPERO #191756). On 2^nd^ August 2024, we searched eight databases. The primary outcome was task performance—the quantity and quality of completed tasks assigned to the individual. Secondary outcomes were contextual performance, adaptive performance, and counter‐productive behaviour. Two independent reviewers selected studies, extracted data, and appraised risks of bias. We conducted pairwise random‐effects meta‐analyses of 99 studies (*N* = 16,054). MBPs were found to improve task performance at post‐intervention compared to passive control groups (*k* = 22, Hedges' *g* = 0.25, 95% CI 0.06–0.44, *p* = 0.01, *I* ^2^ = 81.48%) but not compared to active control groups (*k* = 4, Hedges' *g* = 0.12, 95% CI −0.3–0.55, *p* = 0.43, *I* ^2^ = 62.87%). MBPs improved adaptive performance and contextual performance. Effects may last several months. Confidence in the review results, per Grading of Recommendations Assessment, Development and Evaluation (GRADE), is very low.

## 1 Introduction

Enhancing work performance is a shared priority for both individuals and organisations. According to the World Health Organization's definition of health (World Health Organization 1948), working well is an indicator of well‐being. For individuals, strong performance can foster a sense of achievement, open career opportunities, and increase income. For organisations—such as employers, universities, and schools—the performance of each person contributes to achieving strategic goals, driving growth, and ultimately generating revenue. Both individuals and organisations can take steps to improve performance. At the individual level, evidence‐based strategies such as improving sleep quality, engaging in physical exercise, and maintaining a healthy diet have been shown to reduce psychological distress and enhance performance (Kent et al. 2015; Lieberman 2003; Llewellyn et al. 2008). Beyond these lifestyle factors, organisations are increasingly exploring structured interventions to support employee well‐being and performance.

Mindfulness‐based programs (MBPs) have emerged as a recommended approach for promoting well‐being in the workplace. In this paper, we follow the definition of MBPs proposed by Crane and her colleagues: complex interventions that aim to improve compassion (Crane et al. 2017), attention, and self‐regulation (Crane et al. 2017; Hölzel et al. 2011; Verdonk et al. 2020), through training the ability to maintain awareness of the present moment (Kabat‐Zinn 2003) and decentering from psychological stressors (Crane et al. 2017; Hölzel et al. 2011; Verdonk et al. 2020; Bennett et al. 2021). The National Institute for Health and Care Excellence (NICE) advises organisations to offer mindfulness‐based programmes (MBPs) (National Institute for Health and Clinical Excellence 2022) and many organisations are now doing so (Barnes et al. 2017; Chen 2022; Fleming 2021; The Prince's Responsible Business Network 2019).

It has been argued that MBPs directly improve performance (Baltzell 2016; Chapman‐Clarke 2016; Cheung et al. 2020; Dane 2011; Good et al. 2016; Hyland 2015; Reb et al. 2017), making them particularly attractive to employers compared to other well‐being interventions. However, despite their widespread adoption, evidence on the effectiveness of MBPs for improving work performance remains limited, and even less is known about their comparative effectiveness relative to alternative interventions (Micklitz et al. 2021; Lomas et al. 2017; Bartlett et al. 2019; Vonderlin et al. 2020; Michaelsen et al. 2023; Dawson et al. 2019).

Six prior systematic reviews (Micklitz et al. 2021; Lomas et al. 2017; Bartlett et al. 2019; Vonderlin et al. 2020; Michaelsen et al. 2023; Dawson et al. 2019) have examined contemplative practice programs in workplace and higher education settings. However, these reviews provide insufficient guidance for future trials seeking to investigate the effects of MBPs on work performance and fail to offer a comprehensive synthesis of MBPs' effects. All existing reviews were designed to capture mental health outcomes rather than work performance, which likely resulted in the omission of studies assessing work performance without mental health measures. The realist review (Micklitz et al. 2021) explored how MBPs work, not their effectiveness, and one review provided only a descriptive summary of findings (Lomas et al. 2017). Four reviews (Bartlett et al. 2019; Vonderlin et al. 2020; Michaelsen et al. 2023; Dawson et al. 2019) extracted work outcomes‐related data when available, yet only two—Vonderlin and colleagues (2020) and Michaelsen and colleagues (2023)—conducted meta‐analyses. The remaining reviews cited high measurement heterogeneity (Bartlett et al. 2019) or an insufficient number of studies (Dawson et al. 2019). The existing meta‐analyses suggest that contemplative practices yield small‐to‐moderate improvements in work engagement (Vonderlin et al. 2020; Michaelsen et al. 2023) and productivity (Vonderlin et al. 2020; Michaelsen et al. 2023) when compared to combined passive and active control conditions. However, these findings are derived from a limited number of studies, which reduces the overall confidence in the estimates. Additionally, Michaelsen and colleagues reported a substantial effect on absenteeism (Michaelsen et al. 2023), although the direction of this effect remains unclear.

While these findings are encouraging, the extent to which they specifically reflect effects of MBPs remains uncertain. To begin with, existing analyses have conflated passive and active control conditions, which may lead to overestimation of intervention efficacy when compared with alternative interventions and underestimation when compared with passive control groups. Additional limitations of prior meta‐analyses include: (a) the absence of a clear definition of work performance; (b) exclusive focus on occupational settings; and (c) inconsistent criteria for intervention inclusion.

To begin with, none of the four meta‐analyses provided an explicit definition of work performance, which likely resulted in the omission of certain indicators, such as adaptive performance. To address this gap, we adopt a theory‐driven framework developed by Koopmans and her colleagues (Koopmans et al. 2011), which conceptualises work performance across four domains: 1) task performance—the quantity and quality of completed tasks assigned to the individual; 2) contextual performance—individual behaviour that supports the organisational environment (including its social and psychological aspects), such as proactivity, cooperating with others, and engagement in work; 3) adaptive performance—the ability to adapt to changes in the organisation or in one's organisational role; 4) counterproductive work behaviour—individual behaviours that are harmful to the organisation, such as absenteeism, presenteeism, misusing privileges, or disregard for safety. Use of Koopmans and colleagues' framework allows us to examine conceptualisations of work performance that have not been previously captured.

Moreover, the majority of the systematic reviews focussed on interventions delivered in the occupational setting (Micklitz et al. 2021; Lomas et al. 2017; Bartlett et al. 2019; Vonderlin et al. 2020). We chose to include a wide selection of intervention delivery settings. In addition to the interventions offered in the community, we include MBPs delivered in higher education, where performance is consistently measured. While university grading systems vary across countries and universities, the diversity is likely to be smaller than across different job roles (e.g., between strawberry pickers vs. theoretical physicists). Students receive marks based on their ability to synthesise attained knowledge, write essays and reports. Such tasks, by design, are akin to those performed by knowledge economy workers (e.g., journalists, researchers, analysts). The inclusion of academic setting could thus improve the external validity of MBPs' effects on work performance. Moreover, an external rater primarily performs academic grading, whereas workplace performance is predominantly measured through self‐reports. In the context of randomised controlled trials (RCTs), independent graders can be kept blind to the treatment allocation (i.e., whether the student completed an MBP) reducing the incidence of expectancy bias.

Finally, there was considerable heterogeneity in the existing reviews regarding what constitutes an MBP. Most commonly, an MBP is defined following Crane and her colleagues' proposal (2017). While some systematic reviews have adopted this (Micklitz et al. 2021; Dawson et al. 2019), in others, the criteria for MBPs are vague (e.g., interventions that were “explicitly described as mindfulness programmes” (Bartlett et al. 2019), or were a “mindfulness‐based program” (Vonderlin et al. 2020). Consequently, prior systematic reviews, and, importantly, the two meta‐analyses that included work performance measures (Vonderlin et al. 2020; Michaelsen et al. 2023), have compassed a wide array of contemplative practices such as breathing exercises, Zen meditation, contemplative training, yoga and mindfulness, transcendental meditation, mindful art processing, or mantra meditation. Given there is limited comparative research on the relative effectiveness of these different contemplative practices (Galante and Van Dam 2024), it is important that evidence syntheses use a clear definition of MBPs to guide future implementation within the workplace to consolidate the field. As outlined earlier and detailed in the eligibility criteria, we have adhered to the definition of MBPs proposed by Crane and colleagues (2017). This approach ensures that our review focuses specifically on the effects of MBPs, rather than on other contemplative practices that may incorporate elements of meditation or mindfulness.

To guide future trials evaluating mindfulness‐based programs (MBPs) in workplace settings, it is essential to systematically map how work performance has been assessed in studies investigating the effectiveness of MBPs, as defined by Crane and colleagues (2017), across different domains of work performance (Koopmans et al. 2011). Understanding these effects is critical for informing organisational investment decisions, guiding policy, the development of MBPs, and clarifying whether MBPs deliver benefits beyond general well‐being improvements. To our knowledge, the present systematic review and meta‐analysis is the first to synthesise evidence on MBPs' effects on work performance using a theoretical framework for the independent as well as dependent variables. Expanding the evaluation of MBPs to encompass all facets of work performance is not only critical for advancing the scientific evidence base but also for ensuring that organisational investments in training and well‐being programmes are used effectively. Accordingly, we conducted a rigorous, pre‐registered systematic review and meta‐analysis of MBPs—defined according to a widely accepted consensus—categorising outcomes using an established framework and applying clear, inclusive criteria. This comprehensive evaluation is vital to determine whether the substantial resources allocated to MBPs deliver meaningful benefits for individuals and generate economic and social value for organisations (Van Dam et al. 2017).

Our primary research question was: what is the effectiveness of MBPs, compared with no intervention or comparator interventions, for improving work performance within the first month following completion of the intervention? Our secondary aim was to explore features of MBP delivery and trial design that may influence their effects, to guide further research and real‐world implementation. Specifically, we sought to evaluate (a) which measures of individual performance are used in MBP trials; (b) whether effect sizes differ between externally rated performance (e.g., employer or institutional metrics) and self‐reported ratings; (c) the comparative effectiveness of shorter versus longer MBPs; (d) the duration of MBP effects on individual work performance; (e) whether effects of MBPs vary across settings; (f) gaps in the evidence and areas for further research.

## 2 Methods

This study has followed fully pre‐registered methods (Vainre et al. 2020) and is reported according to the Preferred Reporting Items for Systematic Reviews and Meta–Analyses (PRISMA) 2020 guidelines (Page et al. 2021).

### 2.1 Eligibility Criteria

A study was eligible to be included if it (a) was published in a peer‐reviewed journal; (b) was published either in Catalan, English, Estonian, French, German, Italian, Portuguese, or Spanish (the languages spoken by the research team); (c) was a randomised controlled trial (RCT); (d) evaluated a secular MBP as defined by Crane and her colleagues (2017) with a minimum duration of 4 h (Ma and Teasdale 2004; Teasdale et al. 2000); (e) delivered synchronously or asynchronously, regardless of the medium (in‐person, online, pre‐recorded, such as an app or a book); (f) included participants who were at least 17 years old, living in the community and who were not selected for having any particular health status (e.g., a health problem, health risk (substance abuse), or pregnancy); (g) reported to have collected at least one outcome of interest (see below for details); (h) compared an MBP with at least one control group that did not receive an eligible MBP as defined above (studies that only compared two eligible MBPs with no other arms were excluded).

We excluded residential programmes, for example, retreat‐based interventions, as they are unlikely to be used in organisational settings and may be qualitatively different from non‐residential programmes. We also excluded other contemplative practice programmes that would not adhere to the consensus of what MBPs are according to Crane and her colleagues (2017).

### 2.2 Information Sources and Search Strategy

We searched the following databases for eligible records: ASSIA, EMBASE (via Ovid), ERIC (via EBSCOhost), MEDLINE (via Ovid), PsycINFO (via EBSCOhost), Scopus, Web of Science, and Cochrane Central Register of Controlled Trials (CENTRAL). The search was conducted on 2^nd^ August 2024. We applied no restrictions to the results provided by the search terms (see Supplementary Materials 1) and searched for studies published since the inception of the database. Additionally, we searched for references in the existing systematic reviews (Micklitz et al. 2021; Lomas et al. 2017; Bartlett et al. 2019; Vonderlin et al. 2020; Dawson et al. 2019). To minimise publication bias, we searched for unpublished but completed trials pre‐registered in the World Health Organization (WHO) International Clinical Trials Registry Platform. We considered unpublished studies registered a minimum of 3 years before our search date as an indication of potential publication bias.

### 2.3 Selection and Data Extraction

Retrieved records were imported into EndNote and duplicates were removed. Records were then exported and fed into a machine learning programme to identify reports of RCTs (Marshall et al. 2018) using the sensitive setting recommended for systematic reviews. The output was uploaded to Rayyan (Ouzzani et al. 2016) where each title and abstract were assessed by two independent reviewers against inclusion criteria. The eligibility of full texts was evaluated when the titles and abstracts were rated relevant by at least one of the reviewers. Multiple reports of the same trial were combined. Data were extracted from the included full‐text reports by two independent researchers using pre‐piloted forms set up in Covidence (see Supplementary Materials 2). Disagreements at any stage were discussed and resolved within the review team.

### 2.4 Outcomes and Comparisons

The primary outcome was task performance (e.g., work quality or quantity), as described in Koopmans and colleagues (2011). Secondary outcomes were measures that could be categorised into the three remaining domains of the model, that is, measures of contextual performance, adaptive performance, and counterproductive behaviours. The choice of the outcome to be extracted followed the hierarchy pre‐specified in the protocol (Vainre et al. 2020). When choosing the outcomes, we based our decision on the intrinsic value, not the specific context in which it was collected. For example, writing creativity, a measure used in Bellosta‐Batalla and colleagues (2021), may have a specific value in certain professions (journalists, writers) while less pertinent in others (a student population). We ignored the context and extracted the outcome if it was deemed to contribute to work performance according to the Koopmans and colleagues' (2011) model. Outcomes deemed not to belong to any of the four outcome domains were excluded from the review. All outcomes were second‐rated by MV, who also categorised them. Where there were initial disagreements between the raters, they were resolved by consulting with JG who was blind to the study outcomes.

Task performance measures taken at post‐programme were considered the primary outcome. Where time since the end of the MBP programme was reported, we considered data collected up to 4 weeks after completion of the programme as our primary outcome time point. Where authors described their measure as collected post‐programme, we considered it to belong to the primary outcome. Secondary outcomes time periods were task performance measures collected a) between 5 and 24 weeks post‐programme and b) at ≥ 25 weeks after the end of the programme. Where outcomes were measured more than once within these pre‐specified time ranges, the longest follow‐up was used. Where reported, we also extracted the baseline outcome values for inclusion in analysis, as recommended by Clifton and Clifton (2019). If multiple measurements were taken prior to randomisation, we selected the assessment closest to the point of randomisation.

Control groups were categorised as follows to align with recent similar reviews (Dunning et al. 2018; Galante et al. 2021; Goyal et al. 2014): (a) studies using either no contact or wait‐list groups (i.e., passive controls); (b) control interventions designed principally to take account of non‐specific factors of the intervention studied (i.e., placebo controls); (c) control interventions with active ingredients specifically designed to drive change in one or more outcomes of interest (i.e., active intervention controls).

### 2.5 Risk of Bias and Confidence in Results

Two reviewers independently assessed the quality and risk of bias of all included studies using the Risk of Bias tool (second version; RoB2) developed by the Cochrane Collaboration (Sterne et al. 2019). We made one deviation from the original RoB2: for the risk of the reported result, we rated the risk of bias as high when no information was available on any of the three items. The assumptions made during the rating are described in Supplementary Materials 2. The decisions were recorded using pre‐piloted forms set up in Covidence (Supplementary Materials 2). Disagreements were resolved through discussion within the research team.

Additionally, we collected information about allegiance and funding. Allegiance was considered a risk of bias when the authors of the paper designed the intervention or delivered it. Funding was considered a risk of bias where the organisation delivering the MBPs funded or conducted the study. The GRADE approach was used to assess overall quality of the synthesised evidence (Guyatt et al. 2008). We investigated publication bias with funnel plots.

### 2.6 Synthesis Methods

We used the meta package (Balduzzi et al. 2019) in *R* v4.3.3 (R Core Team 2024) run in RStudio v2024.04.02 to conduct pairwise random‐effects meta‐analyses within the four work performance domains and within the three comparator categories (passive, placebo, and active intervention controls). We set the *α*‐level at 0.05 for primary as well as secondary outcomes. We treated the secondary outcomes' meta‐analyses as exploratory and thus did not correct the *α*‐level for multiple comparisons. Where a multi‐armed trial compared several eligible MBPs with a non‐MBP control group, the MBPs intervention groups were combined (Higgins et al. 2022a).

We used Hedges' *g* (also known as standardised mean difference, SMD) to index intervention effects as trials used different instruments to measure outcomes. Where baseline outcome values were reported, we calculated Hedges' *g* using the ANCOVA estimate (Mackenzie et al. 2006). The within‐study baseline‐post‐intervention correlations needed to calculate the ANCOVA estimate were calculated based on published results for task performance (Allexandre et al. 2016; Garrote‐Caparrós et al. 2022; Lebares et al. 2019; Nübold et al. 2021; Vainre et al. 2024) and adaptive performance (Lebares et al. 2019; Aikens et al. 2014; Asuero et al. 2014; Fazia et al. 2023; Nadler et al. 2020; Nielsen et al. 2021; Roeser et al. 2022). For the other two domains, we averaged correlations across the studies for lack of a better alternative. For the 5–24 weeks post‐intervention period, task performance correlations (Allexandre et al. 2016; Garrote‐Caparrós et al. 2022; Vainre et al. 2024) and adaptive performance correlations (Aikens et al. 2014; Nielsen et al. 2021; Bonde et al. 2022) were calculated from published results. We used the average correlations calculated based on their data in all four outcome domains.

Where baseline data were missing, we calculated the Hedges' *g* based on unadjusted final values. Ordinal and categorical data were transformed to Hedges' *g*s using approaches set out in the Cochrane Handbook (Higgins et al. 2022b). Subscales were combined by pooling their means and standard deviations. Where arm‐specific sample sizes were missing, we divided the total sample size equally between the arms. The sample sizes for cluster‐RCTs were adjusted for the meta‐analysis (Higgins et al. 2022a) using an intraclass correlation (ICC) of 0.05 (Galante et al. 2021).

Estimation of heterogeneity was performed using the restricted maximum likelihood method. Confidence intervals for the overall mean were estimated with the modified Hartung, Knapp, Sidik and Jonkman method (Knapp et al. 2003; Röver et al. 2015) as well as the *I* ^2^ statistic and prediction intervals were calculated using the *metagen* function in the *meta* package (Balduzzi et al. 2019).

We also conducted pre‐specified analyses to investigate heterogeneity on the primary outcome. For continuous variables (duration of the intervention), we conducted meta‐regression analyses. For categorical variables (delivery setting, reporter type) we used sub‐group analyses. To investigate the effect of MBP duration on the outcome, we converted the duration into hours of guided content. The duration of self‐help MBPs was calculated by multiplying the duration of guided meditation multiplied by that number of days per week the participants were asked to practice meditation and the number of weeks the intervention was intended to last. We excluded the duration of unguided mediation, as studies rarely quantified its duration. For face‐to‐face or other human‐taught synchronously delivered programmes, we only included synchronously delivered sessions to estimate duration, that is, leaving out independent home practice.

### 2.7 Amendments to the Protocol

Compared to the original protocol, we decided to exclude theses and outcomes measuring burn‐out and work‐related stress. These decisions were made during full‐text screening due to limited capacity and in order to limit the scope of this review. For the primary outcome's sub‐group analyses, we had planned to analyse reporter type categorised either as self‐reported, reported by someone else, or routinely collected. Due to the small number of eligible studies, we grouped non‐self‐reported outcomes together and thus compared self‐report to non‐self‐report. We had also planned to run sensitivity analyses on the main outcome by excluding high risk of bias studies. However, as all studies contributing data to the main outcome were deemed of high risk of bias, this analysis was not possible. Finally, we also planned to do a sub‐group analysis comparing organisational and community settings. There were not enough community samples, so the sub‐group analysis compares MBP delivered at work and in educational settings.

## 3 Results

### 3.1 Study Selection

**FIGURE 1:**
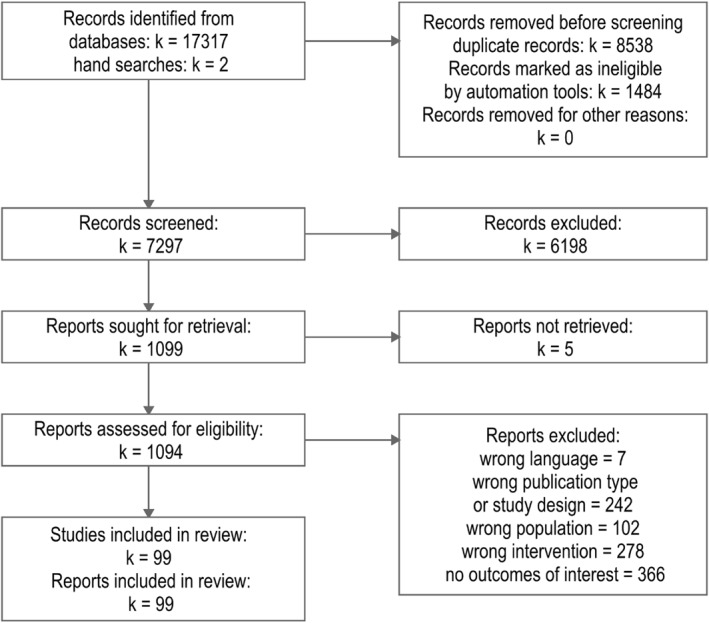
PRISMA 2020 Flowchart for study selection. For eligibility criteria, please refer to the methods section.

The study selection flowchart is shown in Figure 1. In total, 99 trials were included in this review. We contacted authors of six studies for which we were unable to retrieve full‐text reports to assess their eligibility. One author responded.

### 3.2 Study Characteristics

Table 1 summarises the characteristics of the 99 studies included in the review. 11 were cluster‐RCTs (Bonde et al. 2022; Augustus et al. 2024; Fraiman et al. 2022; Hubert et al. 2022; Hwang and Jo 2019; Janssen et al. 2022; Kuyken et al. 2022; Nassif et al. 2023 (study 1 and 2); Pérula‐de Torres et al. 2021; van Dijk et al. 2017), and three studies (Nielsen et al. 2021; dos Santos et al. 2024; Glass et al. 2019) featured a cross‐over design, where we extracted data up to the cross‐over time point. The trials were carried out in 27 countries and published between 1998 and 2024. A total of 16,054 participants (min = 18, max = 2182, median = 92) took part in the trials. Most of them identified as female (70.1%). The mean age of participants per study ranged from 18.0 to 63.6 years. Most studies recruited employees (*k* = 63) or students (*k* = 29) as participants. The remaining 7 studies were conducted in other settings (e.g., carers in a community setting). Passive control groups were used in 71 studies. The remaining 28 used either active specific control groups (*k* = 11), active non‐specific (i.e., placebo) control groups (*k* = 9), or used both, a passive control and an active control group (*k* = 8). One study was published in Spanish (Gómez‐Odriozola et al. 2019).

**TABLE 1:** Study characteristics.

First author (year), country	Participants	*n*	Females %^a^	Age (years): M(SD)^a^	Intervention(s)^b^	Duration weeks(hrs)^c^	Control^b^	Timepoints^d^
Aikens et al. (2014), USA	Employees	90	NR	NR	Dow mindful resilience programme^, remote/hybrid^	NR w (7 h)+	Waitlist	BL, < 4 w, 5–24 w
Allexandre et al. (2016), USA	Employees	161	83%	40 (12.6)	Web‐based stress management (WSM)^Individual + group, live + self‐paced^	8 w (9.5 h)+	Waitlist	BL, < 4 w, 5–24 w
AlQarni et al. (2023), Saudi Arabia	Employees	147	63%	33 (7)	Unnamed MBP^Individual, self‐paced^	2 w (4.7 h)+	Progressive muscle relaxation	BL, < 4 w
Asthana et al. (2021), Slovenia	Students	310	NR	35.55 (6.51)	Mindfulness programme for MBA^F2F^	20 w (46.2 h)+	No contact or waitlist control	BL, < 4 w
Asuero et al. (2014), Spain	Employees	68	NR	48.1 (7.42)	MBSR adaption^F2F^	8 w (28 h)+	Waitlist	BL, < 4 w
Augustus et al. (2024), USA^C^ ^−RCT^	Students	58	NR	19.71 (1.34)	Unnamed MBP^F2F^	7 w (7 h)+	Waitlist	BL, < 4 w, 5–24 w
Balci et al. (2023), Germany, Switzerland, and Austria	Students	40	78%	26.23 (4.5)	StudiCareM‐E^Individual, self‐paced^	7 w (7.6 h)	Info on support offers	BL, < 4 w
Barczak‐scarboro et al. (2021), USA	Other	231	56%	48.53 (8.95)	Unnamed MBP^F2F^	6 w (6 h)+	Loving‐kindness meditation	BL, < 4 w, 5–24 w, > 24 w
Bartlett et al. (2017), Australia	Employees	133	74%	NR	Mindfulness at work programme^F2F^	5 w (7.5 h)+	Self‐help information resources	BL, < 4 w
Baumgartner et al. (2023), USA	Students	128	67%	20 (4.44)	MBSR adaption^F2F^	7 w (10.5 h)+	1. Study skills and 2. Waitlist	5–24 w
Bellosta‐Batallaet al. (2021), Spain	Students	68	72%	23.6 (5.43)	Mindfulness and compassion^F2F^	8 w (16 h)+	Waitlist	BL, < 4 w, 5–24 w
Benn et al. (2012), USA	Employees	38	84%	45.6 (NR)	SMART‐in‐education^F2F^	5 w (36 h)+	Waitlist	BL, < 4 w, 5–24 w
Bonde et al. (2022), Denmark^C^ ^−RCT^	Employees	191	92%	45.2 (8.4)	MBSR^F2F^	8 w (27 h)+	Waitlist	BL, 5–24 w
Braun et al. (2020a), Canada and USA	Employees	171	82%	45 (9.44)	Mindfulness‐based emotional balance (MBEB)^F2F^	9 w (36 h)+	Waitlist	5–24 w, > 24 w
Braun et al. (2020b), USA	Students	48	92%	25.96 (5.09)	Mindfulness for interdisciplinary healthcare professionals (MIHP)^F2F^	8 w (16 h)+	Waitlist	BL, < 4 w
Brown et al. (2016), USA	Other	38	84%	61.14 (10.41)	MBSR adaption^F2F^	8 w (19 h)	Alzheimers' association‐sponsored social support	BL, < 4 w, 5–24 w
Calcagni et al. (2021), Spain	Employees	75	NR	41.3 (10.03)	Combination of MBCT adaption and mindfulness and positive stress management^F2F^	4.5 w (15 h)+	Waitlist	BL, < 4 w
Can Gür et al. (2020), Turkey	Students	123	70%	21.08 (2.18)	Mindfulness‐based empathy training^F2F^	8 w (NR hrs)+	No contact	BL, < 4 w
Chan et al. (2021), Hong Kong	Students	50	60%	NR	MBCT^F2F^	8 w (16 h)+	Waitlist	BL, 5–24 w
Choi et al. (2022), Canada	Employees	230	94%	NR	MBSR adaption^F2F^	8 w (NR hrs)	1. Pilates and 2. Waitlist	BL, < 4 w
Choi et al. (2024), Canada	Employees	230	94%	NR	Unnamed MBP^F2F^	8 w (8 h)+	1. Pilates and 2. Waitlist	BL, < 4 w
Christodoulou et al. (2022), UK	Employees	240	77%	42 (10.4)	MBSR adaption^F2F^	4 w (4 h)+	1. Acceptance and commitment therapy and 2. Waitlist	BL, < 4 w, 5–24 w
Christopher et al. (2018), USA	Employees	61	11%	43.99 (6.07)	Mindfulness‐based resilience training (MBRT)^F2F^	8 w (22 h)+	No contact	BL, < 4 w, 5–24 w
Christopher et al. (2024), USA	Employees	73	30%	38.94 (8.94)	Mindfulness‐based resilience training (MBRT)^, remote/hybrid^	8 w (15 h)+	1. Stress management education (SME) and 2. Other	BL, < 4 w, 5–24 w
de Carvalho et al. (2021), Portugal	Employees	228	NR	NR	Atentamente^F2F^	10 w (30 h)	Waitlist	BL, < 4 w
Daigle et al. (2018), Canada	Employees	75	NR	46.21 (9.6)	MBSR^F2F^	8 w (28 h)+	Waitlist	5–24 w
de Jong et al. (2013), The Netherlands	Employees	60	43%	46.67 (8.11)	MBSR^F2F^	8 w (20 h)+	No contact	BL, < 4 w
Desai et al. (2024), USA	Employees	81	88%	45 (11.96)	Heartfulness meditation^Individual + group, remote/hybrid^	6 w (22.5 h)	Gratitude practice group	BL, < 4 w
dos Santos et al. (2024), Brazil	Employees	35	100%	42.6 (9.2)	Modified breathworks mindfulness for health (BMfH)^, remote/hybrid^	8 w (12 h)	Waitlist	BL, < 4 w
Dvořáková et al. (2017), USA	Students	109	66%	18.2 (0.4)	Learning 2 breathe^F2F^	6 w (10.7 h)+	Waitlist	BL, < 4 w
Erden et al. (2023), Turkey	Employees	42	100%	36.38 (3.81)	MBSR^F2F^	8 w (6 h)	Waitlist	BL, < 4 w
Erogul et al. (2014), USA	Students	81	32%	23.44 (1.66)	MBSR^F2F^	8 w (15 h)+	Wailist	BL, < 4 w, 5–24 w
Fazia et al. (2023), Italy	Students	530	52%	22.88 (3.66)	Integral meditation^, remote/hybrid^	5 w (5.8 h)	Waitlist	BL, < 4 w, 5–24 w
Flook et al. (2013), USA	Employees	18	89%	43.06 (9.87)	MBSR adaption^F2F^	8 w (26 h)+	Waitlist	BL, < 4 w
Fraiman et al. (2022), USA^C^ ^−RCT^	Employees	359	71%	NR	Mindfulness intervention for new interns (MINdI)^F2F^	26 w (7 h)+	1‐h Social lunches	BL, < 4 w, > 24 w
Galante et al. (2018), UK	Students	616	63%	NR	Mindfulness skills for students (MSS)^F2F^	8 w (10.2 h)+	SAU	5–24 w
Garrote‐Caparróset al. (2022), Spain	Employees	63	86%	NR (9.4)	Mindfulness and compassion‐based intervention (MCBI)^F2F^	8 w (16 h)+	Empathy diary	BL, < 4 w, 5–24 w
Glass et al. (2019), USA	Students	57	77%	19.32 (1.25)	Mindful sport performance enhancement^F2F^	6 w (7.5 h)+	Waitlist	BL, < 4 w, 5–24 w, > 24 w
Godara et al. (2024), Germany	Other	253	75%	43.66 (11.59)	CovSocial app^Individual + group, live + self‐paced^	10 w (25 h)+	1. Socio‐emotional training (affect dyad) and 2. Waitlist	BL, < 4 w
Grupe et al. (2021), USA	Employees	114	41%	40 (8.37)	Unnamed MBP^F2F^	8 w (18 h)+	Waitlist	BL, < 4 w, 5–24 w
Gómez‐Odriozolaet al. (2019), Spain	Students	114	81%	17.99 (0.69)	Learning 2 breathe^F2F^	6 w (6 h)+	Waitlist	BL, < 4 w
Hillhouse et al. (2023), USA	Employees	56	NR	45.16 (NR)	Unnamed MBP^F2F^	4 w (13 h)+	Not specified	BL, < 4 w
Huberty et al. (2022), USA^C^ ^−RCT^	Employees	1029	50%	NR	Calm app^Individual, self‐paced^	8 w (9.3 h)	Waitlist	BL, < 4 w
Hunsinger et al. (2019), USA	Employees	61	10%	43.97 (6.03)	MBSR adaption^F2F^	8 w (16 h)+	No contact	BL, < 4 w, 5–24 w
Hwang et al. (2019), Australia^C^ ^−RCT^	Employees	185	NR	43.08 (11.59)	Reconnected^F2F^	8 w (12 h)+	SAU	BL, < 4 w, 5–24 w
Janssen et al. (2022), The Netherlands^C^ ^−RCT^	Employees	120	71%	49 (11.34)	MBSR adaption^F2F^	8 w (20 h)+	Waitlist	BL, < 4 w, 5–24 w, > 24 w
Jennings et al. (2013), USA	Employees	53	89%	36 (NR)	Cultivating awareness and resilience in education (CARE)^F2F^	5 w (36 h)+	Waitlist	BL, < 4 w
Jennings et al. (2017), USA	Employees	224	93%	NR	Cultivating awareness and resilience in education (CARE)^F2F^	16 w (30 h)+	Waitlist	BL, 5–24 w
Jia‐Yuan et al. (2022), China	Students	72	61%	19.14 (1.81)	Mindfulness‐based emotion management programme^, remote/hybrid^	4 w (5.3 h)+	Waitlist	BL, < 4 w, 5–24 w
Juul et al. (2021), Denmark	Students	67	73%	26.34 (4.12)	MBSR^F2F^	8 w (27 h)+	Waitlist	BL, 5–24 w
Karing et al. (2021), Germany	Students	71	80%	22.68 (3.26)	Unnamed MBP^F2F^	6 w (4 h)+	Waitlist	BL, < 4 w, 5‐24 w
Klatt et al. (2015), USA	Employees	34	NR	NR	Mindfulness in motion^F2F^	8 w (8 h)+	Waitlist	BL, < 4 w
Klatt et al. (2017), Denmark	Employees	81	NR	42.91 (9.29)	Mindfulness in motion^F2F^	8 w (8 h)+	Waitlist	BL, < 4 w, 5–24 w
Kor et al. (2019), Hong Kong	Other	36	83%	57.1 (10.6)	MBCT adaption^F2F^	10 w (14 h)+	Education and support	BL, < 4 w, 5‐24 w
Kor et al. (2020), Hong Kong	Other	113	61%	61.7 (10.5)	MBCT adaption^F2F^	10 w (14 h)+	SAU + brief education session	BL, < 4 w, 5–24 w
Kuyken et al. (2022), UK^C^ ^−RCT^	Employees	679	75%	39.7 (9)	MBCT for life + 4‐day workshop^F2F^	8 w (46 h)+	SAU	BL, < 4 w, 5–24 w, > 24 w
Küchler et al. (2023), Germany	Students	386	75%	25.77 (5.34)	StudiCare mindfulness^Individual, self‐paced^	7 w (7.9 h)+	Waitlist	BL, < 4 w, 5–24 w
Lebares et al. (2019), USA	Employees	21	38%	28.24 (2.35)	MBSR adaption^F2F^	8 w (16 h)+	Reading and discussion on becoming a surgeon	BL, < 4 w, > 24 w
Lensen et al. (2024), The Netherlands	Employees	146	85%	39.4 (11.3)	MBSR^,F2F + self‐paced^	8 w (NR hrs)	Waitlist	BL, < 4 w, 5–24 w
Lin et al. (2019), China	Employees	110	76%	31.53 (6.92)	Unnamed MBP^F2F^	8 w (16 h)+	Waitlist	BL, < 4 w, 5–24 w
Liu et al. (2022), China	Employees	91	59%	39.71 (7.82)	Unnamed MBP^F2F^	8 w (12 h)+	Waitlist	BL, < 4 w
Liu et al. (2023), China	Employees	90	99%	38.11 (7.81)	Unnamed MBP^, remote/hybrid^	8 w (14 h)+	Waitlist	BL, < 4 w
Modrego‐Alarcón et al. (2021), Spain	Students	280	79%	22.25 (5.75)	Unnamed MBP^Individual + groupF2F^	6 w (8.2 h)	Relaxation therapy	BL, < 4 w, 5–24 w
Nadler et al. (2020), USA	Employees	275	27%	NR	MBSR adaption^Individual, self‐paced^	8 w (1.6 h)+	Waitlist	
Nassif et al. (2023) study 1, USA^C^ ^−RCT^	Employees	121	0%	NR	Mindfulness‐based attention training (MBAT)^F2F^	4 w (8 h)+	Waitlist	BL, < 4 w, 5–24 w
Nassif et al. (2023) study 2, USA^C^ ^−RCT^	Employees	77	12%	NR	Mindfulness‐based attention training (MBAT)^F2F^	4 w (8 h)+	Waitlist	BL, < 4 w, 5–24 w
Nielsen et al. (2021), USA	Employees	100	63%	47.14 (9.61)	Mindful pause^Individual, live + self‐paced^	8 w (NR hrs)	Waitlist	BL, < 4 w, 5–24 w
Nübold et al. (2021), Europe	Employees	162	60%	34.04 (10.87)	Headspace app^Individual, self‐paced^	4.3 w (5 h)	1. Brain‐training by Memorado and 2. Waitlist	BL, < 4 w
Ogino et al. (2024), Japan	Employees	300	38%	42.27 (9.1)	Mindfulness and compassion against COVID‐19 (IMACOCO)^, remote/hybrid^	8 w (9 h)	Waitlist	BL, < 4 w
Orosa‐Duarte et al. (2021), Spain	Students	154	85%	23 (4.16)	Combination of going home and MBSR adaption^Individual + group, live + self‐paced^	8 w (11.7 h)+	Waitlist	BL, < 4 w
Pang et al. (2019), Switzerland	Employees	63	69%	44.2 (10)	Combination of mindfulness‐based strengths practice and MBSR^F2F^	8 w (16 h)+	Waitlist	BL, < 4 w, 5–24 w
Perez‐Blasco et al. (2016), Spain	Other	45	67%	63.56 (4.1)	Unnamed MBP^F2F^	10 w (20 h)+	Waitlist	BL, < 4 w
Phang et al. (2015), Malaysia	Students	75	76%	21.04 (1.13)	Mindful‐gym^F2F^	5 w (10 h)+	Waitlist	BL, < 4 w, 5–24 w
Pipe et al. (2009), USA	Employees	33	94%	49.79 (6.75)	MBSR adaption^F2F^	4 w (10 h)+	Attention control	BL, < 4 w, > 24 w
Rad et al. (2023), Iran	Students	36	36%	20.59 (0.89)	Unnamed MBP^F2F^	8 w (12 h)+	Waitlist	BL, < 4 w
Repo et al. (2022), Finland	Students	102	73%	NR	MBCT adaption^F2F^	8 w (10.2 h)+	1. Acceptance and Commitment therapy and 2. Waitlist	BL, < 4 w, 5–24 w
Rich et al. (2021), UK	Employees	125	70%	NR	Headspace app^Individual, self‐paced^	6.4 w (32.1 h)+	Waitlist	BL, < 4 w
Rodrigues de Oliveira et al. (2021), Brazil	Employees	76	100%	44.71 (8.4)	Mindfulness‐based health program for educators (MBHP‐Educa)^F2F^	8 w (16 h)+	Neuroscience for education program (neuro‐educa)	BL, < 4 w, > 24 w
Roeser et al. (2013), USA and Canada	Employees	113	88%	46.9 (9.2)	Mindfulness training crogram^F2F^	8 w (36 h)+	Waitlist	BL, 5–24 w, > 24 w
Roeser et al. (2022), USA	Employees	58	69%	41.23 (8.66)	Mindfulness‐based emotional balance (MBEB)^F2F^	8 w (28 h)+	Waitlist	BL, < 4 w, 5–24 w
Sampl et al. (2017), Austria	Students	109	75%	22.28 (4.55)	Mindfulness‐based self‐leadership training (MBSLT)^F2F^	10 w (20 h)+	Waitlist	BL, < 4 w
Schroeder et al. (2018), USA	Employees	33	73%	42.76 (8.43)	Mindful medicine curriculum (MMC)^F2F^	2 w (17 h)	Waitlist	BL, < 4 w, 5–24 w
Shapiro et al. (1998), USA	Students	78	53%	NR	Stress reduction and Relaxation^F2F^	7 w (17.5 h)+	Waitlist	BL, < 4 w
Shapiro et al. 2011), USA	Students	32	81%	18.73 (1.29)	Combination of MBSR and easwaran eight‐point program (EPP)^F2F^	8 w (NR hrs)+	Waitlist	BL, < 4 w, 5–24 w, > 24 w
Shapiro et al. (2019), USA	Students	41	122%	24.4 (NR)	MBSR adaption^F2F^	8 w (12.5 h)+	Introductory class to mindfulness	BL, < 4 w
Steinberg et al. (2017), USA	Employees	32	NR	39.8 (NR)	Unnamed MBP^F2F^	8 w (8 h)+	Waitlist	BL, < 4 w
Strauss et al. (2021), UK	Employees	234	83%	43.95 (10.4)	MBCT adaption^F2F^	8 w (16 h)+	Waitlist	BL, < 4 w
Takhdat et al. (2024), Morocco	Students	92	51%	22.11 (1.7)	MBSR adaption^F2F^	4 w (8 h)	Not specified	< 4 w, > 24 w
Taylor et al. (2016), Canada	Employees	59	90%	NR	Unnamed MBP^F2F^	9 w (36 h)+	Waitlist	BL, < 4 w, 5–24 w
Taylor et al. (2022), UK	Employees	2182	83%	40.53 (10.97)	Headspace app^Individual, self‐paced^	17.9 w (20.8 h)	NHS moodzone	BL, < 4 w
Pérula‐de Torres et al. (2021), Spain^C^ ^−RCT^	Employees	165	52%	41.61 (12.61)	MBSR + mindful Self‐compassion^F2F^	6 w (15 h)+	Waitlist	BL, < 4 w, 5–24 w
Vainre et al. (2024), UK	Employees	241	85%	44.62 (10.67)	Be mindful^Individual, self‐paced^	4 w (5.2 h)+	Light physical exercise	BL, < 4 w, 5–24 w
Valley et al. (2017), USA	Employees	23	87%	NR	MBSR^F2F^	8 w (27 h)+	Waitlist	BL, < 4 w, > 24 w
van Dongen et al. (2016) and van Berkel et al. (2014), The Netherlands	Employees	257	NR	45.55 (9.49)	Vitality in practice^F2F^	8 w (12 h)+	E‐mail with a link to a webpage on health promotion	BL, 5–24 w, > 24 w
van Dijk et al. (2017), The Netherlands^C^ ^−RCT^	Students	167	78%	23.5 (1.85)	MBSR adaption^F2F^	8 w (16 h)+	SAU	< 4 w, 5–24 w, > 24 w
Verweij et al. (2018), The Netherlands	Employees	148	88%	31.2 (4.6)	MBSR^F2F^	8 w (26 h)+	Waitlist	BL, 5–24 w
Wang et al. (2023), China	Employees	118	74%	31.85 (3.79)	Guided self‐help MBP^Individual, self‐paced^	8 w (16.7 h)+	Psychoeducation	BL, < 4 w
Watson‐Singleton et al. (2024), USA	Other	212	43%	36.06 (12.29)	BlackFULLness app^Individual, self‐paced^	12 w (NR hrs)	Waitlist	BL, < 4 w
Wilson et al. (2022), Brazil	Employees	74	100%	44.05 (7.32)	Mindfulness‐based health program for educators (MBHP‐Educa)^F2F^	8 w (16 h)+	Neuroscience for education program (neuro‐educa)	BL, < 4 w

^a^ NR = not reported.

^b^ MBCT = Mindfulness‐Based Cognitive Therapy, MBSR = Mindfulness‐Based Stress Reduction, SAU = service as usual.

^c^ Intervention duration: The symbol '+' denotes additional homework.

^d^ Timepoints: BL = Baseline, < 4 weeks = up to 4 weeks post‐intervention, 5–24 weeks = 5–24 weeks post‐intervention, > 24 weeks = more than 24 weeks post‐intervention. C‐RCT Study design: Cluster‐RCT, Individual Individual, Individual + group Mixed: individual and group‐based, F2F Face‐to‐face, self‐paced Self‐paced, live + self‐paced Mixed synchronous (live) and self‐paced, remote/hybrid Synchronous (live), delivered remotely or hybrid, F2F + self‐paced Mixed face‐to‐face and self‐paced.

The majority of the mindfulness‐based programmes included in this review were delivered face‐to‐face (*k* = 75). The remaining (*k* = 24) were delivered either completely or partially online: 11 were entirely self‐paced e‐programmes (e.g., an app, online course) (Nübold et al. 2021; Vainre et al. 2024; Nadler et al. 2020; Huberty et al. 2022; AlQarni et al. 2023; Balci et al. 2023; Küchler et al. 2023; Rich et al. 2021; Taylor et al. 2022; Wang et al. 2023; Watson‐Singleton et al. 2024), 8 were either hybrid or delivered remotely requiring participants to log on at an arranged time‐slot, and 5 were self‐paced but supported by some synchronous component (e.g., a video call, a group meeting). The programmes' duration ranged from 2 to 26 weeks (*M* = 7.78, SD = 3.19 weeks) and on average included 16.03 h (SD = 9.72) of guided meditation. Almost all studies (*k* = 80) encouraged participants to engage in meditation outside of the guided sessions, this includes self‐paced practice when delivered via an app or an online platform.

Work performance was measured with 91 different measures across the four domains. Task performance was captured with 30 different measures. Contextual performance was measured with 22, Adaptive performance with 24 and the remaining 15 indexed counterproductive work behaviour (see Supplementary Materials 3 Table S4).

#### 3.2.1 Risk of Bias in Studies

**FIGURE 2:**
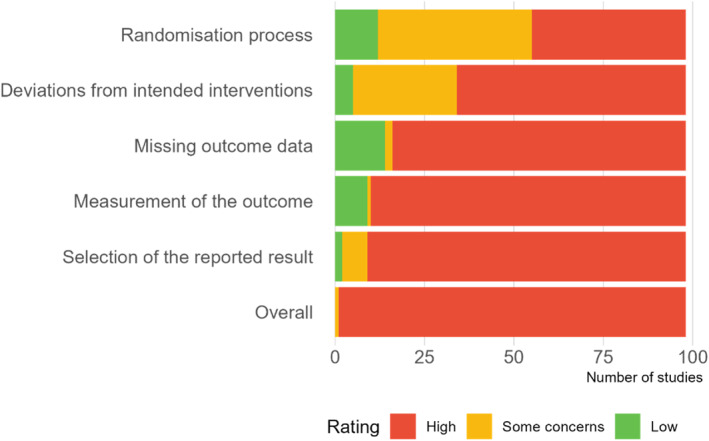
Risk of bias assessment summary.

All studies, except one (Galante et al. 2018) received a high overall risk of bias rating (Figure 2, details in Table S1). Concerns related to risk of bias due to the randomisation process (88% studies) primarily arose because allocation sequence concealment was unclear or because the randomisation method precluded concealment. In the domain assessing the effect of assignment to intervention, elevated risk of bias ratings (95% studies) were attributable to two factor: first, the use of per‐protocol rather than intention‐to‐treat analyses or insufficient transparency regarding the analytic approach, and second, the nature of the intervention—a behavioural programme, rather than a pharmaceutical prescription—made it difficult to conceal allocation from the staff delivering the treatment. Moreover, the characteristics of MBPs inherently prevented participant blinding.

Elevated risk of bias ratings due to missing outcome data (86% studies) were influenced by high attrition rates coupled with lack of sensitivity analyses. In the measurement of the outcome domain, high risk of bias ratings (91% studies) frequently resulted from reliance on self‐report measures or failure to specify whether observers were blinded. These sources of bias are likely to inflate effect size estimates. Most studies (98%) did not have a pre‐registered analysis plan available and thus received a high risk of bias rating in selection of the reported result.

We found some degree of allegiance to the MBP in 61 (63%) studies. That is, the authors either had developed or delivered the intervention. Concerns regarding funding sources were identified in 29 (30%) studies, primarily due to undeclared funding. Twenty‐three studies received a low rating for vested interest due to author allegiance or funding.

### 3.3 Effects of MBPs on Work Performance

Summary statistics for each study's outcome measures of interest are presented in the Supplementary Materials 3 (Tables S6‐S9) for each pre‐specified time period. The detailed results are also presented in Supplementary Materials 3.

### 3.4 Primary Outcome: Task Performance Up to 4 Weeks Post‐Intervention

The primary outcome was collected in 30 studies (*k*_passive_ = 24; *k*_active_ = 4; *k*_placebo_ = 2). Of these, two studies with passive control groups were excluded from this meta‐analysis (Nassif 2023 study 1 and 2) because they presented outcomes as a 3‐way ANOVA making it impossible to extract the relevant data for the current meta‐analysis. Therefore, *k*_passive_ = 22 and *k*_active_ = 4 studies were analysed.

**FIGURE 3:**
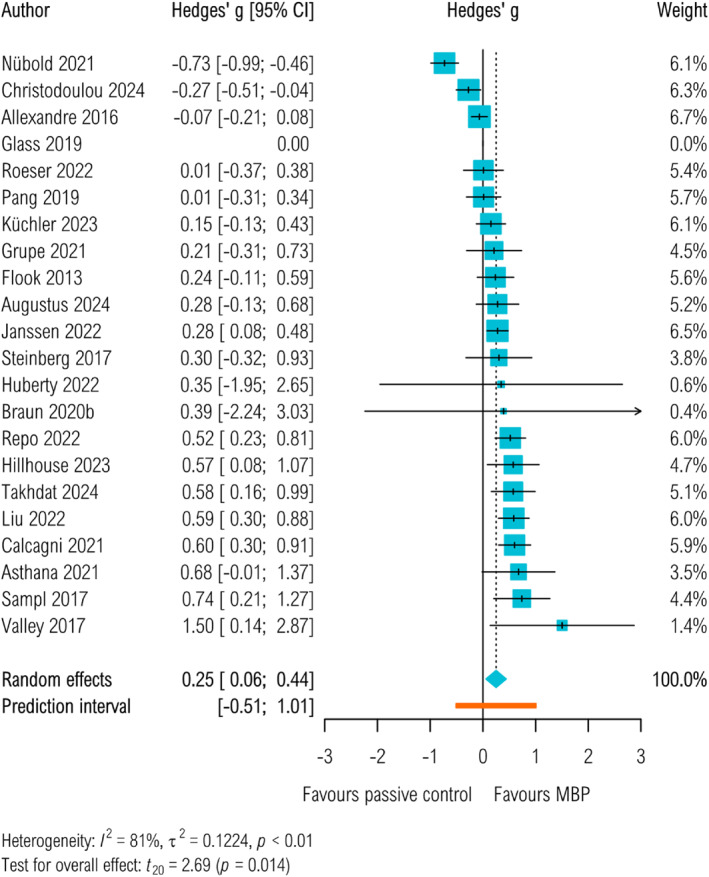
Task performance measured at post‐intervention up to 4 weeks. Passive control groups.

**FIGURE 4:**
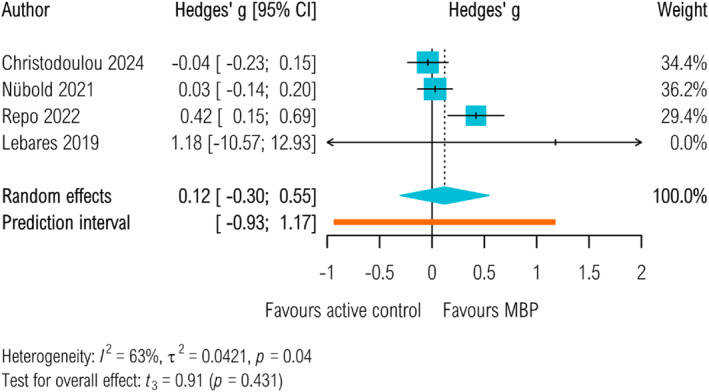
Task performance measured at post‐intervention up to 4 weeks. Active control groups.

Overall, MBP‐s were found to significantly improve task performance compared to passive control groups (Hedges' *g*_passive_ = 0.25, 95% CI 0.06 to 0.44, *p* = 0.01, 95% PI −0.51–1.01). There was high heterogeneity (τ^2^_passive_ = 0.12 (95% CI 0.05–0.28) *I* ^2^ = 81.48%.) As a result, the prediction intervals are wide (95% PI −0.51–1.01) and include zero, which indicates that there is not 95% certainty that the result will apply to every one of the settings represented by the included trials. There were insufficient studies to perform a comparison with non‐specific control groups, but MBPs were not found to significantly improve task performance compared to active control groups specifically aiming to improve work performance by other approaches (Hedges' *g*_active_ = 0.12, 95% CI −0.3 to 0.55, *p* = 0.43, 95% PI −0.93 to 1.17, τ^2^_active_ = 0.04, 95% CI 0 to 0.64, *I* ^2^ = 62.87%, see Figure 3, Figure 4 and Table S2).

Moderator analysis on the primary outcome in studies with passive control groups suggested that interventions offered to students (*k* = 8) had a larger effect size (Hedges' *g* = 0.43, 95% CI 0.18 to 0.67, *I* ^2^ = 13.13%) compared to those offered to employees (*k* = 14, Hedges' *g* = 0.16, 95% CI −0.1 to 0.42, *I* ^2^ = 84.71%), however this difference was not statistically significant (Cochran's *Q*‐test χ ^2^(1) = 2.99, *p* < 0.08). When comparing mode of delivery, face‐to‐face interventions (*k* = 18) had a larger effect size (Hedges' *g* = 0.34, 95% CI 0.17 to 0.51, *I* ^2^ = 67.03%) compared to other types (self‐paced and those involving a mix of self‐paced and face‐to‐face sessions (*k* = 4, Hedges' *g* = −0.19, 95% CI −0.98 to 0.6, *I* ^2^ = 87.75%). This difference was statistically significant (Cochran's *Q*‐test χ ^2^(1) = 4.17, *p* = 0.04). The effect sizes were not statistically different when comparing studies that used self‐reports (*k* = 14, Hedges' *g* = 0.25, 95% CI −0.03 to 0.53, *I* ^2^ = 85.22%)) with those that did not (*k* = 7, Hedges' *g* = 0.31, 95% CI −0.02 to 0.65, *I* ^2^ = 52.94%, Cochran's *Q*‐test χ ^2^(1) = 0.12, *p* = 0.73). Hours of guided content (regression coefficient = 0.02, 95% CI 0 to 0.04, *I* ^2^ = 79.06%, *p* = 0.08, see Table S11) had no significant effect on task performance.

We did not run subgroup analyses to explore effects against distinct types of active control groups as there were too few studies. We could not run the pre‐registered sensitivity analysis where we planned to exclude studies with high risk of bias as all studies included in the primary outcome analysis were rated as having high risk of bias.

### 3.5 Secondary Outcomes

Secondary outcomes included other time periods for the primary outcome of interest and other ways of operationalising individual performance at different time periods. Table 2 summarises the findings of the meta‐analyses (forest plots in Supplementary Material 3, Figures S3‐S14).

**TABLE 2:** Secondary outcome meta‐analyses’ results.

Domain	Time	Control	k	*g* ^a^ ^,^ ^b^	*I* ^2^	95% CI	Pred. int^c^
Task performance	5–24 w	Passive	13	0.03	67.82%	−0.1 to 0.15	−0.35 to 0.4
	> 24w	Passive	3	0.08	0%	−0.27 to 0.43	−0.96 to 1.11
Contextual performance	< 4w	Passive	18	0.4**	96.23%	0.18 to 0.63	−0.44 to 1.25
		Placebo	3	0.19	72.83%	−0.95 to 1.33	−5.76 to 6.15
	5–24 w	Passive	10	0.44*	90.02%	0.13 to 0.75	−0.51 to 1.39
		Active intervention	4	0.04	58.36%	−0.35 to 0.42	−0.8 to 0.88
Adaptive performance	< 4w	Passive	24	0.19**	67.52%	0.08 to 0.3	−0.22 to 0.6
		Placebo	6	0.02	51.76%	−0.21 to 0.24	−0.43 to 0.46
		Active intervention	3	0.16*	0%	0.07 to 0.26	−0.11 to 0.43
	5–24 w	Passive	15	0.28*	70.06%	0.03 to 0.53	−0.5 to 1.07
Counterproductive work behaviour	< 4w	Passive	8	0.25	96.06%	−0.23 to 0.72	−1.08 to 1.57

^a^ Hedges' *g*.

^b^ *p < 0.05, **p < 0.01.

^c^ Prediction interval.

Across the domains, the minimum Hedges' *g* value was 0.03 and the highest 0.44. Some effect sizes were statistically significant but the possibility of spurious results due to multiple testing needs to be considered. Heterogeneity is high in most cases and prediction intervals below zero suggest negative effects cannot be ruled out in any of the domains.

#### 3.5.1 Reporting Biases and Confidence in the Evidence

We investigated selective under‐reporting or non‐reporting of results. While the funnel plot (see Supplementary Materials 3 Figure S1 and S2) revealed no clear publication bias, we found 41 potentially eligible trials publicly registered over three years ago that had not made their results available (see Supplementary Material 3 for details). Second, of the studies included in this systematic review (*k* = 99), we had to exclude 20 studies across the different analyses, because of lack of reports of collected data (see Supplementary Materials for details).

#### 3.5.2 Certainty of Evidence

The overall certainty of evidence assessed with GRADE is very low. The main drivers for low confidence were high risk of bias, high non‐reporting bias, imprecision, and inconsistency (see Supplementary Materials 3, Table S1).

## 4 Discussion

This systematic review and meta‐analysis synthesised evidence from RCTs which evaluated the impact of MBPs on work performance. It makes several novel contributions to the literature. First, it is the first meta‐analysis to categorise work performance into distinct, theory‐driven domains, enabling a more granular understanding of MBPs' effects across different facets of performance. Second, unlike previous reviews that grouped together a broad array of contemplative practices, this review applies a widely accepted, consensus‐based definition of MBPs (National Institute for Health and Clinical Excellence 2022), thereby improving conceptual clarity and ensuring alignment with established standards in the field. Third, it uniquely compares MBPs against a range of control conditions—including passive controls, placebo (e.g., attention) controls, and active work performance‐enhancing interventions—allowing for a more precise estimation of their specific comparative effects. Finally, it includes studies from a wide range of settings, including occupational, educational, and community contexts, enhancing the generalisability of the findings.

Our primary analysis indicated that offering MBPs improves task performance (i.e., the quality and quantity of work) up to at least a month after the intervention, with our secondary time point indicating that these effects may last for longer. When considering other aspects of work performance using our secondary outcomes, we observed small effect sizes in favour of MBPs on improving adaptive and contextual performance. However, no statistically significant effects were found on counterproductive work behaviour. This may be due to the number of studies assessing counterproductive work behaviour being very low, limiting statistical power and increasing the influence of heterogeneity in measurement and context. Additionally, the lack of effect could be partly due to how this construct was operationalised in the included studies—primarily as absenteeism or presenteeism. As MBPs are often designed as preventative interventions, they may not be sufficient for individuals already experiencing substantial distress, such as those requiring sick leave or attending work despite poor health. These individuals may benefit more from targeted clinical or organisational interventions. Together, these factors could have contributed to the absence of statistically significant findings in this domain. Comparison against GRADE criteria indicates that the quality of the current evidence is very low due to high risk of bias within the included trials, and heterogeneity in the results across different delivery settings.

Furthermore, when comparing MBPs to other active interventions implemented to improve task performance, we found no evidence that MBPs outperformed alternative programmes within 4 weeks post‐intervention. These results replicate findings in the general population, where MBPs did not outperform active control groups targeting mental health outcomes (Galante et al. 2021). Similarly, in children and adolescents, MBPs have been shown to reduce anxiety symptoms but not other mental health or executive function outcomes when compared with active control groups (Dunning et al. 2022). Given the comparison included non‐specific active control groups, the observed effects may overestimate MBPs' true performance relative to alternative interventions. As the investigation into the mechanisms of MBPs is still in its infancy, there is little evidence to suggest it is inherently impossible for MBPs to outperform alternative interventions. MBPs relative effectiveness may also depend on the definition of work performance. For example, MBPs showed a slightly greater improvement in adaptive performance compared to other work performance domains at post‐intervention, but this conclusion is based on three studies with prediction intervals suggesting a possible a negative effect and effect sizes so small that real‐world significance is questionable. Overall, this meta‐analysis suggests that there is currently little evidence that MBPs outperform other interventions commonly offered by organisations (e.g., time/stress management seminars, gym passes, free fruit) or organisational changes, such as improving workload and working conditions.

Heterogeneity in our results suggests that the effects of MBPs may be context‐specific. For example, our subgroup analyses indicated that the beneficial effects of MBPs (relative to passive control groups) were larger when the MBP was delivered in face‐to‐face format compared to other forms of delivery, such as app‐based interventions. Similarly, high heterogeneity may suggest that MBPs might be useful for improving work performance in some occupational settings (e.g., for desk‐based roles) but not others, although we lacked data to test this hypothesis. Determining the optimal conditions for MBP delivery requires careful study design, particularly since certain relevant variables, like organisational culture, may be difficult to measure. Indeed, there is some evidence that, where conflicts of interest, and issues with relative power are prevalent, MBPs may actually have a detrimental effect on prosocial behaviour (Donald et al. 2019; Columbus et al. 2022; Poulin et al. 2021).

Our review also highlights a lack of emphasis on disentangling MBPs' specific effects on work performance from the general mental health benefits that MBPs may yield. If improvements in work performance occur primarily—or perhaps exclusively—through an indirect pathway via mental health [for example, Asuero et al. 2014], it may be more practical for organisations to offer a range of approaches to supporting well‐being at work (National Institute for Health and Clinical Excellence 2022). Providing a range of programmes would accommodate employees' and students' preferences and needs (e.g., opportunities for physical exercise) and increase the probability of a programme uptake.

### 4.1 Implications for Future Research

A rigorous evaluation of the effects of mindfulness‐based programmes (MBPs) on real‐world outcomes is essential to ensure responsible and effective implementation. First, there is a strong need for improving open‐science practices. We found 41 potentially eligible studies that had not published their results at least 3 years after initial trial registration. Where results were published, all studies, except one, were rated to have a high overall risk of bias. Some factors contributing to risk of bias are difficult to avoid, for example blinding participants or facilitators of behavioural programmes. However, upgrading randomisation and allocation concealment processes and pre‐registration of outcomes and planned analyses are free, simple, and much‐needed steps. Second, tightening the operationalisation of work performance in MBP efficacy/effectiveness research will also benefit the field. In this meta‐analysis, 91 different instruments were used to index work performance across four domains, diluting interpretability and impacting estimates. Similarly, further use of objective (e.g., blinded raters) rather than subjective measures of work performance is needed.

Future trials of MBPs should investigate underlying mechanisms of action, as evidence so far suggests they may work through self‐regulatory processes (Glomb et al. 2011), increased motivation (Hafenbrack et al. 2022) or improved task engagement (Cheung et al. 2020). Although our review did not directly examine mechanisms, exploring distinct domains of work performance can help identify the specific skills and behaviours most likely influenced by MBPs, thereby enabling more targeted evaluations and supporting the refinement of interventions. Our finding suggest that task performance and adaptive work performance could yield similar effect sizes when MBPs were compared with other work performance‐enhancing interventions, although due to multiple testing, these findings need to be replicated.

To advance understanding of the theoretical underpinnings and mechanisms of action of MBPs, more research is needed to establish their effectiveness relative to alternative interventions. Such studies are critical for guiding employers' procurement decisions and shaping governmental guidelines. Both lines of investigation require trials with active control groups; yet powering these trials remains challenging due to the absence of meta‐analytic effect size estimates for MBPs compared with active controls.

### 4.2 Implications for Policy and Practice

The current evidence base is insufficiently robust to support clear implications for policy and practice. While there is some indication that MBPs may improve work performance, prediction intervals suggest MBPs could also negatively affect any of the four domains of work performance. Moreover, little is known about how MBPs compare with other individual‐level interventions aimed to improve well‐being or work performance. This uncertainty raises concerns about high participant burden and organisational cost for minimal —or even potential declines—in performance. Additionally, possible adverse effects, such as psychological distress or physical discomfort, have been documented for some individuals (Hafenbrack et al. 2022; Baer et al. 2021; Cebolla et al. 2017).

Given the limited confidence that MBPs outperform alternative interventions, employers are to continue offering a range of evidence‐based options to accommodate diverse employee preferences and needs. Organisations should remain vigilant regarding potential negative effects of any intervention, including MBPs. Therefore in addition to introducing well‐being programmes, organisations need to implement systems to monitor and respond to reports of deteriorating well‐being, work performance, or perceived ability to work, thereby ensuring that appropriate support is available for individuals who do not benefit—or may be harmed—by such programmes.

### 4.3 Limitations

Our meta‐analysis highlights several factors that warrant caution in interpreting the findings. First, we observed substantial heterogeneity between the studies. This could be due to the operationalisation of work performance or variation in study settings, as suggested by the subgroup analyses indicating high heterogeneity among employee‐focused trials. Second, we excluded grey literature, a decision that may have amplified publication bias. Third, by applying a specific definition of work performance, we excluded some job‐related outcomes (job satisfaction, work motivation, job‐related stress and burnout) that did not align with the Koopmans and colleagues' (2011) model of work performance. Consequently, this meta‐analysis does not capture all aspects of work and study‐related experiences.

Moreover, adherence to the existing framework required categorising outcomes in ways that others might interpret differently. For example, absenteeism could be viewed as an adaptive response to poor health, where taking sick leave facilitates recovery and mitigates complications. We chose not to modify the framework to maintain consistency and standardisation across the available evidence. However, researchers adopting alternative conceptualisations are encouraged to use our publicly available dataset, which allows outcomes to be reclassified according to their preferences.

Additionally, the large number of outcome measures used to capture work performance may have contributed to heterogeneity and limits this review's ability to advance theory. As noted earlier, there is a need to better understand the mechanisms through which MBPs might influence work performance—a task so far complicated by the lack of an overview of how work performance has been conceptualised in MBP research and the absence of a clear mapping of potential domains.

Finally, we did not adjust our analyses for multiple comparisons, meaning the false positive rate is controlled only for the primary outcome. Therefore, results for secondary outcomes should be interpreted with caution, consistent with standard practice.

## 5 Conclusions

To our knowledge, this synthesis represents the first systematic effort to map how work performance is measured in MBP research. The findings can inform researchers' decisions regarding outcome selection, thereby enhancing both construct as well as ecological validity. Addressing the existing research limitations and comparing MBPs with active control groups are important next steps for guiding the effective and responsible implementation of MBPs in professional and academic settings.

## Author Contributions

Conceptualisation, methodology: MV, CH, JG, data curation: MV, TBO, AK, NK, FM, CMS, AS, SS, TW, MB, formal analysis, project administration, software, writing – original draft: MV, validation: CH, JG, resources, supervision: CH, JG, TD. All authors contributed to reviewing and editing.

## Funding

This study was supported conduced at the MRC Cognition and Brain Sciences Unit at the University of Cambridge. MV was supported by a Kristjan Jaak degree studies abroad scholarship from Estonia's Education and Youth Board and the Estonian Research Council (PRG2190). JG was supported by the National Institute for Health and Care Research (NIHR) Applied Research Collaboration East of England (NIHR ARC EoE) at the Cambridge and Peterborough NHS Foundation Trust, and by the Contemplative Studies Centre, established by a philanthropic gift from the Three Springs Foundation, Pty. Ltd at the University of Melbourne. The views expressed are those of the author and not necessarily those of the NIHR or the Department of Health and Social Care. CH was supported by the Economic and Social Research Council (ES/R010781/1) and Australian Research Council (DE20010004) and TD by the UK Medical Research Council (Grant reference: SUAG/043 G101400) and Wellcome Trust (Grant reference: 104908/Z/14/Z, 107496/Z/15/Z).

## Conflicts of Interest

The authors declare no conflicts of interest.

## Supporting information


Supporting Information S1

## Data Availability

The data collected and the code to analyse this meta‐analysis are available on OSF: https://osf.io/vwu24/. This study was pre‐registered at PROSPERO (#191756).
