# Supplementary material for: Mindfulness‐Based Programmes for Work Performance: A Systematic Review and Meta‐Analysis of Randomised Controlled Trials
Source: Stress Health. 2025 Dec 5;41(6):e70123. doi: 10.1002/smi.70123 (PMC12680914; doi:10.1002/smi.70123)
Supplement: Supplementary file 1 — Supporting Information S1 [file SMI-41-e70123-s001.pdf]

The effectiveness of mindfulness on work performance: A systematic review and meta-analysis of randomised controlled trials

Supplementary information

Maris Vainre[1,2,3], Tim Dalgleish[1,4], Tia Bendriss-Otiko[*5], Molly Butler[*3], Amelia Kirkpatrick[*3], Nana Kosugiyama[*6], Fabiana Mariscotti[*7], Candelaria Martinez-Sosa[*5], Athina Sideri[*8], Sebastian Sönksen[*6], Tim Wood[*6], Caitlin Hitchcock[**1,3], Julieta Galante[**6,7]

[1]Medical Research Council Cognition and Brain Sciences Unit, University of Cambridge, UK; [2]Institute of Psychology, University of Tartu, Estonia; [3]School of Psychological Sciences, University of Melbourne, Melbourne, Victoria, Australia; [4]Cambridgeshire and Peterborough NHS Foundation Trust, Cambridgeshire and Peterborough, UK; [5]North East London NHS Foundation Trust, UK; [6]Contemplative Science Centre, University of Melbourne, Melbourne, Victoria, Australia; [7]Department of Psychiatry, University of Cambridge, UK; [8]Norfolk and Suffolk NHS Foundation Trust, UK;

*Contributed equally, listed alphabetically
**Joint senior authors

For the purpose of open access, the author has applied a Creative Commons Attribution (CC BY) licence to any Author Accepted Manuscript version arising from this submission.

Table of contents

SUPPLEMENTARY MATERIALS 1: SEARCH TERMS **3**
- ASSIA 3
- Cochrane Central Register of Controlled Trials (CENTRAL) 6
- Embase 6
- ERIC 8
- ICTRP 10
- Medline and Pubmed 10
- PsycInfo 12
- Scopus 15
- Web of Science 16

SUPPLEMENTARY MATERIAL 2: DATA EXTRACTION FORMS **17**
- Data extraction items 17
- Risk of bias evaluation 18

SUPPLEMENTARY MATERIAL 3: METHOD AND RESULTS **22**

Confidence in the evidence 22
Risk of bias rating for each study 24
Reporting bias 51

OUTCOME MEASURES USED 54

Studies not meta-analysed due to lack of reports on outcomes 71
Summary statistics for each study 73
Main outcome 81
Secondary outcome analyses 85

REFERENCES 97

Supplementary Materials 1: Search terms

ASSIA

Date of search

02/08/2024

Number of records found

1142

Search strategy

((employ* OR job* OR labo* OR occupation* OR personn* OR staff OR unemploy* OR work*) OR (MAINSUBJECT.EXACT.EXPLODE("Academic staff") OR MAINSUBJECT.EXACT.EXPLODE("Academic work") OR MAINSUBJECT.EXACT.EXPLODE("Employability") OR MAINSUBJECT.EXACT.EXPLODE("Employee participation") OR MAINSUBJECT.EXACT.EXPLODE("Employees") OR MAINSUBJECT.EXACT.EXPLODE("Employers") OR MAINSUBJECT.EXACT.EXPLODE("Employment based education") OR MAINSUBJECT.EXACT.EXPLODE("Employment") OR MAINSUBJECT.EXACT.EXPLODE("Group work") OR MAINSUBJECT.EXACT.EXPLODE("Labor") OR MAINSUBJECT.EXACT.EXPLODE("Labor force") OR MAINSUBJECT.EXACT.EXPLODE("Personnel management") OR MAINSUBJECT.EXACT.EXPLODE("Psychiatric staff nurses") OR MAINSUBJECT.EXACT.EXPLODE("Teamwork") OR MAINSUBJECT.EXACT.EXPLODE("Security staff") OR MAINSUBJECT.EXACT.EXPLODE("Staff cafeteria") OR MAINSUBJECT.EXACT.EXPLODE("Staff development") OR MAINSUBJECT.EXACT.EXPLODE("Staff grade") OR MAINSUBJECT.EXACT.EXPLODE("Staff nurses") OR MAINSUBJECT.EXACT.EXPLODE("Staff") OR MAINSUBJECT.EXACT.EXPLODE("Staffing") OR MAINSUBJECT.EXACT.EXPLODE("Unemployed") OR MAINSUBJECT.EXACT.EXPLODE("Unemployment") OR MAINSUBJECT.EXACT.EXPLODE("Work") OR MAINSUBJECT.EXACT.EXPLODE("Workplaces")) OR (academi* OR college* OR educat* OR HEI OR student* OR university) OR (higher NEAR/1 education) AND (MAINSUBJECT.EXACT.EXPLODE("Colleges & universities") OR MAINSUBJECT.EXACT("Academic staff") OR MAINSUBJECT.EXACT.EXPLODE("Academic work") OR MAINSUBJECT.EXACT.EXPLODE("Adult education") OR MAINSUBJECT.EXACT.EXPLODE("Community colleges") OR MAINSUBJECT.EXACT.EXPLODE("Graduate studies")

MAINSUBJECT.EXACT.EXPLODE("Education") OR MAINSUBJECT.EXACT.EXPLODE("Continuing education") OR MAINSUBJECT.EXACT.EXPLODE("Higher education") OR MAINSUBJECT.EXACT.EXPLODE("Postdoctoral education") OR MAINSUBJECT.EXACT.EXPLODE("Student health services") MAINSUBJECT.EXACT.EXPLODE("Students") OR MAINSUBJECT.EXACT.EXPLODE("Undergraduate students"))) AND ((ab(mindful* OR meditat* OR MBCT OR MBSR) OR ti(mindful* OR meditat* OR MBCT OR MBSR)) OR MAINSUBJECT.EXACT.EXPLODE("Meditation")) AND ((absent* OR achiev* OR adher* OR attainm* OR attend* OR burnout OR conduct* OR disengage* OR distress* OR effective* OR effic* OR engagem* OR error* OR function* OR mistak* OR motivate* OR output* OR perform* OR present* OR product* OR procrast* OR stress* OR underperform*) OR (job NEAR/2 strain) AND (MAINSUBJECT.EXACT("Fitness for work") OR MAINSUBJECT.EXACT("Low achievers") OR MAINSUBJECT.EXACT.EXPLODE("Absenteeism") OR MAINSUBJECT.EXACT.EXPLODE("Academic achievement") OR MAINSUBJECT.EXACT.EXPLODE("Academic climate") OR MAINSUBJECT.EXACT.EXPLODE("Academic misconduct") OR MAINSUBJECT.EXACT.EXPLODE("Achievement") OR MAINSUBJECT.EXACT.EXPLODE("Adherence") OR MAINSUBJECT.EXACT.EXPLODE("Athletic performance") OR MAINSUBJECT.EXACT.EXPLODE("Attendance") MAINSUBJECT.EXACT.EXPLODE("Behavior") MAINSUBJECT.EXACT.EXPLODE("Burnout") OR MAINSUBJECT.EXACT.EXPLODE("Cognitive performance") OR MAINSUBJECT.EXACT.EXPLODE("Cognitive-Motor performance") OR MAINSUBJECT.EXACT.EXPLODE("Cost analysis") OR MAINSUBJECT.EXACT.EXPLODE("Labor productivity") OR MAINSUBJECT.EXACT.EXPLODE("Productivity") OR MAINSUBJECT.EXACT.EXPLODE("Disengagement") OR MAINSUBJECT.EXACT.EXPLODE("Effectiveness") OR MAINSUBJECT.EXACT.EXPLODE("Efficacy") OR MAINSUBJECT.EXACT.EXPLODE("Emotional distress") OR MAINSUBJECT.EXACT.EXPLODE("Work skills") OR MAINSUBJECT.EXACT.EXPLODE("Engagement") OR MAINSUBJECT.EXACT.EXPLODE("Ethical conduct") OR MAINSUBJECT.EXACT.EXPLODE("Extrinsic motivation") OR MAINSUBJECT.EXACT.EXPLODE("Functional performance") OR MAINSUBJECT.EXACT.EXPLODE("Group dynamics") OR MAINSUBJECT.EXACT.EXPLODE("Group performance") OR MAINSUBJECT.EXACT.EXPLODE("Intrinsic motivation") OR MAINSUBJECT.EXACT.EXPLODE("Competence") MAINSUBJECT.EXACT.EXPLODE("Job content") OR MAINSUBJECT.EXACT.EXPLODE("Job control") OR MAINSUBJECT.EXACT.EXPLODE("Job evaluation") OR MAINSUBJECT.EXACT.EXPLODE("Job experiences") OR MAINSUBJECT.EXACT.EXPLODE("Job leaving") OR MAINSUBJECT.EXACT.EXPLODE("Job performance") OR MAINSUBJECT.EXACT.EXPLODE("Job satisfaction") OR MAINSUBJECT.EXACT.EXPLODE("Misconduct") OR MAINSUBJECT.EXACT.EXPLODE("Professional misconduct") OR

MAINSUBJECT.EXACT.EXPLODE("Academic misconduct") OR MAINSUBJECT.EXACT.EXPLODE("Errors") OR MAINSUBJECT.EXACT.EXPLODE("Motivation") OR MAINSUBJECT.EXACT.EXPLODE("Motor performance") OR MAINSUBJECT.EXACT.EXPLODE("Multiple task performance") OR MAINSUBJECT.EXACT.EXPLODE("Occupational balance") OR MAINSUBJECT.EXACT.EXPLODE("Occupational commitment") OR MAINSUBJECT.EXACT.EXPLODE("Occupational culture") OR MAINSUBJECT.EXACT.EXPLODE("Occupational health and safety") OR MAINSUBJECT.EXACT.EXPLODE("Occupational health") OR MAINSUBJECT.EXACT.EXPLODE("Professional identity") OR MAINSUBJECT.EXACT.EXPLODE("Occupational prestige") OR MAINSUBJECT.EXACT.EXPLODE("Occupational stress management") OR MAINSUBJECT.EXACT.EXPLODE("Occupational stress") OR MAINSUBJECT.EXACT.EXPLODE("Organizational effectiveness") OR MAINSUBJECT.EXACT.EXPLODE("Organizational performance") OR MAINSUBJECT.EXACT.EXPLODE("Overachievement") OR MAINSUBJECT.EXACT.EXPLODE("Perceptual performance") OR MAINSUBJECT.EXACT.EXPLODE("Performance appraisal") OR MAINSUBJECT.EXACT.EXPLODE("Performance management") OR MAINSUBJECT.EXACT.EXPLODE("Performance measurement") OR MAINSUBJECT.EXACT.EXPLODE("Performance") OR MAINSUBJECT.EXACT.EXPLODE("Self-efficacy") OR MAINSUBJECT.EXACT.EXPLODE("Procrastination") OR MAINSUBJECT.EXACT.EXPLODE("Productivity measurement") OR MAINSUBJECT.EXACT.EXPLODE("Productivity") OR MAINSUBJECT.EXACT.EXPLODE("Professional conduct") OR MAINSUBJECT.EXACT.EXPLODE("Professional misconduct") OR MAINSUBJECT.EXACT.EXPLODE("Psychological distress") OR MAINSUBJECT.EXACT.EXPLODE("Labor productivity") OR MAINSUBJECT.EXACT.EXPLODE("Productivity") OR MAINSUBJECT.EXACT.EXPLODE("Role stress") OR MAINSUBJECT.EXACT.EXPLODE("School failure") OR MAINSUBJECT.EXACT.EXPLODE("Stress management training") OR MAINSUBJECT.EXACT.EXPLODE("Stress management") OR MAINSUBJECT.EXACT.EXPLODE("Task performance") OR MAINSUBJECT.EXACT.EXPLODE("Underachievement") OR MAINSUBJECT.EXACT.EXPLODE("Employee attitude") OR MAINSUBJECT.EXACT.EXPLODE("Work-Leisure attitudes") OR MAINSUBJECT.EXACT("Work cognitions") OR MAINSUBJECT.EXACT.EXPLODE("Work commitment") OR MAINSUBJECT.EXACT.EXPLODE("Work environment") OR MAINSUBJECT.EXACT.EXPLODE("Workaholism") MAINSUBJECT.EXACT("Work-Family conflict") OR MAINSUBJECT.EXACT.EXPLODE("Working conditions") OR MAINSUBJECT.EXACT.EXPLODE("Working relationships") OR

MAINSUBJECT.EXACT.EXPLODE("Work-Leisure attitudes") OR MAINSUBJECT.EXACT.EXPLODE("Work-Leisure conflict") OR MAINSUBJECT.EXACT.EXPLODE("Workloads") OR MAINSUBJECT.EXACT.EXPLODE("Workplace control") OR MAINSUBJECT.EXACT.EXPLODE("Workplace learning"))) AND ((randomise* OR randomize* OR RCT OR "random allocation" OR "random assignment") OR MAINSUBJECT.EXACT.EXPLODE("Clinical trials"))

Cochrane Central Register of Controlled Trials (CENTRAL)

Date of search

02/08/2024

Number of records found

208

Search strategy

(employ or job* or labo* or occupation* or personn* or staff or unemploy* or work*) or (academi* or college* or educat* or HEI or student* or university) in All Text AND ab(mindful* OR meditat* OR MBCT OR MBSR) or ti(mindful* OR meditat* OR MBCT OR MBSR) or MH “Mindfulness” or MH “Meditation” in All Text AND (absen* or achiev* or adher* or attainm* or attend* or burnout or conduct* or disengage* or distress* or effective* or effic* or engagem* or error* or function* or mistak* or motivate* or output* or perform* or present* or procastin* or product* or stress* or underperform*) in All Text

Embase via Ovid

Date of search

02/08/2024

Number of records found

3586

Search strategy

((employ*.mp. or job*.mp. or labo*.mp. or occupation*.mp. or personn*.mp. or staff.mp. or unemploy*.mp. or work*.mp.) or (exp administrative personnel/ or exp clinical laboratory personnel/ or exp construction work/ or exp dental personnel/ or exp dental staff/ or exp field work/ or exp health care personnel management/ or exp health care personnel/ or exp hospital personnel management/ or exp hospital personnel/ or exp job accommodation/ or

exp laboratory personnel/ or exp medical personnel/ or exp medical staff/ or exp mental health care personnel/ or exp military personnel/ or exp nursing home personnel/ or exp nursing staff/ or exp occupational health/ or exp operating room personnel/ or exp paramedical personnel/ or exp personnel management/ or exp personnel shortage/ or exp personnel/ or exp religious personnel/ or exp rescue personnel/ or exp rescue work/ or exp return to work/ or exp shift work/ or exp social work student/ or exp staff/ or staff nurse/ or exp staff training/ or exp telecommuting/ or exp unemployment/ or exp work resumption/ or exp work schedule/ or exp work/ or exp working time/ or exp workplace/) or (academi*.mp. or college*.mp. or educat*.mp. or HEI.mp. or student*.mp. or university.mp.) or (higher adj1 education).mp. or (exp adult education/ or allied health student/ or athletic training student/ or audiology student/ or baccalaureate nursing student/ or chiropractic student/ or exp clinical education/ or exp college student/ or exp college/ or exp community college/ or exp continuing education/ or dental hygiene student/ or dental student/ or dietetics student/ or disabled student/ or exp doctoral education/ or exp education program/ or education/ or foreign student/ or graduate nursing student/ or graduate student/ or health student/ or exp masters education/ or medical student/ or midwifery student/ or non-medical student/ or nontraditional student/ or nursing student/ or occupational therapy student/ or paramedical student/ or pharmacy student/ or PhD student/ or physical therapy student/ or physician assistant student/ or exp postdoctoral education/ or exp postgraduate education/ or postgraduate student/ or premedical student/ or professional student relation/ or public health student/ or research student/ or respiratory therapy student/ or social work student/ or student assistance program/ or student athlete/ or exp student retention/ or student satisfaction/ or student/ or undergraduate student/ or exp university student/ or exp university/ or veterinary student/)) AND ((Mindful*.ti,ab. or Meditat*.ti,ab. or mbct.ti,ab. or mbsr.ti,ab.) or (exp focused attention meditation/ or exp meditation/ or exp mindfulness/ or exp mindfulness meditation/ or exp open monitoring meditation/ or exp transcendental meditation/)) AND ((absen*.mp or achiev*.mp or adher*.mp or attainm*.mp or attend*.mp or burnout.mp. or conduct*.mp or disengage*.mp. or distress*.mp. or effective*.mp or effic*.mp or engagem*.mp or error*.mp or function*.mp. or mistak*.mp or motivate*.mp. or output*.mp. or perform*.mp or present*.mp or procrast*.mp or product*.mp or stress*.mp or underperform*.mp) or (job adj2 strain).mp or (exp absenteeism/ or exp academic achievement/ or exp academic failure/ or exp academic success/ or exp achievement/ or exp athletic performance/ or exp behavioral stress/ or exp burnout/ or exp diagnostic error/ or exp distress syndrome/ or exp error/ or exp health personnel attitude/ or exp job experience/ or exp job performance/ or exp job satisfaction/ or exp job security/ or exp job stress/ or exp Maslach Burnout Inventory/ or exp Maslach Burnout Inventory-General Survey/ or exp Maslach Burnout Inventory-Human Services Survey/ or exp Maslach Burnout Inventory-Student Survey/ or exp medical error/ or exp medication error/ or exp mental capacity/ or exp motivation/ or exp performance/ or exp presenteeism/ or procrastination/ or exp productivity/ or exp professional burnout/ or exp "quality of working life"/ or stress/ or student burnout/ or exp task performance/ or exp work

capacity/ or exp work engagement/ or exp work environment/ or exp work experience/ or exp work-life balance/ or exp workload/)) AND ((Clinical Trial/ or Randomized Controlled Trial/ or controlled clinical trial/ or multicenter study/ or Phase 3 clinical trial/ or Phase 4 clinical trial/ or exp RANDOMIZATION/ or Single Blind Procedure/ or Double Blind Procedure/ Or Crossover Procedure/ or PLACEBO/ or randomi?ed controlled trial$.tw. or rct.tw. or (random$ adj2 allocat$).tw. or single blind$.tw. or double blind$.tw or ((treble or triple) adj blind$).tw. or placebo$.tw. or Prospective Study) NOT (Case Study/ or case report.tw. or abstract report/ or letter/ or Conference proceeding.pt. Or Conference abstract.pt. or Editorial.pt. or Letter.pt. or Note.pt.))

ERIC via EBSCO

Date of search

02/08/2024

Number of records found

67

Search strategy

((TX employ* or TX job* or TX labo* or TX occupation* or TX personn* or TX staff or TX unemploy* or TX work*) or (TX academi* or TX college* or TX educat* or TX HEI or TX student* or TX university) or (TX (higher N1 education) or (DE "College Environment" OR DE "College Students" OR DE "College Freshmen" OR DE "College Seniors" OR DE "College Transfer Students" OR DE "First Generation College Students" OR DE "Graduate Students" OR DE "In State Students" OR DE "On Campus Students" OR DE "Out of State Students" OR DE "Preservice Teachers" OR DE "Two Year College Students" OR DE "Undergraduate Students" OR DE "Colleges" OR DE "Agricultural Colleges" OR DE "Black Colleges" OR DE "Business Schools" OR DE "Church Related Colleges" OR DE "Cluster Colleges" OR DE "Commuter Colleges" OR DE "Dental Schools" OR DE "Developing Institutions" OR DE "Experimental Colleges" OR DE "Law Schools" OR DE "Library Schools" OR DE "Medical Schools" OR DE "Multicampus Colleges" OR DE "Noncampus Colleges" OR DE "Private Colleges" OR DE "Public Colleges" OR DE "Single Sex Colleges" OR DE "Small Colleges" OR DE "Two Year Colleges" OR DE "Universities" OR DE "Upper Division Colleges" OR DE "Education" OR DE "Academic Education" OR DE "Adult Education" OR DE "Aerospace Education" OR DE "Aesthetic Education" OR DE "African American Education" OR DE "After School Education" OR DE "Aging Education" OR DE "Agricultural Education" OR DE "Alcohol Education" OR DE "Allied Health Occupations Education" OR DE "American Indian Education" OR DE "Art Education" OR DE "Basic Business Education" OR DE "Bilingual Education" OR DE "Career Education" OR DE "Coeducation" OR DE "Community Education" OR DE "Comparative

Education" OR DE "Compensatory Education" OR DE "Competency Based Education" OR DE "Compulsory Education" OR DE "Corporate Education" OR DE "Correctional Education" OR DE "Cultural Education" OR DE "Culturally Relevant Education" OR DE "Dance Education" OR DE "Distance Education" OR DE "Driver Education" OR DE "Drug Education" OR DE "Economics Education" OR DE "Energy Education" OR DE "Environmental Education" OR DE "Equal Education" OR DE "Extension Education" OR DE "Family Life Education" OR DE "General Education" OR DE "Global Education" OR DE "Health Education" OR DE "Humanistic Education" OR DE "Industrial Education" OR DE "Informal Education" OR DE "Inservice Education" OR DE "Intergroup Education" OR DE "Journalism Education" OR DE "Law Related Education" OR DE "Leisure Education" OR DE "Literacy Education" OR DE "Marine Education" OR DE "Mathematics Education" OR DE "Mexican American Education" OR DE "Migrant Education" OR DE "Music Education" OR DE "Noncategorical Education" OR DE "Nondiscriminatory Education" OR DE "Nonformal Education" OR DE "Nontraditional Education" OR DE "Open Education" OR DE "Outcome Based Education" OR DE "Outdoor Education" OR DE "Physical Education" OR DE "Place Based Education" OR DE "Police Education" OR DE "Popular Education" OR DE "Population Education" OR DE "Postsecondary Education" OR DE "Private Education" OR DE "Process Education" OR DE "Professional Education" OR DE "Progressive Education" OR DE "Public Affairs Education" OR DE "Public Education" OR DE "Religious Education" OR DE "Rural Education" OR DE "Safety Education" OR DE "Science Education" OR DE "Special Education" OR DE "STEM Education" OR DE "Study Abroad" OR DE "Supplementary Education" OR DE "Technology Education" OR DE "Tribally Controlled Education" OR DE "Urban Education" OR DE "Values Education" OR DE "Vocational Education" OR DE "Womens Education" OR DE "Education Courses"))) AND ((AB Meditat* OR TI Meditat* OR KW Meditat*or AB Mindful* or KW Mindful* or TI Mindful* or AB mbct or KW mbct or TI mbct or AB mbsr or KW mbsr or TI mbsr)) AND ((TX absen* or TX achiev* or TX adher* or TX attainm* or TX attend* or TX burnout or TX conduct* or TX disengage* or TX distress* or TX effective* or TX effic* or TX engagem* or TX error* or TX function* or TX mistak* or TX motivat* or TX output* or TX perform* or TX present* or TX procrastin* or TX product* or TX stress* or TX underperform*) or (TX (job N2 strain)) or (DE "Academic Ability" OR DE "Academic Failure" OR DE "Achievement" OR DE "Academic Achievement" OR DE "African American Achievement" OR DE "Graduation" OR DE "High Achievement" OR DE "Knowledge Level" OR DE "Low Achievement" OR DE "Overachievement" OR DE "Underachievement" OR DE "Attendance" OR DE "Average Daily Attendance" OR DE "College Attendance" OR DE "Teacher Attendance" OR DE "Attention Control" OR DE "Burnout" OR DE "Teacher Burnout" OR DE "Behavior" OR DE "Competition" OR DE "Cooperation" OR DE "Group Behavior" OR DE "Health Behavior" OR DE "Leadership Styles" OR DE "Participation" OR DE "Performance" OR DE "Persistence" OR DE "Self Control" OR DE "Student Behavior" OR DE "Teacher Behavior" OR DE "College Attendance" OR DE "Educational Attainment" OR DE "Employee Absenteeism" OR DE "Employment Problems" OR DE "Efficiency" OR DE "Teacher Effectiveness" OR DE "Teacher Improvement" OR DE "Job Performance" OR DE "Job Satisfaction" OR DE "Motivation" OR DE "Achievement Need" OR

DE "Learning Motivation" OR DE "Reading Motivation" OR DE "Self Motivation" OR DE "Student Motivation" OR DE "Teacher Motivation" OR DE "Performance" OR DE "Counselor Performance" OR DE "Failure" OR DE "Success" OR DE "Productivity" OR DE "Student Attrition" OR DE "Stress Management" OR DE "Mathematics Anxiety" OR DE "Test Anxiety" OR DE "Work Attitudes" OR DE "Job Satisfaction" OR DE "Work Environment" OR DE "Teaching Conditions") or (TX allocat* random* OR (MH "Quantitative Studies") OR (MH "Placebos") OR TX placebo* OR TX random* allocat* OR (MH "Random Assignment") OR TX randomi* control* trial* OR TX ((singl* n1 blind*) or (singl* n1 mask*)) or TX ((doubl* n1 blind*) or (doubl* n1 mask*)) or TX ((tripl* n1 blind*) or (tripl* n1 mask*)) or TX ((trebl* n1 blind*) or (trebl* n1 mask*)) OR TX clinic* n1 trial* OR DE "Randomized Controlled Trials" OR PT Clinical trial OR (MH "Clinical Trials+") or DE "Randomized Clinical Trials" OR DE "Clinical Trials" OR DE "Randomized Controlled Trials"))

ICTRP

Date of search

21/08/2024

Date restrictions: 1/1/1900-02/08/2021

Number of records

878

Search strategy

mindfulness NOT patient* NOT disturb* NOT disabilit* NOT disorder* NOT infection* NOT syndrome* NOT disease* NOT abus* NOT menopaus* NOT chronic NOT injur* NOT smok* NOT asthma NOT diabet* NOT cancer NOT stroke NOT pregn* NOT dement* NOT obese NOT weight NOT psychosis NOT PTSD NOT "Multiple Sclerosis" NOT insomn*

Medline and Pubmed via Ovid

Date of search

02/08/2024

Number of records found

2533

Search strategy

#	Search terms
1	employ*.mp or unemploy*.mp or job*.mp. or labo*.mp. or occupation*.mp. or personn*.mp or staff.mp. or work*.mp or "Staff development".mp. OR telework*.mp
2	exp Employment/ or Employment, Supported/ or Unemployment/ OR exp Occupations/ or exp Occupational Groups/ or exp Health Occupations/ or exp Students, Health Occupations/ or exp Medical Staff, Hospital/ or exp Staff Development/ or exp Medical Staff/ or exp Nursing Staff, Hospital/ or exp Nursing Staff/ or exp Return to Work/ or exp Work/ or exp Teleworking/ OR exp Workplace/ OR Shift Work Schedule/
3	university.mp. or educat*.mp. or academi*.mp. or HEI.mp. or student*.mp. or college*.mp.
4	(higher adj1 education).mp.
5	Universities/ or exp Education, Medical, Continuing/ or exp Education, Medical/ or exp Education, Medical, Graduate/ or exp Education, Nursing/ or exp Academic Medical Centers/ or Student Health Services/
6	1 or 2 or 3 or 4 or 5
7	Mindful*.ti,ab. or Meditat*.ti,ab. or mbct.ti,ab. or mbsr.ti,ab.
8	Mindfulness/ or Meditation/
9	7 or 8
10	absen*.mp or achiev*.mp or adher*.mp or attainm*.mp or attend*.mp or burnout.mp. or conduct*.mp or disengage*.mp. or distress*.mp. or effective*.mp or effic*.mp or engagem*.mp or error*.mp or function*.mp. or mistak*.mp or motivate*.mp. or output*.mp. or perform*.mp or present*.mp or product*.mp or stress*.mp or underperform*.mp
11	(job adj2 strain).mp.
12	exp absenteeism/ or Academic Failure/ or exp Academic Performance/ or achievement/ or exp burnout, psychological/ or compassion fatigue/ or educational measurement/ or exp efficiency/ or exp Efficiency, Organizational/ or Guideline Adherence/ or job satisfaction/ or exp Medication Errors/ or exp Medical Errors/ or

motivation/ or exp Occupational Health/ or exp Occupational Stress/ or presenteeism/ or Procrastination/ or exp professional competence/ or Psychological Distress/ or psychosocial functioning/ or psychology, military/ or exp Sick Leave/ or exp Stress, Psychological/ or student dropouts/ or exp "task performance and analysis"/ or time management/ or underachievement/ or exp Work/ or work engagement/ or work performance/ or Work Schedule Tolerance/

13 10 or 11 or 12

14 (Randomized Controlled Trials as Topic/ or randomized controlled trial/ or Random Allocation/ or Double Blind Method/ or Single Blind Method/ or clinical trial/ or clinical trial, phase i.pt or clinical trial, phase ii.pt or clinical trial, phase iii.pt or clinical trial, phase iv.pt or controlled clinical trial.pt or randomized controlled trial.pt or multicenter study.pt or clinical trial.pt or exp Clinical Trials as topic/) or ((clinical adj trial$).tw or ((singl$ or doubl$ or treb$ or tripl$) adj (blind$3 or mask$3)).tw or PLACEBOS/ or placebo$.tw or randomly allocated.tw or (allocated adj2 random$).tw) not (case report.tw or letter/ or historical article/)

15 6 and 9 and 13 and 14

PsycInfo via EBSCOhost

Date of search

02/08/2024

Number of records found

1528

Search strategy

((TX employ* or TX job* or labo*.mp or TX occupation* or TX personn* or TX staff or TX unemploy* or TX work*) or (DE "Aerospace Personnel" OR DE "Agricultural Extension Workers" OR DE "Agricultural Workers" OR DE "Air Force Personnel" OR DE "Aircraft Pilots" OR DE "Allied Health Personnel" OR DE "Anthropologists" OR DE "Apprenticeship" OR DE "Architects" OR DE "Army Personnel" OR DE "Artists" OR DE "Astronauts" OR DE "Attendants (Institutions)" OR DE "Attorneys" OR DE "Blue Collar Workers" OR DE "Business

and Industrial Personnel" OR DE "Child Care Workers" OR DE "Clerical Personnel" OR DE "Clinical Psychologists" OR DE "Clinicians" OR DE "Coast Guard Personnel" OR DE "College Teachers" OR DE "Commissioned Officers" OR DE "Corrections Officers" OR DE "Counselors" OR DE "Dentists" OR DE "Disabled Personnel" OR DE "Domestic Service Personnel" OR DE "Educational Personnel" OR DE "Emergency Personnel" OR DE "Engineers" OR DE "Enlisted Military Personnel" OR DE "Fire Fighters" OR DE "First Responders" OR DE "Foreign Workers" OR DE "Frontline Employees" OR DE "Government Personnel" OR DE "Health Personnel Attitudes" OR DE "Health Personnel" OR DE "Home Care Personnel" OR DE "Human Capital" OR DE "Human Resource Management" OR DE "Impaired Professionals" OR DE "Industrial Psychologists" OR DE "Information Specialists" OR DE "Job Resources" OR DE "Journalists" OR DE "Judges" OR DE "Labor Union Members" OR DE "Law Enforcement Personnel" OR DE "Lay Religious Personnel" OR DE "Legal Personnel" OR DE "Management" OR DE "Management Personnel" OR DE "Management Training" OR DE "Marine Personnel" OR DE "Mathematicians" OR DE "Medical Personnel" OR DE "Mental Health Personnel" OR DE "Middle Level Managers" OR DE "Migrant Workers" OR DE "Military Attrition" OR DE "Military Deployment" OR DE "Military Duty Status" OR DE "Military Enlistment" OR DE "Military Medical Personnel" OR DE "Military Personnel" OR DE "Military Psychologists" OR DE "Military Veterans" OR DE "National Guard Personnel" OR DE "Navy Personnel" OR DE "Noise Levels (Work Areas)" OR DE "Nonprofessional Personnel" OR DE "Nurses" OR DE "Occupational Health" OR DE "Occupational Therapists" OR DE "Optometrists" OR DE "Organizations" OR DE "Paramedics" OR DE "Paraprofessional Personnel" OR DE "Parole Officers" OR DE "Personnel" OR DE "Pharmacists" OR DE "Physical Therapists" OR DE "Physicians" OR DE "Physicists" OR DE "Police Personnel" OR DE "Prison Personnel" OR DE "Probation Officers" OR DE "Professional Personnel" OR DE "Professional Role" OR DE "Psychiatric Aides" OR DE "Psychiatric Hospital Staff" OR DE "Psychiatric Nurses" OR DE "Psychiatric Social Workers" OR DE "Psychiatrists" OR DE "Psychologists" OR DE "Psychotherapists" OR DE "Public Health Service Nurses" OR DE "Quality Control" OR DE "Religious Personnel" OR DE "Rescue Workers" OR DE "Sales Personnel" OR DE "School Administrators" OR DE "School Counselors" OR DE "School Nurses" OR DE "School Psychologists" OR DE "Scientists" OR DE "Secretarial Personnel" OR DE "Self-Managing Work Teams" OR DE "Service Personnel" OR DE "Skilled Industrial Workers" OR DE "Social Workers" OR DE "Sociologists" OR DE "Speech Therapists" OR DE "Teacher Aides" OR DE "Teacher Effectiveness" OR DE "Teachers" OR DE "Technical Personnel" OR DE "Technical Service Personnel" OR DE "Telecommuting" OR DE "Therapists" OR DE "Top Level Managers" OR DE "Unemployment" OR DE "Virtual Teams" OR DE "Volunteer Military Personnel" OR DE "White Collar Workers" OR DE "Work Teams" OR DE "Working Conditions" OR DE "Working Space" OR DE "Workplace Intervention") or (TX academi* or TX college* or TX educat* or TX HEI or TX student* or TX university.mp.) or TX (higher N1 education) or (DE "Academic Settings" OR DE "College Athletes" OR DE "College Environment" OR DE "College Graduates" OR DE “college students” OR DE "Community College Students" OR DE

"Continuing Education" OR DE "Education Students" OR DE "Graduate Education" OR DE "Higher Education" OR DE "Junior College Students" OR DE "Nursing Students" OR DE "Postgraduate Training" OR DE "ROTC Students" OR DE "Schools" OR DE "Undergraduate Education")) AND (AB Meditat* OR TI Meditat* OR KW Meditat*or AB Mindful* or KW Mindful* or TI Mindful* or AB mbct or KW mbct or TI mbct or AB mbsr or KW mbsr or TI mbsr or DE "Meditation" OR DE "Mindfulness" OR DE "Mindfulness-Based Interventions") AND ((TX absen* or TX achiev* or TX adher* or TX attainm* or TX attend* or TX burnout or TX conduct* or TX disengage* or TX distress* or TX effective* or TX effic* or TX engagem* or TX error* or TX function* or TX mistak* or TX motivat* or TX output* or TX perform* or TX present* or TX procrastinat* or TX product* or TX stress* or TX underperform*.mp) OR TX (job N2 strain) or (DE "Academic Achievement Motivation" OR DE "Academic Achievement" OR DE "Academic Aptitude" OR DE "Academic Environment" OR DE "Academic Failure" OR DE "Academic Overachievement" OR DE "Academic Stress" OR DE "Academic Underachievement" OR DE "Achievement Motivation" OR DE "Achievement" OR DE "Affiliation Motivation" OR DE "Aspirations" OR DE "Athletic Performance" OR DE "Career Change" OR DE "Career Development" OR DE "Chronic Stress" OR DE "Classroom Environment" OR DE "College Academic Achievement" OR DE "Compassion Fatigue" OR DE "Coping Behavior" OR DE "Coping Style" OR DE "Costs and Cost Analysis" OR DE "Course Evaluation" OR DE "Demoralization" OR DE "Distress" OR DE "Educational Attainment Level" OR DE "Educational Incentives" OR DE "Emotional Exhaustion" OR DE "Employee Absenteeism" OR DE "Employee Attitudes" OR DE "Employee Benefits" OR DE "Employee Characteristics" OR DE "Employee Efficiency" OR DE "Employee Engagement" OR DE "Employee Layoffs" OR DE "Employee Motivation" OR DE "Employee Productivity" OR DE "Employee Skills" OR DE "Employee Turnover" OR DE "Employee Well Being" OR DE "Endurance" OR DE "Environmental Stress" OR DE "Extrinsic Motivation" OR DE "Family Work Conflict" OR DE "Family Work Relationship" OR DE "Fear of Success" OR DE "Goals" OR DE "Group Performance" OR DE "Incentives" OR DE "Intrinsic Motivation" OR DE "Job Analysis" OR DE "Job Demands" OR DE "Job Enrichment" OR DE "Job Involvement" OR DE "Job Knowledge" OR DE "Job Performance" OR DE "Job Satisfaction" OR DE "Labor Management Relations" OR DE "Life Skills" OR DE "Morale" OR DE "Motivation Measures" OR DE "Motivation" OR DE "Motor Performance" OR DE "Occupational Adjustment" OR DE "Occupational Aspirations" OR DE "Occupational Attitudes" OR DE "Occupational Interests" OR DE "Occupational Safety" OR DE "Occupational Stress" OR DE "Occupational Success Prediction" OR DE "Occupational Success" OR DE "organizational behavior" OR DE "organizational climate" OR DE "Organizational Effectiveness" OR DE "Perceived Stress" OR DE "Performance" OR DE "Personnel Evaluation" OR DE "Personnel Placement" OR DE "Personnel Promotion" OR DE "Personnel Supply" OR DE "Personnel Termination" OR DE "Personnel Training" OR DE "Personnel" OR DE "Physical Endurance" OR DE "Physical Fitness" OR DE "Procrastination" OR DE "Productivity" OR DE "Professionalism" OR DE "Psychological Endurance" OR DE "Psychological Stress" OR DE "Readiness to Change" OR DE "School Environment" OR DE "Self-Efficacy" OR DE "Self-Expansion" OR DE "Social

Functioning" OR DE "Social Motivation" OR DE "Social Stress" OR DE "Stress Management" OR DE "Stress Reactions" OR DE "Stress" OR DE "Student Attitudes" OR DE "Student Attrition" OR DE "Student Engagement" OR DE "Supervisor Employee Interaction" OR DE "Test Performance" OR DE "well being" OR DE "Work (Attitudes Toward)" OR DE "Work Related Illnesses" OR DE "Work Rest Cycles" OR DE "Work Scheduling" OR DE "Work Week Length" OR DE "Workaholism" OR DE "Workday Shifts" OR DE "Working Conditions" OR DE "Working Space" OR DE "Work-Life Balance")) AND (TX allocat* random* OR (MH "Quantitative Studies") OR (MH "Placebos") OR TX placebo* OR TX random* allocat* OR (MH "Random Assignment") OR TX randomi* control* trial* OR TX ((singl* n1 blind*) or (singl* n1 mask*)) or TX ((doubl* n1 blind*) or (doubl* n1 mask*)) or TX ((tripl* n1 blind*) or (tripl* n1 mask*)) or TX ((trebl* n1 blind*) or (trebl* n1 mask*)) OR TX clinic* n1 trial* OR PT Clinical trial OR (MH "Clinical Trials+") or DE "Randomized Clinical Trials" OR DE "Clinical Trials" OR DE "Randomized Controlled Trials")

Scopus

Date of search

02/08/2024

Number of records found

4276

Search startegy

(TITLE-ABS-KEY (employ* or job* or labo* or occupation* or personn* or staff or unemploy* or work*) or (EXACTKEYWORD , "Workplace") or TITLE-ABS-KEY (academi* or college* or educat* or HEI or student* or university) or (EXACTKEYWORD , "Education") OR (EXACTKEYWORD , "University") OR (EXACTKEYWORD , "Universities") OR TITLE-ABS-KEY (higher W/1 education)) AND (TITLE-ABS-KEY (mindful* OR meditat* OR mbct OR mbsr) OR (exactkeyword AND , "Meditation") OR (exactkeyword AND , "Mindfulness Based Stress Reduction") OR (exactkeyword AND , "Mindfulness Meditation") OR (exactkeyword AND , "Mind-Body Therapies")) AND (ALL (absen* OR achiev* OR adher* OR attainm* OR attend* OR burnout OR conduct* OR disengage* OR distress* OR effective* OR effic* OR engagem* OR error* OR function* OR mistak* OR motivate* OR output* OR perform* OR present* OR procastin* OR product* OR stress* OR underperform*) OR ALL (job W/2 strain) OR (exactkeyword AND , "Stress") OR (exactkeyword AND , "Mental Stress") OR (exactkeyword AND , "Stress, Psychological") OR (exactkeyword AND , "Stress Management") OR (exactkeyword AND , "Burnout") OR (exactkeyword AND , "Burnout, Professional") OR (exactkeyword AND , "Job Stress")) AND (TITLE-ABS-KEY (randomise* OR randomize* OR rct OR "random allocation" OR

"random assignment" OR randomly) OR (exactkeyword AND , "Randomized Controlled Trial (topic)") OR (exactkeyword AND , "Clinical Trial") OR (exactkeyword AND , "Intervention Study") OR (exactkeyword AND , "Single Blind Procedure") OR (exactkeyword AND , "Controlled Clinical Trial") OR (exactkeyword AND , "Clinical Effectiveness")) AND (EXCLUDE (EXACTKEYWORD , "Child"))

Web of Science

Date of search

02/08/2024

Number of records found

3977

Search strategy

(((TS=(employ* OR job* OR labo* OR occupation* OR personn* OR staff OR unemploy* OR work*) OR TS=(academi* OR college* OR educat* OR HEI OR student* OR university)) AND TS=((mindful* OR meditat* OR MBCT OR MBSR))) AND ALL=(absen* OR achiev* OR adher* OR attainm* OR attend* OR burnout OR conduct* OR disengage* OR distress* OR effective* OR effic* OR engagem* OR error* OR function* OR mistak* OR motivate* OR output* OR perform* OR present* OR procrastin* OR product* OR stress* OR underperform* OR job strain)) AND ALL=(randomise* OR randomize* OR RCT OR "random allocation" OR "random assignment" OR randomly)

Supplementary Material 2: Data extraction forms

Data extraction items

Covidence #
Study ID
Title
Reviewer Name
Reviewer name
Year of first publication
Country in which the study conducted
Notes
Confirm eligibility for review
Reason for exclusion
Sample context
Sample description
Total number of arms
Total number of mindfulness groups
Study design
Any comments on the method
For each MBP arm
- Name of intervention
- Duration of intervention
- Delivery medium of intervention
- Delivery format of intervention
- Any comments on intervention

For each control arm
- Name of control group
- Type of control group
- Content of control group
- Duration of control
- Delivery medium of control
- Delivery format of control
- Any comments on control

Total number of participants
Age
- Total Mean
- Total SD
- Total Min
- Total Max

Sex
- Females
- Males
- Others

For each group
- Number of participants
- Age

Mean
SD
Min
Max
Sex
Females
Males
Others
For each outcome of interest
Dimension of outcome
Construct measured
Scale or instrument used
Cronbach's alpha or other measure for quality of the measure
Report type
Time period ranges reported
Earlier than 4 weeks before intervention
Up to 4 weeks before intervention
Up to 4 weeks after intervention
5 - 24 weeks after intervention
More than 24 weeks post-intervention
For each arm, outcome of interest and time period
Sample size (n analysed)
Mean
SD
Other info
Any other work-related instruments or scales used
Any comments notes about results
Conflicts of interest
References to other relevant studies
Correspondence with the author(s) required?
If correspondence needed, make a note as to why
Name of corresponding author
E-mail of corresponding author
Which of the following sources were *obtained* to help inform the risk-of-bias assessment?

Risk of bias evaluation

We made the following assumptions when responding to the signalling questions of RoB2:

1. **Risk of bias due to deviations from the intended interventions**: We assumed deviations arose because of the trial context when the study used a waitlist control group where the waitlist period was at least 6 months. We argued that the long delay may have compelled some control group participants to seek a MBP or other mental health interventions independently. Similarly, we assumed there to be a possibility of deviations where no intervention control was used – that is, the control group did not receive any intervention even after the end of the study.

2. **Risk of bias due to missing data**: For 3.3/3.4 we assumed missingness could depend on true value when the outcome measure was self-reported or reported by an observer where the participant needed to approve the observation. However, we responded "no information" for the likelihood of that dependence, unless triallists provided relevant evidence.

3. **Risk of bias in measurement of the outcome**:

    a. For 4.2, we responded "probably no" unless triallists explicitly described whether the measurements of outcomes differed between groups. This aspect is rarely described in the studies we included, and this rating allowed us to be open to all three levels of risk (low, some, high) following the suggested judgement algorithm.

    b. 4.4/4.5, we assumed assessment could have been influenced by knowledge of intervention when the outcome measure was self-reported. "No information" was the default for the likelihood of the influence.

For judgements of risk of bias, we followed the algorithm suggested by the tool, except for judgements of risk of bias of the reported result. The tool suggests that if all items receive a "no information" rating, the overall judgement should be "some concerns". Given the lack of pre-specified analyses plans can lead to a high risk for questionable research practices (John et al., 2012), we deemed studies with no pre-registered information available on their outcomes and analyses plans as high risk of bias.

Evaluation items

Covidence #
Study ID
1.1 Was the allocation sequence random?
1.1 Was the allocation sequence random? supporting text
1.2 Was the allocation sequence concealed until participants were enrolled and assigned to interventions?
1.2 Was the allocation sequence concealed until participants were enrolled and assigned to interventions? supporting text
1.3 Did baseline differences between intervention groups suggest a problem with the randomization process?
1.3 Did baseline differences between intervention groups suggest a problem with the randomization process? supporting text
Risk-of-bias judgement risk of bias arising from the randomization process
Risk-of-bias judgement risk of bias arising from the randomization process supporting text
1.4 Optional: What is the predicted direction of bias due to selection of the reported result?
1.4 Optional: What is the predicted direction of bias due to selection of the reported result? supporting text
2.1a. Were participants aware of their assigned intervention during the trial?
2.1a. Were participants aware of their assigned intervention during the trial? supporting text
2.2a. Were carers and people delivering the interventions aware of participants' assigned intervention during the trial?
2.2a. Were carers and people delivering the interventions aware of participants' assigned intervention during the trial? supporting text
2.3a. If Y/PY/NI to 2.1 or 2.2: Were there deviations from the intended intervention that arose because of the trial context?

2.3a. If Y/PY/NI to 2.1 or 2.2: Were there deviations from the intended intervention that arose because of the trial context? supporting text
2.4a If Y/PY to 2.3: Were these deviations likely to have affected the outcome?
2.4a If Y/PY to 2.3: Were these deviations likely to have affected the outcome? supporting text
2.5a. If Y/PY/NI to 2.4: Were these deviations from intended intervention balanced between groups?
2.5a. If Y/PY/NI to 2.4: Were these deviations from intended intervention balanced between groups? supporting text
2.6a Was an appropriate analysis used to estimate the effect of assignment to intervention?
2.6a Was an appropriate analysis used to estimate the effect of assignment to intervention? supporting text
2.7a If N/PN/NI to 2.6: Was there potential for a substantial impact (on the result) of the failure to analyse participants in the group to which they were randomized?
2.7a If N/PN/NI to 2.6: Was there potential for a substantial impact (on the result) of the failure to analyse participants in the group to which they were randomized? supporting text
2.8a. Risk-of-bias judgement for risk of bias due to deviations from the intended interventions (effect of assignment to intervention)
2.8a. Risk-of-bias judgement for risk of bias due to deviations from the intended interventions (effect of assignment to intervention) supporting text
2.9.a Optional: What is the predicted direction of bias due to selection of the reported result?
2.9.a Optional: What is the predicted direction of bias due to selection of the reported result? supporting text
3.1 Were data for this outcome available for all, or nearly all, participants randomized?
3.1 Were data for this outcome available for all, or nearly all, participants randomized? supporting text
3.2 If N/PN/NI to 3.1: Is there evidence that the result was not biased by missing outcome data?
3.2 If N/PN/NI to 3.1: Is there evidence that the result was not biased by missing outcome data? supporting text
3.3 If N/PN to 3.2: Could missingness in the outcome depend on its true value?
3.3 If N/PN to 3.2: Could missingness in the outcome depend on its true value? supporting text
3.4 If Y/PY/NI to 3.3: Is it likely that missingness in the outcome depended on its true value?
3.4 If Y/PY/NI to 3.3: Is it likely that missingness in the outcome depended on its true value? supporting text
Risk-of-bias judgement for risk of bias due to missing outcome data
Risk-of-bias judgement for risk of bias due to missing outcome data supporting text
3.5. Optional: What is the predicted direction of bias due to selection of the reported result?
3.5. Optional: What is the predicted direction of bias due to selection of the reported result? supporting text
4.1 Was the method of measuring the outcome inappropriate?
4.1 Was the method of measuring the outcome inappropriate? supporting text
4.2 Could measurement or ascertainment of the outcome have differed between intervention groups?
4.2 Could measurement or ascertainment of the outcome have differed between intervention groups? supporting text
4.3 If N/PN/NI to 4.1 and 4.2: Were outcome assessors aware of the intervention received by study participants?
4.3 If N/PN/NI to 4.1 and 4.2: Were outcome assessors aware of the intervention received by study participants? supporting text
4.4 If Y/PY/NI to 4.3: Could assessment of the outcome have been influenced by knowledge of intervention received?
4.4 If Y/PY/NI to 4.3: Could assessment of the outcome have been influenced by knowledge of intervention received? supporting text
4.5 If Y/PY/NI to 4.4: Is it likely that assessment of the outcome was influenced by knowledge of intervention received?

4.5 If Y/PY/NI to 4.4: Is it likely that assessment of the outcome was influenced by knowledge of intervention received? supporting text
Risk-of-bias judgement for risk of bias in measurement of the outcome
Risk-of-bias judgement for risk of bias in measurement of the outcome supporting text
4.6. Optional: What is the predicted direction of bias due to selection of the reported result?
4.6. Optional: What is the predicted direction of bias due to selection of the reported result? supporting text
5.1 Were the data that produced this result analysed in accordance with a pre-specified analysis plan that was finalized before unblinded outcome data were available for analysis?
5.1 Were the data that produced this result analysed in accordance with a pre-specified analysis plan that was finalized before unblinded outcome data were available for analysis? supporting text
5.2 Is the numerical result being assessed likely to have been selected, on the basis of the results, from multiple eligible outcome measurements (e.g. scales, definitions, time points) within the outcome domain?
5.2 Is the numerical result being assessed likely to have been selected, on the basis of the results, from multiple eligible outcome measurements (e.g. scales, definitions, time points) within the outcome domain? supporting text
5.3 Is the numerical result being assessed likely to have been selected, on the basis of the results, from multiple eligible analyses of the data?
5.3 Is the numerical result being assessed likely to have been selected, on the basis of the results, from multiple eligible analyses of the data? supporting text
Risk-of-bias judgement for risk of bias in selection of the reported result
Risk-of-bias judgement for risk of bias in selection of the reported result supporting text
5.4 Optional: What is the predicted direction of bias due to selection of the reported result?
5.4 Optional: What is the predicted direction of bias due to selection of the reported result? supporting text
Overall risk-of-bias judgement
Overall risk-of-bias judgement supporting text
Risk of bias because of funding
Risk of bias because of funding supporting text
Risk of bias because of vested interest
Risk of bias because of vested interest supporting text
Other sources of bias
Other sources of bias supporting text

Supplementary Material 3: Method and results

Confidence in the evidence

Table S 1. GRADE Summary Findings: Offering a mindfulness-based programme compared to no action (passive control) for general public

Outcome (follow-up)	Number of participants (studies)	Ratings for quality of evidence	Certainty	Effect size
Task performance (Up to 4 weeks post-intervention)	3241 (22)	Risk of bias: serious, Non-reporting bias: serious, Imprecision: serious, Inconsistency: serious, Indirectness: not serious, Other considerations: not serious	Very low	Hedge's g = 0.25, 95% CI: 0.06 to 0.44, 95% PI: -0.51 to 1.01
Task performance (Up to 4 weeks post-intervention)	3241 (4)	Risk of bias: serious, Non-reporting bias: serious, Imprecision: serious, Inconsistency: serious, Indirectness: not serious, Other considerations: not serious	Very low	Hedge's g = 0.12, 95% CI: -0.3 to 0.55, 95% PI: -0.93 to 1.17
Task performance (5-24 weeks post-intervention)	5940 (13)	Risk of bias: serious, Non-reporting bias: serious, Imprecision: serious, Inconsistency: serious, Indirectness: not serious, Other considerations: not serious	Very low	Hedge's g = 0.03, 95% CI: -0.1 to 0.15, 95% PI: -0.35 to 0.4
Contextual performance (Up to 4 weeks post-intervention)	4988 (18)	Risk of bias: serious, Non-reporting bias: serious, Imprecision: serious, Inconsistency: serious, Indirectness: not serious, Other considerations: not serious	Very low	Hedge's g = 0.4, 95% CI: 0.18 to 0.63, 95% PI: -0.44 to 1.25
Contextual performance (5-24 weeks post-intervention)	4770 (10)	Risk of bias: serious, Non-reporting bias: serious, Imprecision: serious, Inconsistency: serious, Indirectness: not serious, Other considerations: not serious	Very low	Hedge's g = 0.44, 95% CI: 0.13 to 0.75, 95% PI: -0.51 to 1.39
Contextual performance (5-24 weeks post-intervention)	4770 (4)	Risk of bias: serious, Non-reporting bias: serious, Imprecision: serious, Inconsistency: serious, Indirectness: not serious, Other considerations: not serious	Very low	Hedge's g = 0.04, 95% CI: -0.35 to 0.42, 95% PI: -0.8 to 0.88
Adaptive performance (Up to 4 weeks post-intervention)	12254 (24)	Risk of bias: serious, Non-reporting bias: serious, Imprecision: serious, Inconsistency: not serious, Indirectness: not serious, Other considerations: not serious	Very low	Hedge's g = 0.19, 95% CI: 0.08 to 0.3, 95% PI: -0.22 to 0.6
Adaptive performance (Up to 4 weeks post-intervention)	12254 (6)	Risk of bias: serious, Non-reporting bias: serious, Imprecision: serious,	Very low	Hedge's g = 0.02, 95% CI: -0.21 to 0.24, 95% PI: -0.43 to 0.46

Outcome (follow-up)	Number of participants (studies)	Ratings for quality of evidence	Certainty	Effect size
		Inconsistency: not serious, Indirectness: not serious, Other considerations: not serious		
Adaptive performance (Up to 4 weeks post-intervention)	12254 (3)	Risk of bias: serious, Non-reporting bias: serious, Imprecision: serious, Inconsistency: not serious, Indirectness: not serious, Other considerations: not serious	Very low	Hedge's g = 0.16, 95% CI: 0.07 to 0.26, 95% PI: -0.11 to 0.43
Adaptive performance (5-24 weeks post-intervention)	3970 (15)	Risk of bias: serious, Non-reporting bias: serious, Imprecision: serious, Inconsistency: serious, Indirectness: not serious, Other considerations: not serious	Very low	Hedge's g = 0.28, 95% CI: 0.03 to 0.53, 95% PI: -0.5 to 1.07
Counterproductive work behaviour (Up to 4 weeks post-intervention)	1419 (8)	Risk of bias: serious, Non-reporting bias: serious, Imprecision: serious, Inconsistency: serious, Indirectness: not serious, Other considerations: not serious	Very low	Hedge's g = 0.25, 95% CI: -0.23 to 0.72, 95% PI: -1.08 to 1.57

Risk of bias rating for each study

Table S 2. Risk of bias ratings

Study	Randomisation process	Deviations from intervention	Data missingness	Outcome measurement	Result selection
**Aikens 2014**	Some concerns	Some concerns	High	High	High
	Participants were randomly selected from those who signed up to the program. No information on randomisation sequence or concealment. Detailed baseline characteristics are not provided.	Participants and facilitators were aware of intervention allocation. No information about possible deviations due to trial context. ITT used.	Data missingness not reported. Missingness could be due to attrition, which in turn could have been affected by perception of intervention efficacy.	Participants' knowledge of intervention allocation could have influenced self-reported outcomes.	No pre-specified analysis plan available. Vigour full scale results not presented or analysed.
**Allexandre 2016**	Some concerns	Some concerns	High	High	High
	No information on allocation concealment. Based on Table 1 and Table 2, the groups seem similar at baseline.	Not possible to blind participants/intervention facilitators. No information about possible deviations due to the trial context. For analysis: "In this primary intent-to-treat analysis, web-based program participants with and without group support showed..."	"...data are only available for participants who were debt collectors (N = 102, 63% of participants) and who were absent fewer than 20% of the workdays in a given month." Sensitivity analysis for missing data available. Missingness could depend on the true value as poorer health could lead to sick leave.	Not clear how score was calculated, however the raw data were collected independent of the trial.	No pre-specified analysis plan available.
**AlQarni 2023**	Low	High	High	High	High
	Allocation sequence probably randomised and concealed. No apparent between-group differences in demographics or outcomes at baseline.	Participants probably blinded to respective assignments, but not clear what information was communicated. No information about possible deviations due to the trial context. Not clear if ITT or per-protocol.	High level of missingness which could be due to true value.	Participants' knowledge of intervention content could have influenced self-reported outcomes.	No pre-specified analysis plan available. Trial registered retrospectively.
**Asthana 2021**	Low	High	High	High	High
	A computer-generated randomisation sequence was used, with the allocation sequence concealed until	No information about possible deviations due to the trial context. It is not clear if ITT was used. 9 people are counted as	High level of missingness.	Not clear if assessors were aware of intervention arm.	No pre-specified analysis plan available.

	participants were assigned to interventions. Based on Table 1, the groups seem similar at baseline.	not receiving the allocated information and thus excluded from the analysis.			
**Asuero 2014**	High	High	High	High	High
	Not clear whether allocation was concealed. Also: "The number of participants in the intervention group (43) was larger than expected due to the high interest in the mindfulness educational program and the convenience of its schedule."	No information about possible deviations due to the trial context nor on the principles upon which the analysis was conducted.	Data missingness not reported. Missingness could have been due attrition, which in turn could have been affected by engagement and perceived benefit.	Participants' knowledge of intervention allocation could have influenced self-reported outcomes.	No pre-specified analysis plan available.
**Augustus 2024**	High	High	High	High	High
	No information on randomised procedure or concealment. Participants were cluster randomised by sport teams, which may have led to a difference in baseline subjective sport performance due to differences in training.	Participants and facilitators were aware of intervention allocations. No information about possible deviations due to trial context. No information on whether analysis was pre-protocol or ITT.	High level of missingness which could be due to true value.	Participants' knowledge of intervention allocation could have influenced self-reported outcomes.	No pre-specified analysis plan available.
**Balci 2023**	High	Some concerns	High	High	High
	Randomisation completed with sealed envelopes. Allocation sequence not concealed. Slight baseline differences consistent with chance.	Participants and facilitators were aware of intervention allocation. No information about possible deviations due to the trial context. ITT used.	High level of missingness which could be due to true value.	Participants' knowledge of intervention allocation could have influenced self-reported outcomes.	No pre-specified analysis plan available.
**Barczak-Scarboro 2021**	Some concerns	High	High	High	High
	No information on randomisation procedure or concealment. No apparent between-group differences in demographics or outcomes at baseline.	Participants and facilitators were aware of intervention allocation. No information about possible deviations due to the trial context. The allocation of one participant is unknown, with no information as to how this deviation affected the outcome. There is potential that failure to analyse participants in the	It is unclear how many people were randomised, with estimates varying between 217 and 232 depending on publication. Various people were excluded depending on the paper. Missingness could be due to the true value.	Participants' knowledge of intervention allocation could have influenced self-reported outcomes.	No pre-registration and the number of papers none of which report the full list of outcomes allow for a multitude of analyses

		group to which they were randomised had substantial impact on the result. ITT not used.			
**Bartlett 2017**	Low<br>"A non-research team member handled the randomization to avoid investigator selection bias." Table 2 suggests no major imbalances	Low<br>Not possible to blind participants/intervention facilitators. No information about possible deviations due to the trial context. Two participants assigned to the information control group as a reserve were transferred to the intervention group. The possibility of this happening seems to have been pre-planned. "To test robustness of findings, inverse pro-portion modelling was used to compensate for gaps in data, by proportionally weighting post-intervention scores of completers with similar pre-intervention characteristics to non-completers"	Low<br>Overall, 66% of participants in the control condition analysed at post. 100% of participants in the intervention condition. Data missingness for outcome of interest is not explicitly reported. "A negligible difference in outcomes was observed for the complete cases versus OAG analysis."	High<br>Participants' knowledge of intervention allocation could have influenced self-reported outcomes.	High<br>No pre-specified analysis plan available.
**Baumgartner 2021**	High<br>Participants randomised through random number generation. Allocation sequence was not concealed. Group sizes are uneven, and GPA is different between groups.	High<br>Participants and facilitators were aware of intervention allocation. Contamination issues are present as the active control group received "Study Skills", a course adapted from Freshman Seminar that is offered to all incoming students. The sample was 72% freshman, so 100% of participants either attended the seminar the same year or had done so previously.	High<br>High level of missingness which could be due to true value.	Low<br>Assessors were likely unaware of participant allocation, assuming university markers are not aware of those involved in the study.	Some concerns<br>No pre-specified analysis plan available. However, in the context of the study, GPA is the only appropriate outcome measure.

**Bellosta-Batalla 2021**	Some concerns<br>No information on randomisation procedure or concealment. No apparent between-group differences in demographics or outcomes at baseline.	High<br>Not possible to blind participants/intervention facilitators. No information about possible deviations due to the trial context. Analysis conducted was per-protocol.	High<br>Data missingness not reported. Missingness could have been due attrition, which in turn could have been affected by engagement and perceived benefit.	Some concerns<br>It is not clear who scored the subscales. It is stated that "These subscales were corrected by an external researcher who was previously trained in the PIC-A correction manual. She was not informed that there were different groups in the study, thus avoiding possible biases in the evaluation process." This could have been a mistranslation.	High<br>No pre-specified analysis plan available.
**Benn 2012**	Some concerns<br>Concealment unclear. "Following randomization, results showed that treatment and control participants did not significantly differ on any baseline measures" - not clear if powered enough. Tests run for differences, not equivalences.	High<br>Not possible to blind participants/intervention facilitators. No information about possible deviations due to the trial context. Not reported whether the analysis was ITT or per-protocol	High<br>"Of the study sample, 14% declined participation after randomization (see Table 4). One treatment participant dropped out of the study after the intervention training began." Participants who dropped out had statistically significantly higher scores for depression, stress, anxiety, negative affect and lower scores for mindfulness, positive affect and personal growth ($p < .05$).	High<br>Participants' knowledge of intervention allocation could have influenced self-reported outcomes.	High<br>No pre-specified analysis plan available.
**Bonde 2022**	Some concerns<br>No information on randomisation procedure or concealment. No apparent between-group differences for the key indicator.	Some concerns<br>Participants and facilitators were aware of intervention allocation. ITT used.	High<br>High missingness. "Of the 97 school teachers allocated to the intervention group, 78 (82%) participated in an MBSR course (figure 1). None of the 78 participants attended fewer than five MBSR sessions with an average attendance of 7.6 sessions out of 9." Loss to 6-month follow-up showed statistically significant differences in age, geographical	High<br>Participants' knowledge of intervention allocation could have influenced self-reported outcomes.	High<br>No pre-specified analysis plan available.

			region, sleepiness and bodily awareness.		
**Braun 2020a**	High<br>No information on randomisation procedure or concealment. The groups were not of equal sizes. It is not known whether the randomisation was intended to be done at 1:1 ratio or not. Caregivers' relationship to the person they cared for also differed: in one group, 52.2% were spouses, in the other 26.7% were spouses. Also, in the MBSR group, nearly all (95.7 or 22 of 23 participants) were the main care providers. In the control condition, 66.7% described themselves as the main caregivers.	High<br>Not possible to blind participants/intervention facilitators. No information about possible deviations due to the trial context. "To permit investigation of the intent-to-treat (ITT) sample, all available participant data were included in the analyses"	High<br>Data missingness not reported. Missingness could have been due attrition, which in turn could have been affected by engagement and perceived benefit.	High<br>Participants' knowledge of intervention allocation could have influenced self-reported outcomes. However, "After randomization and before interventions were begun, participants were asked to rate, on a 1 (not at all) to 10 (extremely) scale the extent to which they expected their assigned course to benefit them. MBSR and SS participants did not differ on expected benefit (MBSR M = 8.0, SD = 1.95; SS M = 8.6, SD = 1.40, p = .32). ". No info on power.	High<br>No pre-specified analysis plan available.
**Braun 2020b**	High<br>Due to the randomisation procedure, unlikely that allocation was concealed. Baseline imbalances not evident	High<br>No information about possible deviations due to the trial context. "Preferential group allocation was offered /…/ if [participants'] schedules did not allow them to participate in the group to which they were randomized". 13 completed post assessment in MBP; 22 analyses in full ITT - 19 completed post assessment (CONTROL); 26 analysed in full ITT.	High<br>Data missingness not reported. Missingness could have been due attrition, which in turn could have been affected by engagement and perceived benefit.	High<br>Participants' knowledge of intervention allocation could have influenced self-reported outcomes.	High<br>No pre-specified analysis plan available. Productivity is not listed as an outcome measure in the trial registration.

**Brown 2016**	High<br>No information on randomisation procedure or concealment. The groups were not of equal sizes. It is not known whether the randomisation was intended to be done at 1:1 ratio or not. Caregivers' relationship to the person they cared for also differed: in one group, 52.2% were spouses, in the other 26.7% were spouses. Also, in the MBSR group, nearly all (95.7 or 22 of 23 participants) were the main care providers. In the control confition, 66.7% described themselves as the main caregivers.	Some concerns<br>Not possible to blind participants/intervention facilitators. No information about possible deviations due to the trial context. "To permit investigation of the intent-to-treat (ITT) sample, all available participant data were included in the analyses"	High<br>Data missingness not reported. Missingness could have been due attrition, which in turn could have been affected by engagement and perceived benefit.	Low<br>Self-reported measure used, thus blinding towards assignment was impossible. However, "After randomization and before interventions were begun, participants were asked to rate, on a 1 (not at all) to 10 (extremely) scale the extent to which they expected their assigned course to benefit them. MBSR and SS participants did not differ on expected benefit (MBSR M = 8.0, SD = 1.95; SS M = 8.6, SD = 1.40, p = .32). ". No info on power.	High<br>No pre-specified analysis plan available.
**Calcagni 2021**	High<br>No information on randomisation or concealment. No information on baseline group differences.	High<br>Participants and facilitators aware of intervention allocations. Not reported whether analysis was ITT or per-protocol.	High<br>Data missingness not reported.	High<br>Participants' knowledge of intervention allocation could have influenced self-reported outcomes.	High<br>No pre-specified analysis plan available.
**Can Gür 2020**	Low<br>"The allocation was concealed using opaque sealed envelopes." No information on baseline differences between groups - age, gender, ethnicity are all calculated for the whole group and not divided between control and intervention	High<br>Not possible to blind participants/intervention facilitators. No information about possible deviations due to the trial context. Control group seem to have received no intervention (including after trial finished) and may have sought an intervention elsewhere. Not reported whether the analysis was ITT or per-protocol, but based on Figure 1, seems to be ITT.	High<br>Data missingness not reported. Missingness could have been due attrition, which in turn could have been affected by engagement and perceived benefit.	High<br>Participants' knowledge of intervention allocation could have influenced self-reported outcomes.	High<br>No pre-specified analysis plan available.

**Chan 2021**	Some concerns	Some concerns	High	High	High
	No information on concealment. Some differences between groups at baseline, some statistically significant although lack of power and multiple testing makes it hard to judge real differences.	Not possible to blind participants/intervention facilitators. No information about possible deviations due to the trial context. ITT analysis using repeated measures ANOVA.	Data missingness not reported. Missingness could have been due attrition, which in turn could have been affected by engagement and perceived benefit.	Participants' knowledge of intervention allocation could have influenced self-reported outcomes.	No pre-specified analysis plan available.
**Choi 2022**	Some concerns	High	High	High	High
	No information on concealment or randomisation sequence. "... which involved random assignment to an 8-week mindfulness training group, Pilates training (active control), or a waitlist control group"" (p. 455)." No information on baseline group differences.	Participants and facilitators aware of intervention allocations. No information about possible deviations due to the trial context. ITT not used.	High missingness. > 30% dropout/missing data in all three groups.	Participants' knowledge of intervention allocation could have influenced self-reported outcomes.	No pre-specified analysis plan available.
**Choi 2024**	Some concerns	High		High	High
	No information on randomisation procedure or concealment. No apparent between-group differences in demographics or outcomes at baseline.	Participants and facilitators were aware of intervention allocations. No information about possible deviations due to trial context. No information on whether analysis was pre-protocol or ITT.	High level of missingness which could be due to true value.	Participants' knowledge of intervention allocation could have influenced self-reported outcomes.	No pre-specified analysis plan available.
**Christodoulou 2024**	High	High	High	High	High
	Allocation sequence was not concealed. No apparent between-group differences in outcome, however age and gender characteristics of each group were not reported.	Participants and facilitators were aware of intervention allocations. Baseline measures completed after randomisation. ITT not used.	Data missingness not reported. Missingness could have been due attrition, which in turn could have been affected by engagement and perceived benefit.	Participants' knowledge of intervention allocation could have influenced self-reported outcomes.	No pre-specified analysis plan available.

**Christopher 2018**	Some concerns<br>No information on concealment. Groups differed at baseline in the belief that MBP improves job stress, performance and resilience (favours control), MBP participants higher in self-compassion and resilience. Not enough info to suggest a problem in randomisation.	Some concerns<br>Not possible to blind participants/intervention facilitators. No information about possible deviations due to the trial context. "Intent-to-treat (ITT) analyses, without imputed missing data, assessed pre-training between-group differences for all outcomes, demographic variables, and expectancy data"	High<br>"Conclusions with imputed data differed for four outcomes (see Table 3)". Authors ran Little's missingness test and conclude data to miss at random. Power analysis not reported. Some participants dropped out because "they did not want to continue with MBRT".	High<br>Participants' knowledge of intervention allocation could have influenced self-reported outcomes.	High<br>No pre-specified analysis plan available.
**Christopher 2024**	Some concerns<br>No information on randomisation or concealment. Unclear if details in SAP were followed. No apparent between-group imbalances.	High<br>Participants and facilitators were aware of intervention allocations. No information about possible deviations due to trial context. ITT not used.	High<br>High level of missingness which could be due to true value.	High<br>Participants' knowledge of intervention allocation could have influenced self-reported outcomes.	Low<br>Analyses seem to follow SAP.
**Daigle 2018**	Some concerns<br>No info on how participants were randomised except that they were and that they were matched for scores of a measure on burnout. Not reported which measure for burnout was used. No apparent between-group imbalances.	Some concerns<br>Not possible to blind participants/intervention facilitators. No information about possible deviations due to the trial context. "ANCOVAs were performed using intention-to-treat analyses with the last observation carried forward method and pretest scores as covariates."	High<br>Nursing Errors Rating Scale data available for 28 out of 70 participants. "The Nursing Errors Rating Scale was sent by mail 3 months following MBSR to nurses in the second and third recruitment wave as part of this pilot study." Control group did not receive the measure.	High<br>Participants' knowledge of intervention allocation could have influenced self-reported outcomes. Measure only collected from intervention group. The rating scale used by the Nursing Errors Rating Scale is 0 - was never a problem to 5 - greatly improved. There seems to be no way in indicating worsening in nursing errors thus also creating an expectation of the outcome.	High<br>No pre-specified analysis plan available.
**de Carvalho 2021**	**High**<br>"Upon registration (prior to pretest), a serial number was allocated to each teacher (their students and parents). The odd numbers were assigned to the EG and the	**High**<br>Participants and facilitators aware of intervention allocations. No information about possible deviations due to the trial context. ITT not used.	**High**<br>Data missingness not reported. Missingness could have been due attrition, which in turn could have been affected by engagement and perceived benefit.	**High**<br>Participants' knowledge of intervention allocation could have influenced self-reported outcomes.	**High**<br>No pre-specified analysis plan available.

	even numbers to the waitlist CG." Allocation was not concealed. Intervention groups were imbalanced without explanation: 123 teachers allocated to intervention; 105 to control.				
**de Jong 2013**	High	High	High	High	High
	No information on randomisation procedure or concealment. No apparent between-group differences in demographics or outcomes at baseline.	No information about possible deviations due to the trial context but the control group received no intervention and were given no intervention after the study which may have motivated them to seek mindfulness elsewhere. It seems that per protocol analysis is run since everyone who stopped the course also did not provide data post-intervention.	Data missingness not reported. Missingness could have been due attrition, which in turn could have been affected by engagement and perceived benefit.	Attrition is high: 33% in the experimental group and 23% in the control group. Participants' knowledge of intervention allocation could have influenced self-reported outcomes. Quitting the training was due to finding a new job and some gave up because of private matter	No pre-specified analysis plan available.
**Desai 2024**	High	High	High	High	
	No information on randomisation procedure. Randomisation was conducted 'by the principal investigator with the help of a statistician'. No apparent between-group differences in outcomes at baseline.	Participants and facilitators were aware of intervention allocation. No information on possible deviations due to trial context. Analysis was pre-protocol with only 38% of the intervention group and 42% of the control group analysed.	High level of missingness which could be due to true value.	Attrition is high: 62% in the experimental group and 58% in the control group. Participants' knowledge of intervention allocation could have influenced self-reported outcomes.	No pre-specified analysis plan available. Subscale scores reported where total score was possible to calculate.
**dos Santos 2024**	High	High	High	High	High
	Randomisation conducted through a 'simple draw' by a member of the research team and was therefore not concealed. No apparent group differences at baseline.	Participants and facilitators were aware of intervention allocation. No information on possible deviations due to trial context. ITT not used.	Missingness not reported.	Participants' knowledge of intervention allocation could have influenced self-reported outcomes.	No pre-specified analysis plan available.

**Dvoráková 2017**	High<br>Allocation probably not concealed as participants were sent e-mails about their allocation. Also, from the flow chart (Figure 1) it appears participants were first randomised and then they were asked to fill in the pre-intervention questionnaires. The intervention group baseline scores for mental health problems are higher in every domain measured and they lower scores for scales that could correlate with wellbeing. They were also significantly more likely to attend therapy in the 6 months prior to data collection.	High<br>No information about possible deviations due to the trial context but waitlist group had to wait more than 6 months to receive intervention. "The analysis was conducted as an intent-to-treat, including all randomized participants."	High<br>Data missingness not reported. Missingness could have been due attrition, which in turn could have been affected by engagement and perceived benefit.	High<br>Participants' knowledge of intervention allocation could have influenced self-reported outcomes.	High<br>No pre-specified analysis plan available.
**Erden 2023**	Low<br>No information on randomisation. Allocation claimed to be concealed. No between-group baseline differences.	Some concerns<br>Participants and facilitators aware of intervention allocation. No information about possible deviations due to trial context. ITT was likely used.	High<br>Missingness not reported, no evidence that the result is not biased. Missingness could depend on true value.	High<br>Participants' knowledge of intervention allocation could have influenced self-reported outcomes.	High<br>No pre-specified analysis plan available.
**Erogul 2014**	High<br>Participants were first randomised and then asked whether they want to participate in the study.	High<br>Not possible to blind participants/intervention facilitators. No information about possible deviations due to the trial context. Not reported whether the analysis was ITT or per-protocol	High<br>Data missingness not reported. Missingness could have been due attrition, which in turn could have been affected by engagement and perceived benefit.	High<br>Participants' knowledge of intervention allocation could have influenced self-reported outcomes.	High<br>No pre-specified analysis plan available.

**Fazia 2023**	High	Some concerns	High	High	High
	Randomisation was completed in R, meaning concealment was likely impossible. No apparent between-group differences in demographics or outcomes at baseline.	Not possible to blind participants/intervention facilitators. No information about possible deviations due to the trial context. ITT was likely used.	High missingness which could depend on true value.	Participants' knowledge of intervention allocation could have influenced self-reported outcomes.	Trial protocol published retrospectively. No pre-specified analysis plan available.
**Flook 2013**	Some concerns	High	High	High	High
	No information on randomisation procedure or concealment. No apparent between-group differences in demographics or outcomes at baseline.	Not possible to blind participants/intervention facilitators. No information about possible deviations due to the trial context. Not reported whether the analysis was ITT or per-protocol	Data missingness not reported. As CLASS is not self-reported, missingness could come from abandoning the study (none reported) or the rater not completing CLASS. Participants could have withdrawn their consent to have an observer.	Coders were blind to study hypothesis, no info on group allocation	No pre-specified analysis plan available.
**Fraiman 2022**	High	Some concerns	Low	High	High
	Allocation sequence not concealed.	Participants and facilitators were aware of intervention allocation. ITT was likely used.	"Because 29.8% of data was missing, we conducted all multivariable analyses on 20 imputed data sets created using individual-level data following standard imputation procedures using PROC MI and PROC MI ANALYZE in SAS software"	Participants' knowledge of intervention allocation could have influenced self-reported outcomes.	No pre-specified analysis plan available. Two scales preregistered, one not used, the other one used partially (2 subscales). Evidence of multiple analyses yet alpha levels not adjusted.
**Galante 2018**	Low	Some concerns	Low	Low	Low
	"...the allocation process was concealed from the researchers." No apparent between-group imbalances	Not possible to blind participants/intervention facilitators. Some participants in the control group engaged in mindfulness elsewhere. Sensitivity analysis done.	"Examination results graded according to the British undergraduate degree classification system (examination ranking was unavailable);" Some participants were postgraduates (master's and PhD students) and had thus no examination results available. The missingness did not depend on the true value.	Examiners were unaware of which students participated in the study, including which group participants were allocated to.	No other relevant measure was planned to be collected, as per the trial protocol. Data analysed according to plan

**Garrote-Caparros 2022**	Some concerns<br>Allocation sequence not concealed. No clear imbalance between arms.	Some concerns<br>Participants and facilitators were aware of intervention allocation. ITT was likely used.	Low<br>High missingness but imputed datasets used.	Low<br>Outcome assessors not aware of group allocation	High<br>No pre-specified analysis plan available.
**Glass 2019**	High<br>Not clear whether allocation was concealed. Tested for differences between groups at baseline. No differences found, unclear whether analysis was well-powered.	High<br>Not possible to blind participants/intervention facilitators. No information about possible deviations due to the trial context. Only per-protocol data available.	High<br>Data missingness not reported. Data only available for per-protocol participants and only at 2 out of 4 time points collected	High<br>Not known whether trainer (reporter) knew about group allocation	High<br>No pre-specified analysis plan available. No ITT data reported, no control group data available
**Godara 2024**	High<br>Allocation sequence not concealed. Baseline imbalances suggest a problem with randomisation.	Some concerns<br>Not possible to blind participants/intervention facilitators. No information about possible deviations due to the trial context. ITT used.	High<br>Considerable missingness that could depend on true value.	High<br>Participants' knowledge of intervention allocation could have influenced self-reported outcomes.	High<br>No pre-specified analysis plan available.
**Gómez-Odriozola 2019**	Some concerns<br>No information on randomisation procedure or concealment. No apparent between-group differences in demographics or outcomes at baseline.	High<br>Not possible to blind participants/intervention facilitators. No information about possible deviations due to the trial context. Based on the CONSORT diagram, probably per protocol analysis	High<br>Data missingness not reported. Likely that people who did not complete treatment were not invited to fill in measures.	High<br>Participants' knowledge of intervention allocation could have influenced self-reported outcomes.	High<br>No pre-specified analysis plan available.
**Grupe 2021**	Some concerns<br>No information on randomisation sequence or concealment. "Block randomization to mindfulness training or waitlist control (stratified by police agency) occurred 1week before classes began"	Some concerns<br>Participants and facilitators aware of intervention allocations. No information about possible deviations due to the trial context. ITT used.	High<br>Data missingness not reported. Over 95% participants retained at T2 (MT = 54, WLC = 56). However, no information on whether all the participants left gave complete outcome data for the Work Limitations questionnaire.	High<br>Participants' knowledge of intervention allocation could have influenced self-reported outcomes.	High<br>No pre-specified data analysis plan for the outcome of interest. Evidence that analysis was done after data were seen.
**Hillhouse 2023**	High<br>Allocation sequence not concealed. Baseline imbalances between groups suggest a problem with randomisation.	High<br>Participants and facilitators aware of intervention allocations. No information about possible deviations due	High<br>High missingness that could depend on true value.	High<br>Participants' knowledge of intervention allocation could have influenced self-reported outcomes. Measurement of	High<br>No pre-specified analysis plan available.

		to the trial context. ITT not used.		outcome likely differed between groups	
**Huberty 2022**	High	High	High	High	High
	Allocation sequence was concealed and randomised through computer generation. Group differences at baseline were seen across all health indicators.	Participants and facilitators aware of intervention allocations. Deviations due to trial context were present: "due to low enrolment, we quickly transitioned to offering sleep coaching to all participants with an elevated ISI".	Data missingness not reported. Extent of missingness does not significantly differ between intervention and control group (32% vs 34%) for all available data.	Participants' knowledge of intervention allocation could have influenced self-reported outcomes.	No pre-specified analysis plan available.
**Hunsinger 2019**	Some concerns	High	High	High	High
	No information on randomisation procedure or concealment. No apparent between-group differences in demographics or outcomes at baseline.	Not possible to blind participants/intervention facilitators. No information about possible deviations due to the trial context but no-intervention control may have led some participants to seek MBP elsewhere. ITT used.	Data missingness not reported. Large attrition. Outcome measured with a behavioural task, not clear how the task was explained to the participants	Implicit bias is unlikely to be a good measure for racial bias (e.g., https://replicationindex.com/category/implicit-bias/).	No pre-specified analysis plan available.
**Hwang 2019**	High	High	High	High	High
	No information on randomisation procedure or concealment. School types considerably differed between intervention and control group.	Not possible to blind participants/intervention facilitators. No information about possible deviations due to the trial context but waitlist control was given access to the MBP more than 6 months after randomisation. Not reported whether the analysis was ITT or per-protocol.	6% data missing, mainly at outcome level.	Participants' knowledge of intervention allocation could have influenced self-reported outcomes.	No pre-specified analysis plan available.
**Janssen 2022**	Some concerns	High	High	High	High
	Allocation sequence was randomised and concealed, but no information on how allocation was generated. No baseline group differences on outcome measures, but significant differences evident between courses.	Participants and facilitators were not blinded to intervention allocation. High risk due to per-protocol analysis method.	Data missingness not reported. High dropout with no sensitivity analysis. Reasons for drop-out that could be related to the outcome include "not fulfilling expectations", and "lack of motivation."	Outcome assessors were aware of assignment. No information provided on whether knowledge of intervention impacted assessment outcome.	Protocol states ITT analysis will be conducted, but only per-protocol analysis is reported. Subscale scores were used instead of full scores.

**Jennings 2013**	High	High	High	High	High
	No information on randomisation procedure or concealment. Not clear how many participants were allocated to which groups.	Not possible to blind participants/intervention facilitators. No information about possible deviations due to the trial context. Not reported whether the analysis was ITT or per-protocol	Data missingness not reported, not clear how many participants were assigned to groups	Participants' knowledge of intervention allocation could have influenced self-reported outcomes.	No pre-specified analysis plan available.
**Jennings 2017**	Some concerns	High	High	Low	High
	No information on randomisation or concealment. No apparent baseline between-group differences in demographics or outcomes.	Not possible to blind participants/intervention facilitators. No information about possible deviations due to the trial context. Not reported whether the analysis was ITT or per-protocol	Data missingness not reported. Teachers who were not happy to be observed and rated using the CLASS could have withdrawn their consent to be observed. Missingness is not reported so unclear how many if any participants did that.	Observations were conducted by 24 ethnically diverse certified coders who were blind to teacher intervention condition.	No pre-specified analysis plan available.
**Jia-Yuan 2022**	Some concerns	Some concerns	High	High	High
	No information on randomisation or concealment. No apparent baseline between-group differences in demographics or outcomes.	Not possible to blind participants/intervention facilitators. No information about possible deviations due to the trial context. ITT used.	Data missingness not reported. Missingness could have been due attrition, which in turn could have been affected by engagement and perceived benefit.	Participants' knowledge of intervention allocation could have influenced self-reported outcomes.	No pre-specified analysis plan available.
**Juul 2021**	High	Some concerns	Low	High	Some concerns
	No information on randomisation. Allocation sequence was not concealed. Between-group differences were present across all four mental health measures.	Not possible to blind participants/intervention facilitators. No information about possible deviations due to the trial context. ITT used.	Some missingness. "Furthermore, the loss to follow-up in the RCT was relatively small and non-differential with no specific measured characteristics of the students leaving the study, making the risk of selection bias low. This was supported by the results of a rigorous sensitivity analysis."	Participants' knowledge of intervention allocation could have influenced self-reported outcomes.	No pre-specified analysis plan available. Trial registration specifies the outcome of interest at the given timepoint.
**Karing 2021**	Some concerns	Some concerns	High	High	High
	No information on randomisation or concealment. No apparent baseline between-group differences in demographics or outcomes.	Not possible to blind participants/intervention facilitators. No information about possible deviations due to the trial context. ITT used.	High level of missingness.	Participants' knowledge of intervention allocation could have influenced self-reported outcomes.	No pre-specified analysis plan available.

**Klatt 2015**	Some concerns<br>No information on randomisation procedure, concealment, or between-group differences at baseline.	High<br>Not possible to blind participants/intervention facilitators. No information about possible deviations due to the trial context. Not reported whether the analysis was ITT or per-protocol.	High<br>Data missingness not reported. Missingness could have been due attrition, which in turn could have been affected by engagement and perceived benefit.	High<br>Participants' knowledge of intervention allocation could have influenced self-reported outcomes.	High<br>No pre-specified analysis plan available.
**Klatt 2017**	High<br>No information on randomisation concealment. Group allocation is said to have been stratified based on sex, yet there are considerable differences in the proportion of males and females in the two groups (int: 22% male; control 40% male).	High<br>Not possible to blind participants/intervention facilitators. No information about possible deviations due to the trial context. Probably per-protocol analysis, based on CONSORT diagram	High<br>Data missingness not reported. Reasons to discontinue with study (n = 20) included work-related conflict, uninterested, injury.	High<br>Participants' knowledge of intervention allocation could have influenced self-reported outcomes. Participants in the control group did not participate in the 9-week follow up.	High<br>No pre-specified analysis plan available. From Table 1 it seems data was also collected on presenteeism, absenteeism, capacity to work, and ability to perform daily life activities among other things. These data are only presented for baseline.
**Kor 2019**	Low<br>"An independent research assistant randomized the subjects /.../ using the computer- generated random numbers /.../. The participants would be informed of their group allocation via a sealed opaque envelope, which was concealed to the researchers and the assessors". No information on baseline imbalances.	Some concerns<br>Not possible to blind participants/intervention facilitators. No information about possible deviations due to the trial context. ITT used.	High<br>Data missingness not reported. Missingness could have been due attrition, which in turn could have been affected by engagement and perceived benefit.	High<br>Participants' knowledge of intervention allocation could have influenced self-reported outcomes.	High<br>No pre-specified analysis plan available.

**Kor 2020**	Low<br>Online sequence generation randomization tool was used. Participants "received notice of their group allocation in an opaque, sealed envelope /…/. The group allocation lists were concealed from the researchers, the staff of the older people centres, and the outcome assessors".	Some concerns<br>Not possible to blind participants/intervention facilitators. No information about possible deviations due to the trial context. ITT used.	High<br>Data missingness not reported. Missingness could have been due attrition, which in turn could have been affected by engagement and perceived benefit.	High<br>Participants' knowledge of intervention allocation could have influenced self-reported outcomes.	High<br>Protocol pre-specifies MANOVA, paper reports on GEE instead.
**Külcher 2023**	Some concerns<br>No information on randomisation concealment. No apparent between-group differences in demographics or outcomes at baseline.	High<br>Not possible to blind participants/intervention facilitators. "In addition, one participant from the WL was accidentally handled as if allocated to GoD during the trial. Consequently, for the purpose of data analysis they were relocated to the GoD group." No other deviations reported. ITT used.	Low<br>At post-intervention, 152 (out of 386 allocated participants) participants were lost to follow-up (IG1=61, IG2=59, WL=32). Reasons for loss to follow-up were not reported. Missing data was imputed.	High<br>Participants' knowledge of intervention allocation could have influenced self-reported outcomes.	Some concerns<br>No pre-specified analysis plan available. Protocol received to be published in July 2019, before the completion of participant data collection in November 2020 specified the outcome measure.
**Kuyken 2022**	High<br>The allocation sequence was randomised but not concealed from the trial manager. No apparent between-group differences in demographics or outcomes at baseline.	Some concerns<br>Participants and people teaching the MBP were aware of group allocation. No deviations due to trial context reported. ITT used.	High<br>Data missingness is more than 5%. Missingness could have been due attrition, which in turn could have been affected by engagement and perceived benefit.	High<br>Participants' knowledge of intervention allocation could have influenced self-reported outcomes.	High<br>Pre-specified analysis plan is mentioned in the paper and in the trial protocol. However, it has not been published. Total score is not reported, only subscale scores.
**Lebares 2019**	Some concerns<br>The allocation sequence was randomised but no information is provided on concealment. No apparent between-group differences in demographics or outcomes at baseline.	High<br>Not possible to blind participants/intervention facilitators. One participant was initially allocated to the active control but did not receive the intervention owing to inadvertently attending the modMBSR training class during week 1. She was therefore reassigned to the modMBSR	High<br>Data missingness not reported.	High<br>No information on whether assessors were aware of the group assignment	Pre-specified analysis plan does not provide enough detail. The protocol specifies the group of tasks to be used but not which ones. The outcome measure (accuracy or timing) is not prespecified.

		intervention group. ITT not used.			
**Lensen 2024**	Some concerns	Some concerns	Low	High	Some concerns
	No information on randomisation concealment. No apparent between-group differences in demographics or outcomes at baseline.	Not possible to blind participants/intervention facilitators. No information about possible deviations due to the trial context. ITT used.	93% participants retained for the intervention group at post-measure, and 96% participants retained for the control group. Reasons for attrition were reported, which included illness, not happy with assignment to control, other task in school, stopped job, not meeting inclusion criteria (job outside education), and choosing other training.	Participants' knowledge of intervention allocation could have influenced self-reported outcomes.	The numerical result aligns with the analysis intentions from the protocol; however, the protocol was published following the availability of some of the outcome data.
**Lin 2019**	Some concerns	High	High	High	High
	No information on randomisation concealment. No apparent between-group differences in demographics or outcomes at baseline.	Not possible to blind participants/intervention facilitators. No information about possible deviations due to the trial context but long delay for waitlist control until they received intervention may have lead to participants seeking support elsewhere. Probably per-protocol analysis as 11 participants seem to have excluded due to low engagement in intervention.	Data missingness not reported but per protocol analysis excluded some participants. Missingness could have been due to attrition, which in turn could have been affected by engagement and perceived benefit.	Participants' knowledge of intervention allocation could have influenced self-reported outcomes.	No pre-specified analysis plan available.
**Liu 2022**	Some concerns	High	High	High	High
	The allocation sequence was randomised but no information is provided on concealment. No apparent between-group differences in demographics or outcomes at baseline.	Not possible to blind participants/intervention facilitators. No information about possible deviations due to the trial context.	Data missingness is not reported and could have an impact on outcomes.	Participants' knowledge of intervention allocation could have influenced self-reported outcomes.	No pre-specified analysis plan available.
**Liu 2023**	Some concerns	High	High	High	High
	No information on randomisation or concealment. No apparent	Not possible to blind participants/intervention facilitators. No information	High missingness that could depend on true value.	Participants' knowledge of intervention allocation could have influenced self-reported	No pre-specified analysis plan available.

	between-group differences in demographics or outcomes at baseline.	about possible deviations due to the trial context. ITT likely not used.		outcomes. Measurement of outcome could have differed between groups	
**Modrego-Alarcón 2021**	Some concerns	Some concerns	Low	High	High
	The allocation sequence was randomised but no information is provided on concealment. "Assignation of the subjects was performed after the baseline evaluation by a member of the research group, who had no knowledge about the study aims and was not involved in the study in any other way". No apparent between-group differences in demographics or outcomes at baseline.	Not possible to blind participants/intervention facilitators. No information about possible deviations due to the trial context. ITT used.	Attrition was reported. 64 (MBP), 68 (MBP + VR), 51 (Control) participants analysed at follow-up for UWES outcome variable. Reasons for leaving the study were reported for the study in general. Missingness corrected by multiple imputation. Sensitivity analysis performed.	Participants' knowledge of intervention allocation could have influenced self-reported outcomes.	No pre-specified analysis plan available.
**Nadler 2020**	Some concerns	High	High	High	High
	No information on randomisation procedure or concealment. No apparent between-group differences in demographics or outcomes at baseline.	Not possible to blind participants/intervention facilitators. No information about possible deviations due to the trial context. Analysis was per-protocol	Data missingness not reported, attrition high post randomisation and post intervention. Missingness could have been due attrition, which in turn could have been affected by engagement and perceived benefit.	Participants' knowledge of intervention allocation could have influenced self-reported outcomes.	No pre-specified analysis plan available.
**Nassif 2023**	High	Some concerns	High	High	High
	No information on randomisation procedure or concealment. Between-groups differences are evident in rank and age. The CONSORT flowchart suggest participants were first randomised and then consented.	Not possible to blind participants/intervention facilitators. No information about possible deviations due to the trial context. ITT used.	High missingness. At post-intervention, 152 (out of 386 allocated participants) participants were lost to follow-up (IG1=61, IG2=59, WL=32). Reasons for loss to follow-up were not reported.	Not clear whether assessors of shooting accuracy were aware of group allocation. Assessment could have been influenced by knowledge of intervention.	No pre-specified analysis plan available.
**Nielsen 2021**	High	Some concerns	High	High	High
	No information on randomisation procedure or concealment. Between-	Participants and facilitators aware of intervention allocation. No information about possible deviations due to the trial	High missingness. Chi-square and independent t-tests indicated attrition between T1 and T2 was significantly related	Outcome assessors were aware of condition, which could have influenced their rating.	Pre-specified analysis plan does not provide enough detail. Plan does not differentiate between Study 1 and Study 2.

	group differences evident on outcomes.	context. Modified ITT used, excluding participants who did not respond to the assessments.	to condition and age, with attrition being higher in the experimental condition (44.44% VS 18.75%).		Plan states ANOVA will be used, but ANCOVA was reported due to high attrition.
**Nübold 2021**	Some concerns	High	High	Low	High
	No information about how allocation sequence was generated. Allocation sequence may have been concealed. No information on baseline imbalances.	Participants were aware of intervention allocation. Not clear whether participants were excluded from analysis if they did not partake the intervention.	High missingness which could be due to the true value.	Although self-reported, use of active specific intervention reduces the risk that assessment could have influenced by knowledge of the intervention received.	No pre-specified analysis plan available. Authors state hypotheses are not pre-registered.
**Ogino 2024**	High	High	High	High	High
	No information on randomisation or concealment. Baseline imbalances between groups suggest some problems with randomisation.	Participants and facilitators not blind to intervention allocation. No information about possible deviations due to the trial context. ITT not used.	High missingness which could be due to true value.	Participants' knowledge of intervention allocation could have influenced self-reported outcomes.	No pre-specified analysis plan available.
**Orosa-Duarte 2021**	High	High	High	High	High
	Website used to randomise rules out concealment. No apparent between-group differences in demographics or outcomes at baseline.	22 of 29 participants in the IMBP group did not receive the intervention. No information is provided for the App group nor the control group. Authors claim to have done ITT but also say they excluded participants who did not complete 8 week assessment.	Data missingness not reported but attrition is high (no data available for 70 out of 154 participants). Missingness could have been due attrition, which in turn could have been affected by engagement and perceived benefit.	Participants' knowledge of intervention allocation could have influenced self-reported outcomes.	No pre-specified analysis plan available.
**Pang 2019**	High	High	Low	High	High
	Only 40% of the sample was randomised, the other received allocation based on participant availability. Concealment thus not possible. No apparent baseline between-group differences.	Not possible to blind participants/intervention facilitators. Waitlist was for 1 year and may have led to some participants seek support earlier. Although people did also pay their participation fee which reduced the likelihood of them seeking additional help. ITT performed	Data available for nearly all participants	Supervisors rated performance, it is unclear whether they were blind to allocation	No pre-specified analysis plan available.

**Perez-Blasco 2016**	High<br>Randomisation procedure did not allow for concealment. No apparent between-group differences in demographics or outcomes at baseline.	High<br>"The participants did not know which group they belonged to until the second data collection was completed". Given it was a passive control group study, it does not seem to be possible to blind participants/intervention facilitators. No information about possible deviations due to the trial context. Per-protocol analysis: "two participants did not have 90% attendance and were excluded from the analyses"	High<br>Data missingness not reported. Missingness could have been due attrition, which in turn could have been affected by engagement and perceived benefit.	High<br>Participants' knowledge of intervention allocation could have influenced self-reported outcomes.	High<br>No pre-specified analysis plan available.
**Phang 2015**	High<br>Randomisation procedure does not allow for concealment. Also, intervention group has more favourable scores in all of the 4 outcomes collected at baseline. This could also be due to randomness but the probability of all of the 4 measures favouring the intervention group is low.	High<br>Not possible to blind participants/intervention facilitators. Waitlist participants had to wait 6 months to receive intervention and may have sought support independently. ITT used.	High<br>Data missingness not reported. Missingness could have been due attrition, which in turn could have been affected by engagement and perceived benefit.	High<br>Participants' knowledge of intervention allocation could have influenced self-reported outcomes.	High<br>No pre-specified analysis plan available.

**Pipe 2009**	High<br>Participants met with the research team for a project overview prior to intervention, concealment thus unlikely. No baseline data available	High<br>Not possible to blind participants/intervention facilitators. When participants joined the study they were told there is a 1 year follow-up period. It is not clear when the decision was made to scrap it. The participants in the control condition could have sought support elsewhere given the paper's authors report high stress levels. Probably per-protocol analysis done as the person who withdrew from the study is excluded (not clear if withdrew from the intervention or the study altogether).	Low<br>Data available for nearly all participants	High<br>Participants' knowledge of intervention allocation could have influenced self-reported outcomes.	High<br>No pre-specified analysis plan available.
**Rad 2023**	Some concerns<br>No information on randomisation procedure or concealment. No apparent between-group differences in demographics or outcomes at baseline.	High<br>Participants and facilitators aware of intervention allocation. No information about possible deviations due to the trial context. ITT not used.	High<br>Missingness not reported and could depend on true value.	High<br>Participants' knowledge of intervention allocation could have influenced self-reported outcomes.	High<br>No pre-specified analysis plan available.
**Repo 2022**	High<br>Allocation was random but likely not concealed. No apparent between-group differences in demographics or outcomes at baseline.	High<br>Participants and facilitators aware of intervention allocation. No information about possible deviations due to the trial context. ITT not used.	High<br>Missingness not reported and could depend on true value.	High<br>Outcome assessors aware of group assignment which could have influenced ratings.	High<br>No pre-specified analysis plan available.
**Rich 2021**	Low<br>Randomisation done in Qualtrics which does allow for concealment. Not explicitly stated whether allocation was concealed. No apparent between-group imbalances.	Some concerns<br>Not possible to blind participants but intervention was delivered through an app. The app, "Headspace" is available for anyone to access and control group participants may have accessed it despite their group allocation. ITT used.	High<br>Data missingness not reported, but attrition high. Those who dropped out (and thus were less likely to fill in T2), also had lower job engagement.	High<br>Participants' knowledge of intervention allocation could have influenced self-reported outcomes.	High<br>No pre-specified analysis plan available.

**Rodrigues de Oliveria 2021**	High<br>Randomisation done in SPSS by a researcher who was not involved in the study. Allocation was not concealed from the trialists. No apparent between-group imbalances.	High<br>Participants and facilitators aware of intervention allocation. No information about possible deviations due to the trial context. ITT not used. "Thirty-five participants dropped out of the study because they did not complete more than three sessions."	High<br>High attrition across both groups. Reasons for attrition not reported.	High<br>Participants' knowledge of intervention allocation could have influenced self-reported outcomes.	High<br>No pre-specified analysis plan available. Outcome of interest registered retrospectively.
**Roeser 2013**	Some concerns<br>No information on randomisation but also no apparent between-group imbalances.	High<br>Not possible to blind participants/intervention facilitators. Waitlist control had to wait 6 months to access intervention and may have sought support independently. Not reported whether the analysis was ITT or per-protocol.	High<br>Data missingness not reported. But reported withdrawal reasons include not finding the intervention engaging/worthwhile and having a health crisis. These could have influenced outcomes.	High<br>Participants' knowledge of intervention allocation could have influenced self-reported outcomes.	High<br>No pre-specified analysis plan available.
**Roeser 2022**	Some concerns<br>No information on randomisation procedure or concealment. No apparent between-group differences in demographics or outcomes at baseline.	Low<br>Participants and facilitators aware of intervention allocation. Likely no possible deviations due to the trial context. ITT used.	High<br>Missingness not reported and could depend on true value.	Low<br>Precautions were taken throughout the study (e.g., separating the implementation team from the research team, restricting access to data that could identify study condition, restricting discussions about individual teachers) to keep the class-room observers blind to teachers' group assignment.	High<br>No pre-specified analysis plan available.
**Sampl 2017**	High<br>No information on randomisation procedure or concealment. Unequal group allocation (51 vs 58). This could have happened randomly but unclear. Also mean age differs. Of the measures, control group fares slightly worse outcome measures except one (self-	High<br>Not possible to blind participants/intervention facilitators. No information about possible deviations due to the trial context. Not reported whether the analysis was ITT or per-protocol.	High<br>High missingness for grades, the main outcome. The outcome measures of interest are related to ability to perform in during the exam period. If participants felt they were not doing well, they may have had less motivation to complete measures.	Low<br>Main outcome of interest was average grade - assessors were thus blind to allocation. Secondary outcomes were self-reported.	High<br>No pre-specified analysis plan available.

	leadership). There are no statistically significant differences.				
**Schroeder 2018**	Some concerns<br>No information on randomisation procedure or concealment. No apparent between-group differences in demographics or outcomes at baseline.	High<br>Not possible to blind participants/intervention facilitators. No information about possible deviations due to the trial context. It's likely that per-protocol approach was used.	Low<br>High missingness which was dealt with REML.	Low<br>Outcomes not self-reported	High<br>No pre-specified analysis plan available.
**Shapiro 1998**	Some concerns<br>No information on randomisation procedure or concealment. Between-group demographics and outcomes at baseline not available. Significance testing for differences yielded a 0-result.	High<br>Not possible to blind participants/intervention facilitators. No information about possible deviations due to the trial context. Not reported whether the analysis was ITT or per-protocol.	High<br>Data missingness not reported for outcome of interest. Missingness could have been due attrition, which in turn could have been affected by engagement and perceived benefit.	High<br>Participants' knowledge of intervention allocation could have influenced self-reported outcomes.	High<br>No pre-specified analysis plan available.
**Shapiro 2011**	Some concerns<br>No information on randomisation but also no apparent between-group imbalances.	High<br>Not possible to blind participants/intervention facilitators. No information about possible deviations due to the trial context. Not reported whether the analysis was ITT or per-protocol.	High<br>Data missingness present for several variables. Missingness could have been due attrition, which in turn could have been affected by engagement and perceived benefit.	High<br>Participants' knowledge of intervention allocation could have influenced self-reported outcomes.	High<br>No pre-specified analysis plan available.
**Shapiro 2019**	Some concerns<br>Randomisation done by computer. No information on allocation sequence, concealment, or baseline between-group differences.	High<br>Not possible to blind participants/intervention facilitators. No information about possible deviations due to the trial context. Not reported whether the analysis was ITT or per-protocol.	High<br>Data missingness not reported. Missingness could have been due attrition, which in turn could have been affected by engagement and perceived benefit.	High<br>Participants' knowledge of intervention allocation could have influenced self-reported outcomes.	High<br>No pre-specified analysis plan available.

**Steinberg 2017**	Some concerns<br>No information on randomisation concealment. No apparent between-group differences in demographics or outcomes at baseline.	Some concerns<br>Not possible to blind participants/intervention facilitators. No information about possible deviations due to the trial context. ITT used.	High<br>Data missingness not reported. Missingness could have been due attrition, which in turn could have been affected by engagement and perceived benefit.	High<br>Participants' knowledge of intervention allocation could have influenced self-reported outcomes.	High<br>No pre-specified analysis plan available. No group*time interaction analyses are presented, just time-interaction.
**Strauss 2021**	Some concerns<br>Not explicitly stated but randomisation procedure used does allow for concealment. Lack of information on baseline imbalances.	High<br>Not possible to blind participants/intervention facilitators. There were more people in the intervention group who did not receive the allocated intervention (n = 21) compared to the control group (n = 4). ITT used.	Some concerns<br>Although missingness for the outcome of interest is not clearly reported, overall loss to follow-up was around 33% in the intervention group and 24% in the control group. "Missing-values analysis revealed that stress and wellbeing met criteria for MCAR [Little's MCAR2 (df = 71) = 58.34, p = .859].	High<br>Participants' knowledge of intervention allocation could have influenced self-reported outcomes.	High<br>The protocol specifies the use of mixed ANOVA. The main outcome paper uses regression. The protocol states that "Presenteeism is measured using items from the Institute for Medical Technology Assessment Productivity Cost Questionnaire". It does not specify which items and how many. Similarly it is stated that "Compassion is measured using the Compassion Scale" without specifying which scale.
**Takhdat 2024**	Some concerns<br>No information on randomisation or concealment. No apparent between-group differences in demographics or outcomes at baseline.	High<br>Participants and facilitators were aware of intervention allocation. No information about possible deviations due to trial context. ITT not used.	High<br>High missingness which could depend on the true value.	Some concerns<br>Participant allocation was concealed from observational raters. Interrater reliability between raters was not reported.	High<br>No pre-specified analysis plan available.
**Taylor 2016**	High<br>No information on randomisation procedure or concealment. The participants assigned to the intervention group were more stressed. Also, demographic information suggest the study had 59 participants, but 56 were randomised. There is no mention what happened to the three.	High<br>Not possible to blind participants/intervention facilitators. No information about possible deviations due to the trial context. Not reported whether the analysis was ITT or per-protocol	High<br>Follow-up data is not reported for anyone. Data missingness for the outcome of interest is not reported.	High<br>Participants' knowledge of intervention allocation could have influenced self-reported outcomes.	High<br>No pre-specified analysis plan available. Follow-up data not reported although collected.

**Taylor 2022**	Low	Some concerns	High	High	High
	Randomisation done through Qualtrics which allows for concealment. No apparent between-group imbalances.	Participants were aware of intervention allocation. All but the mediation analysis was conducted blind to the study arm (p. 6). No information about possible deviations due to the trial context, however both interventions were available to engage with outside the context of the trial. No information on potential cross-over reported. ITT used.	High attrition. Only 1123 participants out of 2182 randomized completed the measurement (see CONSORT, p. 4). Reasons for attrition not reported.	Outcome assessors aware of group assignment which could have influenced ratings.	No pre-specified analysis plan available.
**Torres 2021**	Some concerns	High	High	High	High
	Outcome assessors aware of group assignment which could have influenced ratings.	Participants and facilitators aware of intervention allocation. No information about possible deviations due to the trial context. Authors claim ITT used, however participants were excluded if they did not participate enough, suggesting per-protocol analysis.	High missingness. Greater loss to follow-up in the IG2 (which was longer than IG1) compared to the other groups. Participants retained at follow-up for intervention groups were around 60%, compared to 80% for the control group. Documented reasons were also related to refusal to continue participating (control: 19%, IG1: 23%, IG2: 27%).	Outcome assessors aware of group assignment which could have influenced ratings.	No pre-specified analysis plan available. Protocol does not pre-specify which variables are included in models. Type of analysis not the same as pre-specified in protocol (planned to use multilevel linear regression but reported ANCOVA).
**Vainre 2024**	Low	Some concerns	High	High	Some concerns
	Randomisation procedure allowed for concealment. No apparent between-group differences in demographics or outcomes at baseline.	Not possible to blind participants/intervention facilitators; however, participants were not told which intervention was considered to be the control. No information about possible deviations due to the trial context. ITT used.	High missingness which could be due to true value.	Participants' knowledge of intervention allocation could have influenced self-reported outcomes.	The numerical result aligns with the analysis intentions from the protocol; however, the protocol was published following the availability of some of the outcome data.
**Valley 2017**	Some concerns	High	High	High	High
	Outcome assessors aware of group assignment which could have influenced ratings.	Not possible to blind participants/intervention facilitators. No information about possible deviations due to the trial context. Not reported	5 out of 12 participants in the control group dropped out. 6-months follow-up was collected only from those who completed the course (including wait-list controls who could do	Self-reported measure used, thus blinding towards assignment was impossible. Control group data bot collected d for follow-up	No pre-specified analysis plan available. Data were presented differently for different time-points.

		whether the analysis was ITT or per-protocol	mindfulness after post-intervention)		
**van Berkel 2014, van Dongen 2016**	High<br>Randomisation procedure probably did not allow for concealment. No apparent between-group differences in demographics or outcomes at baseline.	High<br>Not possible to blind participants/intervention facilitators. Waitlist participants needed to wait a year to access intervention and may have sought support independently. ITT used.	Low<br>Data missingness is low	High<br>Participants' knowledge of intervention allocation could have influenced self-reported outcomes.	High<br>Analysis plan available but undated. Not all outcomes of interest are specified. In van Dongen 2016, the change in WAI is said to not be statistically significant. There could therefore be a file-drawer effect as the data is not reported in other reports.
**van Dijk 2017**	High<br>Group allocation not concealed. No apparent between-group differences in demographics or outcomes at baseline.	High<br>Not possible to blind participants/intervention facilitators. Control group participants had to wait for the intervention for 6 months and may have sought support independently. ITT used.	Low<br>Data missingness not reported but sensitivity analysis run to account for their effect.	High<br>Participants' knowledge of intervention allocation could have influenced self-reported outcomes.	High<br>Pre-specified analysis plan not accessible.
**Verweij 2018**	Some concerns<br>No information on randomisation concealment but also no apparent between-group imbalances.	Some concerns<br>Not possible to blind participants/intervention facilitators. No information about possible deviations due to the trial context.	High<br>Data missingness not reported. Missingness could have been due attrition, which in turn could have been affected by engagement and perceived benefit. Sensitivity analyses were performed but not for the outcomes of interest for the current review	High<br>Participants' knowledge of intervention allocation could have influenced self-reported outcomes.	High<br>The outcome of interest scale is reported as subscales with no total score.
**Wang 2023**	Low<br>Randomisation procedure allowed for concealment. No apparent between-group differences in demographics or outcomes at baseline	High<br>Participants and facilitators aware of intervention allocation. No information about possible deviations due to the trial context. Participants excluded if they did not participate enough, suggesting per-protocol analysis. ITT not used.	High<br>High missingness which could be due to true value.	High<br>Participants' knowledge of intervention allocation could have influenced self-reported outcomes.	High<br>Trial registered retrospectively. Pre-specified analysis plan not available.

**Watson-Singleton 2024**	High<br>Allocation sequence not concealed. No apparent between-group differences in demographics or outcomes at baseline	High<br>Participants were aware of intervention allocation. Intervention was delivered via an app. No information about possible deviations due to the trial context. ITT not used.	High<br>High missingness which could be due to true value.	High<br>Participants' knowledge of intervention allocation could have influenced self-reported outcomes.	High<br>Trial registered retrospectively. Pre-specified analysis plan not available.
**Wilson 2022**	High<br>Randomisation done in SPSS, which does not allow concealment. No apparent between-group imbalances at baseline.	High<br>Participants and facilitators aware of intervention allocation. No information about possible deviations due to the trial context. Participants excluded if they did not participate enough, suggesting per-protocol analysis. ITT not used.	High<br>High missingness which could be due to true value.	High<br>Participants' knowledge of intervention allocation could have influenced self-reported outcomes.	High<br>Pre-specified analysis plan not available.

Reporting bias

Table S 3. Potentially eligible studies pre-registered in ICTRP by August 2, 2021 but not published by August 2, 2024. Their eligibility could not always be assessed due to lack of clarity in pre-registrations.

Trial ID	Title
ChiCTR2100041851	The Impact of MIndfulness Meditation on the Arthroscopy Performance in Orthopaedic Surgeons
ChiCTR2100041811	The impact of Mindfulness Meditation on the maintenance of arthroscopy skills in orthorpaedic surgeons
CTRI/2021/03/031803	Effects of Mindfulness based intervention on mental well-being and quality of life in Indian adults: a randomised controlled trial
CTRI/2021/02/031479	Effect of an Online Mindfulness program on Stress in Indian Adults during COVID19 pandemic: A randomized controlled preliminary study to search for an alternative therapy
ChiCTR2100043371	The effects of brief mindfulness meditation on attention
ChiCTR2000039347	Effect of Mindfulness-based Program Promoting College Students' Well-being
IRCT20181203041832N1	The effectiveness of online mindfulness training based on stress reduction (MBSR) on mental health and quality of work life of nurses fighting on the frontlines against COVID-19
ChiCTR2100042726	Mindfulness and Psychological Intervention Programs for College Students in the Context of COVID-19: A Randomized Controlled Trial
ChiCTR2100042344	School-Based Mindfulness Intervention with Migrant Children in China
NCT03728062	Mindfulness Meditation vs. Physical Exercise as Internal Recovery Strategies: Study on Comparative Effects on Stress, Fatigue, Burnout, Sleep Quality and Immunocompetence. A Randomized Controlled Trial
ACTRN12610000833066	A randomised controlled trial of a fully automated online mindfulness program focussing on 18-25 year TAFE and Further Education Students
ACTRN12617000049370p	Enhancing wellbeing of JMOs with mindfulness meditation pilot programme
ChiCTR1900026506	Efficacy of a Mindfulness-Based Mobile Application for Students in Tertiary Education: a Randomised Controlled Trial
ChiCTR1900026939	Efficacy of 'Mindful Flourishing' - a Mindfulness-Based Mobile Application: a Randomised Controlled Trial
IRCT201702054299N5	The effect of mindfulness intervention on job stress of nurses in intensive care units
IRCT20180429039464N3	The effect of Mindfulness- Based Stress Reduction Practice on Nurses' Self-Compassion and Job Stress of Nurses
IRCT20190604043813N1	Evaluating the effectiveness of Mindfulness-based Stress Reduction (MBSR) on occupational burnout and comparing it with control group in hospital non-medical staffs
ISRCTN03386834	Longitudinal evaluation of cost effectiveness and wellbeing related variables of mindfulness training in the workplace
ISRCTN62401721	Effects of mindfulness-based stress reduction (MBSR) on stress, depression, self-esteem and mindfulness in Thai nursing students: A randomised controlled trial
JPRN-UMIN000029791	The effect of mindfulness-based stress reduction program for workers
JPRN-UMIN000029885	Mindfulness Training in Return-to-Work Program - Mindfulness Training in Return-to-Work Program
JPRN-UMIN000031435	Mindfulness for health professionals building resilience and compassion (MHALO program) - randomized control trial - MHALO program
JPRN-UMIN000032736	Randomized Controlled Trial of Cognitive-Behavioral Therapy versus Mindfulness-Based Intervention for Depression in University Students - CBT/MBI for Depression
JPRN-UMIN000039327	Relationships among mindfulness, attention control function, mind wandering, and emotion - Relationships among mindfulness, attention control function, mind wandering, and emotion
NCT00214357	The Effects of Mindfulness Training on School Staff Emotions, Attention, and Stress
NCT01212497	Virtual Coach for Mindfulness Meditation Training
NCT04282733	Mindfulness Rounds Initiative - An 8-Week, Short Session, Mindfulness Based Protocol for On-Site Delivery of Stress Reduction Practices
NCT05397249	The Impact of Mindfulness and Spirituality on Student Well-being-Efficacy Study
NL8171	Mindfulness-Based Stress Reduction Intervention for Elementary School Teachers: Too Soft or a Solution?
NTR5001	Efficacy of a mindfulness app in promoting mindfulness, mental health, quality of life, and self-actualization.
RBR-7y89kb	The impact of mindfulness on health professionals' quality of life, anxiety, burnout levels and empathy: a randomized controlled trial

Trial ID	Title
NCT04987905	The Effect of MIND-BE Program Applied to Intensive Care Nurses on Mental Health Parameters (Mindfulness Based Empowerment)
JPRN-UMIN000044721	A randomized controlled trial examining the effect of brief mindfulness-based cognitive therapy on productivity among healthy workers - A randomized controlled trial examining the effect of brief mindfulness-based cognitive therapy on productivity among healthy workers
ACTRN12621000610831	A randomized pretest-posttest waitlist-controlled trial to examine the effects of an online mindfulness-based intervention.
IRCT20210321050755N1	The effectiveness of mindfulness practice on the stress and Psychological Capital of mothers of premature neonates admitted to the neonatal intensive care unit
ISRCTN48912721	Mindfulness-based mental agility: impact of a virtual face to face workplace intervention
NCT04929613	Intensive Mindfulness-based Resilience Training in First Responders: A Pilot Study
NCT04870775	Effectiveness of a Mindfulness Training Program for Hospital Workers During the Pandemic: A Randomised Controlled Trial
NCT04828291	Optimizing Daily Mindfulness Interventions Using Peer Support to Increase Well-Being in First-Year Students
NCT04861844	Tracking Mood and Language Change Across a Phone-based Mindfulness Intervention
NCT04610333	On Top of Everything. A Study Protocol for a Cluster-Randomised Controlled Trial Testing a Teacher Training Programme to Teach Mindfulness Among Students in Danish Upper Secondary Schools and Schools of Health and Social Care
NCT04602312	Online RCT Comparing the Effects of Mindfulness, Sham Mindfulness and Book Listening Control on Coronavirus-related Catastrophizing in Adults
NCT04594278	Remote Delivery of a Mindfulness-based Intervention to Decrease Anxiety Levels and Burnout Among Health-care Professionals During the Covid-19 Pandemic
NCT04589195	Measurement of an Application - Based Delivery of Mindfulness Training Program Content
NCT04589377	Mobile Mindfulness Training and Physics Learning
NCT04558008	Effectiveness of Online Mindfulness-based Stress Reduction Among Teachers at Upper Secondary Schools and Schools of Health and Social Care
NCT04557033	My Mindful Moments: A Mindfulness Meditation and Digital Art Activity
NCT04515667	Mindfulness in the OR: Can an Abbreviated Mindfulness Skill Improve a Surgeon's Focus, Anxiety and Performance in the OR?
NCT04518631	Effects of Mindfulness Training on Emotional Care
NCT04584268	A Randomized Controlled Trial of a Longitudinal Mindfulness Intervention to Decrease Burnout in Medical Residents
NCT04572464	A Pre-Experimental Study to Assess the Effectiveness of Mindfulness Based Guided Meditation on Wellbeing, Self Compassion and Cognizance Among Nurses Working in Intensive Care Units at ILBS, New Delhi
NCT04431297	The Impact of Virtual Mindfulness Education Sessions on Staff Perceived Stress During COVID-19
NCT04425356	Evaluation of the Effectiveness of a Mindfulness Coaching Program for Stress Management: A Randomized Control Trial

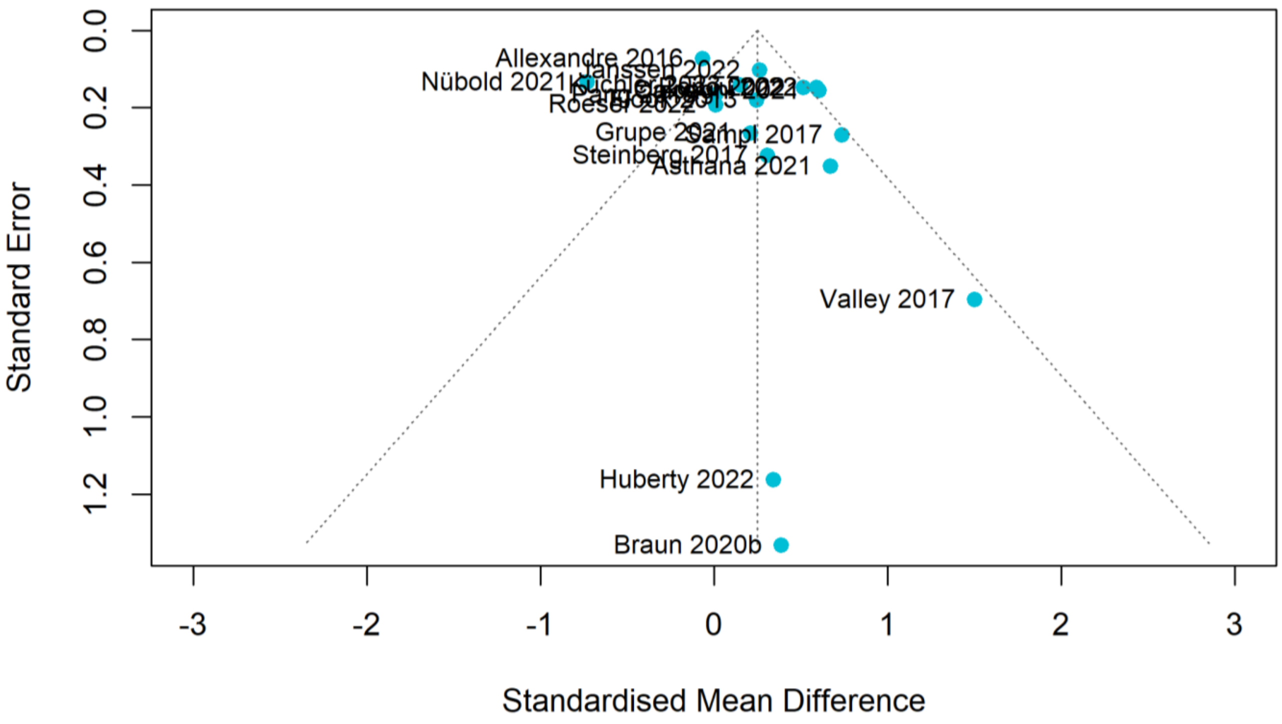

Figure S 2. Funnel plot for task performance measured up to 4 weeks post-intervention in studies using passive control groups.

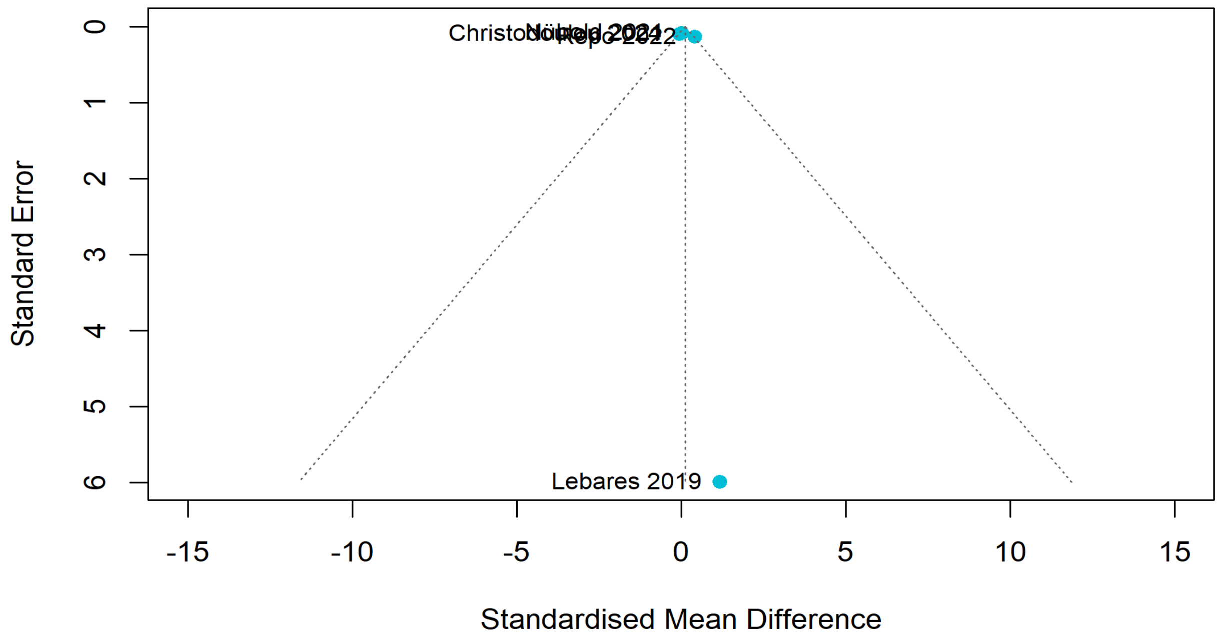

Figure S 1. Funnel plot for task performance measured up to 4 weeks post-intervention in studies using active specific control groups

Outcome measures used

Table S 4. Outcome measures used to capture work performance

Domain	Study	construct	Scale	Authors	doi	Type of scale	Direction for improvement
Adaptive performance	Aikens 2014	Resilience	Connor-Davidson Resilience Scale	Connor, K. M., & Davidson, J. R. T. (2003). Development of a new resilience scale: The Connor-Davidson Resilience Scale (CD-RISC). In Depression and Anxiety (Vol. 18, Issue 2, pp. 76–82). Wiley. https://doi.org/10.1002/da.10113	10.1002/da.10113	Ordinal	Increase
Adaptive performance	Asuero 2014	Compassion towards others	Jefferson Scale of Physician Empathy	Hojat, M., Gonnella, J. S., Nasca, T. J., Mangione, S., Vergare, M., & Magee, M. (2002). Physician Empathy: Definition, Components, Measurement, and Relationship to Gender and Specialty. In American Journal of Psychiatry (Vol. 159, Issue 9, pp. 1563–1569). American Psychiatric Association Publishing. https://doi.org/10.1176/appi.ajp.159.9.1563	10.1176/appi.ajp.159.9.1563	Ordinal	Increase
Adaptive performance	Barczak-Scarboro 2021	Resilience	Block Ego-Resilience Scale	Block, J. H., Block, J., & Collins, W. A. (Ed.) (1980). Minnesota Symposia on Child Psychology. Hillsdale, NJ: Erlbaum.	NA	Ordinal	Increase
Adaptive performance	Bonde 2022	Resilience	Brief Resilience Scale	Smith, B. W., Dalen, J., Wiggins, K., Tooley, E., Christopher, P., & Bernard, J. (2008). The brief resilience scale: Assessing the ability to bounce back. In International Journal of Behavioral Medicine (Vol. 15, Issue 3, pp. 194–200). Springer Science and Business Media LLC. https://doi.org/10.1080/10705500802222972	10.1080/10705500802222972	Ordinal	Increase
Adaptive performance	Can Gür 2020	Compassion towards others	Jefferson Empathy Scale	Yanık, A., & Saygılı, S. (2014). Validity and Reliability of the Turkish Version of Jefferson Scale of Empathy for Nursing Students. In Turkiye Klinikleri Journal of Medical Sciences (Vol. 34, Issue 1, pp. 111–119). Turkiye Klinikleri. https://doi.org/10.5336/medsci.2013-37793	10.5336/medsci.2013-37793	Ordinal	Increase
Adaptive performance	Chan 2021	Compassion towards others	Interpersonal Reactivity Index: Empathic Concern	Siu, A. M. H., & Shek, D. T. L. (2005). Validation of the Interpersonal Reactivity Index in a Chinese Context. In Research on Social Work Practice (Vol. 15, Issue 2, pp. 118–126). SAGE Publications. https://doi.org/10.1177/1049731504270384	10.1177/1049731504270384	Ordinal	Increase

Domain	Study	construct	Scale	Authors	doi	Type of scale	Direction for improvement
Adaptive performance	Christopher 2018	Resilience	Connor-Davidson Resilience Scale	Connor, K. M., & Davidson, J. R. T. (2003). Development of a new resilience scale: The Connor-Davidson Resilience Scale (CD-RISC). In Depression and Anxiety (Vol. 18, Issue 2, pp. 76–82). Wiley. https://doi.org/10.1002/da.10113	10.1002/da.10113	Ordinal	Increase
Adaptive performance	Christopher 2024	Resilience	Brief Resilience Scale	Smith, B. W., Dalen, J., Wiggins, K., Tooley, E., Christopher, P., & Bernard, J. (2008). The brief resilience scale: Assessing the ability to bounce back. In International Journal of Behavioral Medicine (Vol. 15, Issue 3, pp. 194–200). Springer Science and Business Media LLC. https://doi.org/10.1080/10705500802222972	10.1080/10705500802222972	Ordinal	Increase
Adaptive performance	dos Santos 2024	Resilience	Connor-Davidson Resilience Scale-25 Brazil	Solano, J. P. C., Bracher, E. S. B., Faisal-Cury, A., Ashmawi, H. A., Carmona, M. J. C., Lotufo Neto, F., & Vieira, J. E. (2016). Factor structure and psychometric properties of the Connor-Davidson resilience scale among Brazilian adult patients. In Sao Paulo Medical Journal (Vol. 134, Issue 5, pp. 400–406). FapUNIFESP (SciELO).	10.1590/1516-3180.2015.02290512	Ordinal	Increase
Adaptive performance	Dvoráková 2017	Compassion towards others	Compassion Scale	Pommier, E., Neff, K. D., & Tóth-Király, I. (2019). The Development and Validation of the Compassion Scale. In Assessment (Vol. 27, Issue 1, pp. 21–39). SAGE Publications. https://doi.org/10.1177/1073191119874108	10.1177/1073191119874108	Ordinal	Increase
Adaptive performance	Erogul 2014	Resilience	Resilience Scale	Wagnild, G. M., & Young, H. M. (1993). Development and psychometric evaluation of the Resilience Scale. Journal of nursing measurement, 1(2), 165–178.	NA	Ordinal	Increase
Adaptive performance	Fazia 2023	Resilience	Resilience Scale	Wagnild, G. M., & Young, H. M. (1993). Development and psychometric evaluation of the Resilience Scale. Journal of nursing measurement, 1(2), 165–178.	NA	Ordinal	Increase
Adaptive performance	Flook 2013	Understanding other groups or cultures	CLASS: Emotional support	La Paro, K. M., Pianta, R. C., & Stuhlman, M. (2004). The Classroom Assessment Scoring System: Findings from the Prekindergarten Year. In The Elementary School Journal (Vol. 104, Issue 5, pp. 409–426). University of Chicago Press. https://doi.org/10.1086/499760	10.1086/499760	Ordinal	Increase
Adaptive performance	Fraiman 2022	Compassion towards others	Interpersonal Reactivity Index's Perspective Taking	Davis, M. H. (1983). Measuring individual differences in empathy: Evidence for a multidimensional approach. In Journal of Personality and Social Psychology (Vol. 44, Issue 1, pp. 113–126). American Psychological	10.1037/0022-3514.44.1.113	Ordinal	Increase

Domain	Study	construct	Scale	Authors	doi	Type of scale	Direction for improvement
				Association (APA). https://doi.org/10.1037/0022-3514.44.1.113			
Adaptive performance	Garrote-Caparrós 2022	Compassion towards others	Empathic Understanding Scale-Patient form	El papel del terapeuta en la alianza ter-apéutica [The role of the therapist in the therapeutic alliance] [Paper presentation]. Trabajo presentado en la UNED, Guadalajara, España.	NA	Ordinal	Increase
Adaptive performance	Godara 2024	Resilience	Brief Resilience Scale	Smith, B. W., Dalen, J., Wiggins, K., Tooley, E., Christopher, P., & Bernard, J. (2008). The brief resilience scale: Assessing the ability to bounce back. In International Journal of Behavioral Medicine (Vol. 15, Issue 3, pp. 194–200). Springer Science and Business Media LLC. https://doi.org/10.1080/10705500802222972	10.1080/10705500802222972	Ordinal	Increase
Adaptive performance	Gómez-Odriozola 2019	Resilience	Brief Resilience Scale	Smith, B. W., Dalen, J., Wiggins, K., Tooley, E., Christopher, P., & Bernard, J. (2008). The brief resilience scale: Assessing the ability to bounce back. In International Journal of Behavioral Medicine (Vol. 15, Issue 3, pp. 194–200). Springer Science and Business Media LLC. https://doi.org/10.1080/10705500802222972	10.1080/10705500802222972	Ordinal	Increase
Adaptive performance	Grupe 2021	Resilience	Brief Resilience Scale	Smith, B. W., Dalen, J., Wiggins, K., Tooley, E., Christopher, P., & Bernard, J. (2008). The brief resilience scale: Assessing the ability to bounce back. In International Journal of Behavioral Medicine (Vol. 15, Issue 3, pp. 194–200). Springer Science and Business Media LLC. https://doi.org/10.1080/10705500802222972	10.1080/10705500802222972	Ordinal	Increase
Adaptive performance	Hillhouse 2023	Resilience	Connor-Davidson Resilience Scale	Connor, K. M., & Davidson, J. R. T. (2003). Development of a new resilience scale: The Connor-Davidson Resilience Scale (CD-RISC). In Depression and Anxiety (Vol. 18, Issue 2, pp. 76–82). Wiley. https://doi.org/10.1002/da.10113	10.1002/da.10113	Ordinal	Increase
Adaptive performance	Huberty 2022	Resilience	Brief Resilience Scale	Smith, B. W., Dalen, J., Wiggins, K., Tooley, E., Christopher, P., & Bernard, J. (2008). The brief resilience scale: Assessing the ability to bounce back. In International Journal of Behavioral Medicine (Vol. 15, Issue 3, pp. 194–200). Springer Science and Business Media LLC. https://doi.org/10.1080/10705500802222972	10.1080/10705500802222972	Ordinal	Increase
Adaptive performance	Jennings 2017	Understanding other groups or cultures	CLASS: Emotional support	La Paro, K. M., Pianta, R. C., & Stuhlman, M. (2004). The Classroom Assessment Scoring System: Findings from the Prekindergarten Year. In The Elementary	10.1086/499760	Ordinal	Increase

Domain	Study	construct	Scale	Authors	doi	Type of scale	Direction for improvement
				School Journal (Vol. 104, Issue 5, pp. 409–426). University of Chicago Press. https://doi.org/10.1086/499760			
Adaptive performance	Jia-Yuan 2022	Resilience	Resilience Scale for Chinese Adolescent			Ordinal	Increase
Adaptive performance	Juul 2021	Resilience	Brief Resilience Scale	Smith, B. W., Dalen, J., Wiggins, K., Tooley, E., Christopher, P., & Bernard, J. (2008). The brief resilience scale: Assessing the ability to bounce back. In International Journal of Behavioral Medicine (Vol. 15, Issue 3, pp. 194–200). Springer Science and Business Media LLC. https://doi.org/10.1080/10705500802222972	10.1080/10705500802222972	Ordinal	Increase
Adaptive performance	Klatt 2015	Resilience	Connor-Davidson Resilience Scale	Connor, K. M., & Davidson, J. R. T. (2003). Development of a new resilience scale: The Connor-Davidson Resilience Scale (CD-RISC). In Depression and Anxiety (Vol. 18, Issue 2, pp. 76–82). Wiley. https://doi.org/10.1002/da.10113	10.1002/da.10113	Ordinal	Increase
Adaptive performance	Kor 2019	Resilience	Brief Resilience Scale	Smith, B. W., Dalen, J., Wiggins, K., Tooley, E., Christopher, P., & Bernard, J. (2008). The brief resilience scale: Assessing the ability to bounce back. In International Journal of Behavioral Medicine (Vol. 15, Issue 3, pp. 194–200). Springer Science and Business Media LLC. https://doi.org/10.1080/10705500802222972	10.1080/10705500802222972	Ordinal	Increase
Adaptive performance	Kor 2020	Resilience	Brief Resilience Scale	Smith, B. W., Dalen, J., Wiggins, K., Tooley, E., Christopher, P., & Bernard, J. (2008). The brief resilience scale: Assessing the ability to bounce back. In International Journal of Behavioral Medicine (Vol. 15, Issue 3, pp. 194–200). Springer Science and Business Media LLC. https://doi.org/10.1080/10705500802222972	10.1080/10705500802222972	Ordinal	Increase
Adaptive performance	Lebares 2019	Resilience	Block Ego-Resilience Scale	Block, J. H., Block, J., & Collins, W. A. (Ed.) (1980). Minnesota Symposia on Child Psychology. Hillsdale, NJ: Erlbaum.	NA	Ordinal	Increase
Adaptive performance	Lin 2019	Resilience	Connor-Davidson Resilience Scale	Connor, K. M., & Davidson, J. R. T. (2003). Development of a new resilience scale: The Connor-Davidson Resilience Scale (CD-RISC). In Depression and Anxiety (Vol. 18, Issue 2, pp. 76–82). Wiley. https://doi.org/10.1002/da.10113	10.1002/da.10113	Ordinal	Increase
Adaptive performance	Liu 2023	Resilience	The Psychological	Zhang JH, Qiu XX, Fuch T. Study on social anxiety of nursing students coming from nonmilitary colleges	10.3760/cma.j.issn.1674-2907.2008.30.003	Ordinal	Increase

Domain	Study	construct	Scale	Authors	doi	Type of scale	Direction for improvement
			Adaptability Questionnaire	during the initial period of practicing in the military hospitals. Chin J Mod Nurs. 2008;14(30):3147-3149			
Adaptive performance	Nadler 2020	Resilience	Brief Resilience Scale	Smith, B. W., Dalen, J., Wiggins, K., Tooley, E., Christopher, P., & Bernard, J. (2008). The brief resilience scale: Assessing the ability to bounce back. In International Journal of Behavioral Medicine (Vol. 15, Issue 3, pp. 194–200). Springer Science and Business Media LLC. https://doi.org/10.1080/10705500802222972	10.1080/10705500802222972	Ordinal	Increase
Adaptive performance	Nielsen 2021	Resilience	Brief Resilience Scale	Smith, B. W., Dalen, J., Wiggins, K., Tooley, E., Christopher, P., & Bernard, J. (2008). The brief resilience scale: Assessing the ability to bounce back. In International Journal of Behavioral Medicine (Vol. 15, Issue 3, pp. 194–200). Springer Science and Business Media LLC. https://doi.org/10.1080/10705500802222972	10.1080/10705500802222972	Ordinal	Increase
Adaptive performance	Orosa-Duarte 2021	Compassion towards others	Jefferson Scale of Physician Empathy	Hojat, M., Gonnella, J. S., Nasca, T. J., Mangione, S., Vergare, M., & Magee, M. (2002). Physician Empathy: Definition, Components, Measurement, and Relationship to Gender and Specialty. In American Journal of Psychiatry (Vol. 159, Issue 9, pp. 1563–1569). American Psychiatric Association Publishing. https://doi.org/10.1176/appi.ajp.159.9.1563	10.1176/appi.ajp.159.9.1563	Ordinal	Increase
Adaptive performance	Perez-Blasco 2016	Resilience	Brief Resilient Coping Scale	Tomás, J. M., Meléndez, J. C., Sancho, P., & Mayordomo, T. (2012). Adaptation and Initial Validation of the BRCS in an Elderly Spanish Sample. In European Journal of Psychological Assessment (Vol. 28, Issue 4, pp. 283–289). Hogrefe Publishing Group. https://doi.org/10.1027/1015-5759/a000108	10.1027/1015-5759/a000108	Ordinal	Increase
Adaptive performance	Repo 2022	Resilience	Resilience Scale	Wagnild, G. M., & Young, H. M. (1993). Development and psychometric evaluation of the Resilience Scale. Journal of nursing measurement, 1(2), 165–178.	NA	Ordinal	Increase
Adaptive performance	Rodrigues de Oliveira 2021	Resilience	Connor-Davidson Resilience Scale	Connor, K. M., & Davidson, J. R. T. (2003). Development of a new resilience scale: The Connor-Davidson Resilience Scale (CD-RISC). In Depression and Anxiety (Vol. 18, Issue 2, pp. 76–82). Wiley. https://doi.org/10.1002/da.10113	10.1002/da.10113	Ordinal	Increase
Adaptive performance	Roeser 2022	Understanding other groups or cultures	CLASS: Emotional support	La Paro, K. M., Pianta, R. C., & Stuhlman, M. (2004). The Classroom Assessment Scoring System: Findings from the Prekindergarten Year. In The Elementary School Journal (Vol. 104, Issue 5, pp. 409–426).	10.1086/499760	Ordinal	Increase

Domain	Study	construct	Scale	Authors	doi	Type of scale	Direction for improvement
				University of Chicago Press. https://doi.org/10.1086/499760			
Adaptive performance	Schroeder 2018	Compassion towards others	Santa Clara Brief Compassion Scale	Hwang, J. Y., Plante, T., & Lackey, K. (2008). The Development of the Santa Clara Brief Compassion Scale: An Abbreviation of Sprecher and Fehr's Compassionate Love Scale. In Pastoral Psychology (Vol. 56, Issue 4, pp. 421–428). Springer Science and Business Media LLC. https://doi.org/10.1007/s11089-008-0117-2	10.1007/s11089-008-0117-2	Ordinal	Increase
Adaptive performance	Schroeder 2018	Resilience	Brief Resilience Scale	Smith, B. W., Dalen, J., Wiggins, K., Tooley, E., Christopher, P., & Bernard, J. (2008). The brief resilience scale: Assessing the ability to bounce back. In International Journal of Behavioral Medicine (Vol. 15, Issue 3, pp. 194–200). Springer Science and Business Media LLC. https://doi.org/10.1080/10705500802222972	10.1080/10705500802222972	Ordinal	Increase
Adaptive performance	Shapiro 1998	Compassion towards others	Empathy Construct Rating Scale	Adapted from Monica, E. L. L. (1981). Construct validity of an empathy instrument. In Research in Nursing & Health (Vol. 4, Issue 4, pp. 389–400). Wiley. https://doi.org/10.1002/nur.4770040406	10.1002/nur.4770040406	Ordinal	Increase
Adaptive performance	Shapiro 2011	Compassion towards others	Interpersonal Reactivity Index	Davis, M. H. (1983). Measuring individual differences in empathy: Evidence for a multidimensional approach. In Journal of Personality and Social Psychology (Vol. 44, Issue 1, pp. 113–126). American Psychological Association (APA). https://doi.org/10.1037/0022-3514.44.1.113	10.1037/0022-3514.44.1.113	Ordinal	Increase
Adaptive performance	Shapiro 2019	Compassion towards others	Jefferson Scale of Empathy	Hojat, M., Gonnella, J. S., Nasca, T. J., Mangione, S., Vergare, M., & Magee, M. (2002). Physician Empathy: Definition, Components, Measurement, and Relationship to Gender and Specialty. In American Journal of Psychiatry (Vol. 159, Issue 9, pp. 1563–1569). American Psychiatric Association Publishing. https://doi.org/10.1176/appi.ajp.159.9.1563	10.1176/appi.ajp.159.9.1563	Ordinal	Increase
Adaptive performance	Strauss 2021	Compassion towards others	Sussex-Oxford Compassion Scale Other	Gu, J., Baer, R., Cavanagh, K., Kuyken, W., & Strauss, C. (2019). Development and Psychometric Properties of the Sussex-Oxford Compassion Scales (SOCS). In Assessment (Vol. 27, Issue 1, pp. 3–20). SAGE Publications. https://doi.org/10.1177/1073191119860911	10.1177/1073191119860911	Ordinal	Increase
Adaptive performance	Taylor 2016	Compassion towards others	4-item Santa Clara Brief	Adapted from Hwang, J. Y., Plante, T., & Lackey, K. (2008). The Development of the Santa Clara Brief	10.1007/s11089-008-0117-2	Ordinal	Increase

Domain	Study	construct	Scale	Authors	doi	Type of scale	Direction for improvement
			Compassion Scale	Compassion Scale: An Abbreviation of Sprecher and Fehr's Compassionate Love Scale. In Pastoral Psychology (Vol. 56, Issue 4, pp. 421–428). Springer Science and Business Media LLC. https://doi.org/10.1007/s11089-008-0117-2			
Adaptive performance	Taylor 2022	Compassion towards others	Compassionate Love Scale	Sprecher S, Fehr B. Compassionate love for close others and humanity. J Social Personal Relationship 2016 Jun		Ordinal	Increase
Adaptive performance	Pérula-de Torres 2021	Compassion towards others	Jefferson Medical Empathy Scale			Ordinal	Increase
Adaptive performance	van Dijk 2017	Compassion towards others	Jefferson Scale of Physician Empathy	Adapted from Hojat, M., Gonnella, J. S., Nasca, T. J., Mangione, S., Vergare, M., & Magee, M. (2002). Physician Empathy: Definition, Components, Measurement, and Relationship to Gender and Specialty. In American Journal of Psychiatry (Vol. 159, Issue 9, pp. 1563–1569). American Psychiatric Association Publishing. https://doi.org/10.1176/appi.ajp.159.9.1563	10.1176/appi.ajp.159.9.1563	Ordinal	Increase
Adaptive performance	Verweij 2018	Compassion towards others	Jefferson Scale of Physician Empathy	Hojat, M., Gonnella, J. S., Nasca, T. J., Mangione, S., Vergare, M., & Magee, M. (2002). Physician Empathy: Definition, Components, Measurement, and Relationship to Gender and Specialty. In American Journal of Psychiatry (Vol. 159, Issue 9, pp. 1563–1569). American Psychiatric Association Publishing. https://doi.org/10.1176/appi.ajp.159.9.1563	10.1176/appi.ajp.159.9.1563	Ordinal	Increase
Adaptive performance	Wang 2023	Resilience	Connor-Davidson Resilience Scale	Connor, K. M., & Davidson, J. R. T. (2003). Development of a new resilience scale: The Connor-Davidson Resilience Scale (CD-RISC). In Depression and Anxiety (Vol. 18, Issue 2, pp. 76–82). Wiley. https://doi.org/10.1002/da.10113	10.1002/da.10113	Ordinal	Increase
Adaptive performance	Watson-Singleton 2024	Resilience	Brief Resilience Scale	Smith, B. W., Dalen, J., Wiggins, K., Tooley, E., Christopher, P., & Bernard, J. (2008). The brief resilience scale: Assessing the ability to bounce back. In International Journal of Behavioral Medicine (Vol. 15, Issue 3, pp. 194–200). Springer Science and Business Media LLC. https://doi.org/10.1080/10705500802222972	10.1080/10705500802222972	Ordinal	Increase
Adaptive performance	Wilson 2022	Resilience	Connor-Davidson Resilience Scale	Connor, K. M., & Davidson, J. R. T. (2003). Development of a new resilience scale: The Connor-Davidson Resilience Scale (CD-RISC). In Depression	10.1002/da.10113	Ordinal	Increase

Domain	Study	construct	Scale	Authors	doi	Type of scale	Direction for improvement
				and Anxiety (Vol. 18, Issue 2, pp. 76–82). Wiley. https://doi.org/10.1002/da.10113			
Adaptive performance	AlQarni 2023	Resilience	Connor-Davidson Resilience Scale-10	Not reported		Ordinal	Increase
Contextual performance	Aikens 2014	Engagement	Shirom Vigor Scale	Shirom A. (2004). Feeling vigorous at work? The construct of vigor and the study of positive affect in organizations. In D.C. Ganster P.L. Perrewe (Eds.), Research in organizational stress and well-being. 135-164. Greenwich, CN: JAI Press.	NA	Ordinal	Increase
Contextual performance	Baumgartner 2021	Engagement	Enrolment in the next semester classes	Bespoke scale	NA	Nominal	Increase
Contextual performance	Bellosta-Batalla 2021	Creativity	Creative Imagination Test for Adults: Fluency subscale	Artola, T., Ancillo, I., Barraca, J. & Mosteiro, P. (2010). PIC-A. Prueba de Imaginación Creativa para Adultos [Creative Imagination Test for Adults]. Madrid: TEA Edicione	NA	Ordinal	Increase
Contextual performance	Benn 2012	Efficacy	Teaching self-efficacy	Adapted from Midgley, C., Maehr, M. L., Hruda, L. Z., Anderman, E., Anderman, L., Freeman, K. E., . . . Urdan, T. (2000). Manual for the Patterns of Adaptive Learning Scales (PALS). Ann Arbor, MI: University of Michigan.	NA	Ordinal	Increase
Contextual performance	Braun 2020a	Interpersonal relations	Tendency to forgive scale	Brown, R. P. (2003). Measuring Individual Differences in the Tendency to Forgive: Construct Validity and Links with Depression. In Personality and Social Psychology Bulletin (Vol. 29, Issue 6, pp. 759–771). SAGE Publications. https://doi.org/10.1177/0146167203029006008	10.1177/0146167203029006008	Ordinal	Increase
Contextual performance	Brown 2016	Interpersonal relations	Mutuality Scale of the Family Care Inventory	Archbold, P. G., Stewart, B. J., Greenlick, M. R., & Harvath, T. (1990). Mutuality and preparedness as predictors of caregiver role strain. In Research in Nursing & Health (Vol. 13, Issue 6, pp. 375–384). Wiley. https://doi.org/10.1002/nur.4770130605	10.1002/nur.4770130605	Ordinal	Increase
Contextual performance	Calcagni 2021	Engagement	Utrecht Work Engagement Scale-9	Schaufeli, W. B., & Bakker, A. B. (2004). Job demands, job resources, and their relationship with burnout and engagement: a multi-sample study. In Journal of Organizational Behavior (Vol. 25, Issue 3, pp. 293–315). Wiley. https://doi.org/10.1002/job.248	10.1002/job.248	Ordinal	Increase

Domain	Study	construct	Scale	Authors	doi	Type of scale	Direction for improvement
Contextual performance	de Carvalho 2021	Efficacy	Teachers' Sense of Efficacy Questionnaire Long Form	Tschannen-Moran, M., & Woolfolk Hoy, A. (2001). Teacher efficacy: Capturing and elusive construct. Teaching and Teacher Education, 17, 783-805	NA	Ordinal	Increase
Contextual performance	de Jong 2013	Efficacy	Job Seeking Self-Efficacy Scale	Barlow, J., Wright, C., & Cullen, L. (2002). A job-seeking self-efficacy scale for people with physical disabilities: Preliminary development and psychometric testing. In British Journal of Guidance & Counselling (Vol. 30, Issue 1, pp. 37–53). Informa UK Limited. https://doi.org/10.1080/030698880220106500	10.1080/030698880220106500	Ordinal	Increase
Contextual performance	Desai 2024	Engagement	Utrecht Work Engagement Scale-17	Schaufeli, W. B., & Bakker, A. B. (2004). Job demands, job resources, and their relationship with burnout and engagement: a multi-sample study. In Journal of Organizational Behavior (Vol. 25, Issue 3, pp. 293–315). https://doi.org/10.1002/job.248	10.1002/job.248	Ordinal	Increase
Contextual performance	Erden 2023	Motivation	Nurses Job Motivation Scale	Adapted from Engin, E., & Çam, M. O. (2016). The nurses job motivation scale: validity and reliability. Journal of Ege University Faculty of Nursing, 32(3), 1-13.		Ordinal	Increase
Contextual performance	Flook 2013	Inititative and proactivity	CLASS: Instructional Support	La Paro, K. M., Pianta, R. C., & Stuhlman, M. (2004). The Classroom Assessment Scoring System: Findings from the Prekindergarten Year. In The Elementary School Journal (Vol. 104, Issue 5, pp. 409–426). University of Chicago Press. https://doi.org/10.1086/499760	10.1086/499760	Ordinal	Increase
Contextual performance	Garrote-Caparrós 2022	Interpersonal relations	Working Alliance Theory of Change Inventory	Corbella and Botella 2004. Psychometric properties of the Spanish version of the Working Alliance Theory of Change Inventory (WATOCI). Psicothema, 16, 702–705. https://reunido.uniovi.es/index.php/PST/article/view/8178	NA	Ordinal	Increase
Contextual performance	Hwang 2019	Efficacy	Teachers' Sense of Efficacy Questionnaire Short Form	Tschannen-Moran, M., & Woolfolk Hoy, A. (2001). Teacher efficacy: Capturing and elusive construct. Teaching and Teacher Education, 17, 783-805	NA	Ordinal	Increase
Contextual performance	Janssen 2023	Contextual performance	Individual Work Performance Questionnaire (IWPQ) -	Koopmans, L., Coffeng, J. K., Bernaards, C. M., Boot, C. R., Hildebrandt, V. H., de Vet, H. C., & van der Beek, A. J. (2014). Responsiveness of the individual work	10.1186/1471-2458-14-513	Ordinal	Increase

Domain	Study	construct	Scale	Authors	doi	Type of scale	Direction for improvement
			subscale contextual performance	performance questionnaire. In BMC Public Health (Vol. 14, Issue 1). Springer Science and Business Media LLC			
Contextual performance	Jennings 2013	Efficacy	Teachers' Sense of Efficacy Questionnaire Long Form	Tschannen-Moran, M., & Woolfolk Hoy, A. (2001). Teacher efficacy: Capturing and elusive construct. Teaching and Teacher Education, 17, 783-805	NA	Ordinal	Increase
Contextual performance	Jennings 2017	Inititative and proactivity	CLASS: Instructional Support	La Paro, K. M., Pianta, R. C., & Stuhlman, M. (2004). The Classroom Assessment Scoring System: Findings from the Prekindergarten Year. In The Elementary School Journal (Vol. 104, Issue 5, pp. 409–426). University of Chicago Press. https://doi.org/10.1086/499760	10.1086/499760	Ordinal	Increase
Contextual performance	Karing 2021	Efficacy	General Self-Efficacy Scale	Schwarzer & Jerusalem, 1999		Ordinal	Increase
Contextual performance	Klatt 2015	Engagement	Utrecht Work Engagement Scale-9	Schaufeli, W. B., & Bakker, A. B. (2004). Job demands, job resources, and their relationship with burnout and engagement: a multi-sample study. In Journal of Organizational Behavior (Vol. 25, Issue 3, pp. 293–315). Wiley. https://doi.org/10.1002/job.248	10.1002/job.248	Ordinal	Increase
Contextual performance	Klatt 2017	Engagement	Utrecht Work Engagement Scale-9	Schaufeli, W. B., & Bakker, A. B. (2004). Job demands, job resources, and their relationship with burnout and engagement: a multi-sample study. In Journal of Organizational Behavior (Vol. 25, Issue 3, pp. 293–315). Wiley. https://doi.org/10.1002/job.248	10.1002/job.248	Ordinal	Increase
Contextual performance	Küchler 2023	Efficacy	General Self-Efficacy Scale	Schwarzer & Jerusalem, 1999		Ordinal	Increase
Contextual performance	Kuyken 2022	Efficacy	Teachers' Sense of Efficacy Questionnaire Short Form	Tschannen-Moran, M., & Woolfolk Hoy, A. (2001). Teacher efficacy: Capturing and elusive construct. Teaching and Teacher Education, 17, 783-805	NA	Ordinal	Increase
Contextual performance	Lensen 2024	Efficacy	Teachers' Sense of Efficacy Questionnaire Short Form	Hoogendijk, C., Tick, N. T., Hofman, W. H. A., Holland, J. G., Severiens, S. E., Vuijk, P., & van Veen, A. F. D. (2018). Direct and indirect effects of Key2Teach on teachers' sense of self-efficacy and emotional exhaustion, a randomized controlled trial. In Teaching and Teacher Education (Vol. 76, pp. 1–13).	10.1016/j.tate.2018.07.014	Ordinal	Increase
Contextual performance	Modrego-Alarcón 2021	Engagement	The Utrecht Work Engagement			Ordinal	Increase

Domain	Study	construct	Scale	Authors	doi	Type of scale	Direction for improvement
			Survey Scale-Students				
Contextual performance	Phang 2015	Efficacy	General Self-Efficacy	Schwarzer, R., & Jerusalem, M. (1995). General Self-Efficacy Scale [Data set]. In PsycTESTS Dataset. American Psychological Association (APA). https://doi.org/10.1037/t00393-000	10.1037/t00393-000	Ordinal	Increase
Contextual performance	Pipe 2009	Efficacy	Caring Efficacy Scale	Adapted from Coates C. J. (1997). The Caring Efficacy Scale: nurses' self-reports of caring in practice settings. Advanced practice nursing quarterly, 3(1), 53–59.	NA	Ordinal	Increase
Contextual performance	Rich 2021	Engagement	Job Engagement Scale	Rich, B. L., Lepine, J. A., & Crawford, E. R. (2010). Job Engagement: Antecedents and Effects on Job Performance. In Academy of Management Journal (Vol. 53, Issue 3, pp. 617–635). Academy of Management. https://doi.org/10.5465/amj.2010.51468988	10.5465/amj.2010.51468988	Ordinal	Increase
Contextual performance	Sampl 2017	Efficacy	Self-Efficacy Scale	Pintrich, P. R., & De Groot, E. V. (1990). Motivational and self-regulated learning components of classroom academic performance. In Journal of Educational Psychology (Vol. 82, Issue 1, pp. 33–40). American Psychological Association (APA). https://doi.org/10.1037/0022-0663.82.1.33	10.1037/0022-0663.82.1.33	Ordinal	Decrease
Contextual performance	Sampl 2017	Inititative and proactivity	Revised Self-Leadership Questionnaire-Deutsch	Andreßen, P., & Konradt, U. (2007). Messung von Selbstführung: Psychometrische Überprüfung der deutschsprachigen Version des Revised Self-Leadership Questionnaire. In Zeitschrift für Personalpsychologie (Vol. 6, Issue 3, pp. 117–128). Hogrefe Publishing Group. https://doi.org/10.1026/1617-6391.6.3.117	10.1026/1617-6391.6.3.117	Ordinal	Increase
Contextual performance	Steinberg 2017	Engagement	Utrecht Work Engagement Scale-9	Schaufeli, W. B., & Bakker, A. B. (2004). Job demands, job resources, and their relationship with burnout and engagement: a multi-sample study. In Journal of Organizational Behavior (Vol. 25, Issue 3, pp. 293–315). Wiley. https://doi.org/10.1002/job.248	10.1002/job.248	Ordinal	Increase
Contextual performance	Valley 2017	Effort	Workplace safety performance: Safety participation	Adapted from Neal, A., Griffin, M. A., & Hart, P. M. (2000). The impact of organizational climate on safety climate and individual behavior. In Safety Science (Vol. 34, Issues 1–3, pp. 99–109). Elsevier BV. https://doi.org/10.1016/s0925-7535(00)00008-4	10.1016/S0925-7535(00)00008-4	Ordinal	Increase
Contextual performance	van Berkel 2014, van Dongen 2016	Engagement	Utrecht Work Engagement Scale-17	Schaufeli, W. B., & Bakker, A. B. (2004). Job demands, job resources, and their relationship with burnout and engagement: a multi-sample study. In Journal of	10.1002/job.248	Ordinal	Increase

Domain	Study	construct	Scale	Authors	doi	Type of scale	Direction for improvement
				Organizational Behavior (Vol. 25, Issue 3, pp. 293–315). https://doi.org/10.1002/job.248			
Counterproductive work behaviour	Balci 2023	Presenteeism	Presenteeism Scale for Students	Matsushita, M., Adachi, H., Arakida, M., Namura, I., Takahashi, Y., Miyata, M., Kumano-go, T., Yamamura, S., Shigedo, Y., Suganuma, N., Mikami, A., Moriyama, T., & Sugita, Y. (2010). Presenteeism in college students: reliability and validity of the Presenteeism Scale for Students. In Quality of Life Research (Vol. 20, Issue 3, pp. 439–446).	10.1007/s11136-010-9763-9	Ordinal	Decrease
Counterproductive work behaviour	Bartlett 2017	Absenteeism and presenteeism	Productivity loss in days	Bespoke scale	NA	Ratio	Decrease
Counterproductive work behaviour	Can Gür 2020	Discrimination	Age Discrimination Attitude Scale	Yılmaz D. V., and Terzioglu, F. (2011). Development and psychometric evaluation of ageism attitude scale among the university students. Turk Geriatri Dergisi 14(3), 259-268	NA	Ordinal	Decrease
Counterproductive work behaviour	Choi 2022	Accuracy	Error Orientation and Motivation Scale	Schell, K. L. (2012). The Error-Oriented Motivation Scale: An examination of structural and convergent validity. Personality and Individual Differ- ences, 52(3), 352–356	10.1016/j.paid.2011.10.035	Ordinal	Decrease
Counterproductive work behaviour	Hunsinger 2019	Discrimination	Shooter Bias Task	Adapted from Correll, J., Park, B., Judd, C. M., & Wittenbrink, B. (2002). The police officer's dilemma: Using ethnicity to disambiguate potentially threatening individuals. In Journal of Personality and Social Psychology (Vol. 83, Issue 6, pp. 1314–1329). American Psychological Association (APA). https://doi.org/10.1037/0022-3514.83.6.1314	10.1037/0022-3514.83.6.1314	Ordinal	Increase
Counterproductive work behaviour	Janssen 2023	Counterproducive work behaviour	Individual Work Performance Questionnaire (IWPQ) - subscale counterproductive work behaviour	Koopmans, L., Coffeng, J. K., Bernaards, C. M., Boot, C. R., Hildebrandt, V. H., de Vet, H. C., & van der Beek, A. J. (2014). Responsiveness of the individual work performance questionnaire. In BMC Public Health (Vol. 14, Issue 1). Springer Science and Business Media LLC	10.1186/1471-2458-14-513	Ordinal	Decrease
Counterproductive work behaviour	Küchler 2023	Absenteeism	Modified Standford Presenteeism Scale	Matsushita, M.; Adachi, H.; Arakida, M.; Namura, I.; Takahashi, Y.; Miyata, M.; Kumano-go, T.; Yamamura, S.; Shigedo, Y.; Suganuma, N.; et al. Presenteeism in college students: Reliability and validity of the presenteeism scale for		Ratio	Decrease

Domain	Study	construct	Scale	Authors	doi	Type of scale	Direction for improvement
				students. Qual. Life Res. 2011, 20, 439–446			
Counterproductive work behaviour	Liu 2022	Disregard for safety	Adverse events self-report	Bespoke scale	NA	Ordinal	Decrease
Counterproductive work behaviour	Ogino 2024	Presenteeism	Single-item presenteeism adapted from the standardized World Health Organization Health and Work Performance Questionnaire	Adapted from Kessler, R. C., Barber, C., Beck, A., Berglund, P., Cleary, P. D., McKenas, D., Pronk, N., Simon, G., Stang, P., Ustun, T. B., & Wang, P. (2003). The World Health Organization Health and Work Performance Questionnaire (HPQ). In Journal of Occupational and Environmental Medicine (Vol. 45, Issue 2, pp. 156–174).	10.1097/01.jom.0000052967.43131.51	Ratio	Increase
Counterproductive work behaviour	Rad 2023	Off-task behaviour	The multifaceted measure of academic procrastination	Haghbin, M. (2015). Conceptualization and operationalization of delay: Development and validation of the multifaceted measure of academic procrastination and the delay questionnaire. Carleton University. https://doi.org/10.22215/etd/2015-11051.		Ordinal	Decrease
Counterproductive work behaviour	Roeser 2013	Absenteeism	Teacher absences from work	Bespoke scale	NA	Ratio	Decrease
Counterproductive work behaviour	Steinberg 2017	Absenteeism and presenteeism	Days on sick leave or reduced ability to work	NA	NA	Ratio	Decrease
Counterproductive work behaviour	Strauss 2021	Presenteeism	Institute for Medical Technology Assessment Productivity Cost Questionnaire	Adapted from Bouwmans, C., Krol, M., Severens, H., Koopmanschap, M., Brouwer, W., & Roijen, L. H. (2015). The iMTA Productivity Cost Questionnaire. In Value in Health (Vol. 18, Issue 6, pp. 753–758). Elsevier BV. https://doi.org/10.1016/j.jval.2015.05.009	10.1016/j.jval.2015.05.009	Ordinal	Decrease
Counterproductive work behaviour	Taylor 2022	Absenteeism	Days on sick leave	30;22(5):629-651.	NA	Ratio	Decrease
Counterproductive work behaviour	van Berkel 2014, van Dongen 2016	Absenteeism	Absenteeism	Bespoke scale	NA	Ratio	Decrease
Task performance	Daigle 2018	Doing tasks incorrectly	Nursing Errors Rating Scale	Bespoke scale	NA	Ordinal	Increase

Domain	Study	construct	Scale	Authors	doi	Type of scale	Direction for improvement
Task performance	Nassif 2023 study 1	Accuracy	Weapons qualification			Ordinal	Increase
Task performance	Nassif 2023 study 2	Accuracy	Weapons qualification			Ordinal	Increase
Task performance	Verweij 2018	Doing tasks incorrectly	Medical errors	Prins, J. T., van der Heijden, F. M. M. A., Hoekstra-Weebers, J. E. H. M., Bakker, A. B., van de Wiel, H. B. M., Jacobs, B., & Gazendam-Donofrio, S. M. (2009). Burnout, engagement and resident physicians' self-reported errors. In Psychology, Health & Medicine (Vol. 14, Issue 6, pp. 654–666). Informa UK Limited. https://doi.org/10.1080/13548500903311554	10.1080/13548500903311554	Ordinal	Decrease
Task performance	Asthana 2021	Decision-making	A case study exercise	Bespoke scale	NA	Ordinal	Increase
Task performance	Hillhouse 2023	Decision-making	Sunk Cost Inventory	Strough, J., Mehta, C. M., McFall, J. P., & Schuller, K. L. (2008). Are older adults less subject to the sunk-cost fallacy than younger adults? Psychological Science, 19(7), 650–652.	10. 1111/j.1467-9280.2008.02138.x	Ordinal	Decrease
Task performance	Liu 2022	Work quality	Hospital Survey on Patient Safety Culture			Ordinal	Increase
Task performance	Allexandre 2016	Productivity	Bespoke score	Bespoke scale	NA	Ordinal	Decrease
Task performance	Braun 2020b	Productivity	Work Productivity and Activity Impairment Questionnaire plus Classroom Impairment Questions	Reilly, M. C., Zbrozek, A. S., & Dukes, E. M. (1993). The Validity and Reproducibility of a Work Productivity and Activity Impairment Instrument. In PharmacoEconomics (Vol. 4, Issue 5, pp. 353–365). Springer Science and Business Media LLC. https://doi.org/10.2165/00019053-199304050-00006	10.2165/00019053-199304050-00006	Ordinal	Decrease
Task performance	Calcagni 2021	Productivity	Performance: In-role Work Performance	Goodman, S. A., and Svyantek, D. J. (1999). Person-Organization Fit and Contextual Performance: do Shared Values Matter. J. Vocat. Behav. 55, 254–275.	10.1006/jvbe.1998.1682	Ordinal	Increase
Task performance	Christodoulou 2024	Productivity	Work Limitations Questionnaire-Short Form	Lerner, D., Amick, B. C., III, Rogers, W. H., Malspeis, S., Bungay, K., & Cynn, D. (2001). The Work Limitations Questionnaire. Medical Care, 39(1), 72-85.		Ordinal	Decrease
Task performance	Garrote-Caparrós 2022	Productivity	Brief Symptom Inventory	Derogatis LR. BSI 18, Brief Symptom Inventory 18: Administration, scoring and Procedure Manual. Minneapolis, MN: NCS Pearson, Incorporated; 2001		Ordinal	Decrease

Domain	Study	construct	Scale	Authors	doi	Type of scale	Direction for improvement
Task performance	Grupe 2021	Productivity	Work Limitations Questionnaire	Lerner, D., Amick, B. C., III, Rogers, W. H., Malspeis, S., Bungay, K., & Cynn, D. (2001). The Work Limitations Questionnaire. Medical Care, 39(1), 72-85.		Ordinal	Decrease
Task performance	Huberty 2022	Productivity	Work Productivity and Activity Impairment Questionnaire - General Health measure (WPAI general health): Sub-scale "work impairment"	Reilly, M. C., Zbrozek, A. S., & Dukes, E. M. (1993). The Validity and Reproducibility of a Work Productivity and Activity Impairment Instrument. In PharmacoEconomics (Vol. 4, Issue 5, pp. 353–365). Springer Science and Business Media LLC. https://doi.org/10.2165/00019053-199304050-00006	10.2165/00019053-199304050-00006	Ordinal	Decrease
Task performance	Küchler 2023	Productivity	Presenteeism Scale for Students - work output subscale	Matsushita, M.; Adachi, H.; Arakida, M.; Namura, I.; Takahashi, Y.; Miyata, M.; Kumano-go, T.; Yamamura, S.; Shigedo, Y.; Suganuma, N.; et al. Presenteeism in college students: Reliability and validity of the presenteeism scale for students. Qual. Life Res. 2011, 20, 439–446		Ordinal	Increase
Task performance	Nübold 2021	Productivity	Three items from Williams and Anderson (1991) that were also adjusted to the day (also see Nohe et al., 2014).	Williams, L. J., & Anderson, S. E. (1991). Job satisfaction and organizational commitment as predictors of organi-zational citizenship and in-role behaviors. Journal of Management, 17(3), 601–617.	NA	Ordinal	Increase
Task performance	Repo 2022	Productivity	Functional ability at work	Tuomi, K. (2001). Promotion of work ability, the quality of work and retirement. In Occupational Medicine (Vol. 51, Issue 5, pp. 318–324). Oxford University Press	10.1093/occmed/51.5.318	Ordinal	Increase
Task performance	van Berkel 2014, van Dongen 2016	Productivity	Work Ability Index	Adapted from Tuomi K, Ilmarinen J, Jahkola A, Katajarinne L, Tulkki A. (1998) Work Ability Index. Helsinki, Finland: Finish Institute of Occupational Health.	NA	Ordinal	Increase
Task performance	Vainre 2024	Productivity	Work Role Functioning Questionnaire	Abma, F. I., Bültmann, U., Amick III, B. C., Arends, I., Dorland, H. F., Flach, P. A., van der Klink, J. J. L., van de Ven, H. A., & Bjørner, J. B. (2017). The Work Role Functioning Questionnaire v2.0 Showed Consistent Factor Structure Across Six Working Samples. In	10.1007/s10926-017-9722-1	Ordinal	Increase

Domain	Study	construct	Scale	Authors	doi	Type of scale	Direction for improvement
				Journal of Occupational Rehabilitation (Vol. 28, Issue 3, pp. 465–474).			
Task performance	Glass 2019	Skills or knowledge	Coach Rating Form	Bespoke scale	NA	Ordinal	Increase
Task performance	Janssen 2022	Task performance	Individual Work Performance Questionnaire	Koopmans, L., Coffeng, J. K., Bernaards, C. M., Boot, C. R., Hildebrandt, V. H., de Vet, H. C., & van der Beek, A. J. (2014). Responsiveness of the individual work performance questionnaire. In BMC Public Health (Vol. 14, Issue 1). Springer Science and Business Media LLC	10.1186/1471-2458-14-513	Ordinal	Increase
Task performance	Augustus 2024	Work quality	Multidimensional Thriving at Work Scale	Porath, C., Spreitzer, G., Gibson, C., & Garnett, F. G. (2011). Thriving at work: Toward its measurement, construct validation, and theoretical refinement. In Journal of Organizational Behavior (Vol. 33, Issue 2, pp. 250–275).	10.1002/job.756	Ordinal	Increase
Task performance	Baumgartner 2021	Work quality	Grade point Average	Bespoke scale	NA	Ratio	Increase
Task performance	Flook 2013	Work quality	CLASS: Classroom organisation	La Paro, K. M., Pianta, R. C., & Stuhlman, M. (2004). The Classroom Assessment Scoring System: Findings from the Prekindergarten Year. In The Elementary School Journal (Vol. 104, Issue 5, pp. 409–426). University of Chicago Press. https://doi.org/10.1086/499760	10.1086/499760	Ordinal	Increase
Task performance	Galante 2018	Work quality	Examination results	Bespoke scale	NA	Nominal	Decrease
Task performance	Jennings 2017	Work quality	CLASS: Classroom organisation	La Paro, K. M., Pianta, R. C., & Stuhlman, M. (2004). The Classroom Assessment Scoring System: Findings from the Prekindergarten Year. In The Elementary School Journal (Vol. 104, Issue 5, pp. 409–426). University of Chicago Press. https://doi.org/10.1086/499760	10.1086/499760	Ordinal	Increase
Task performance	Lebares 2019	Work quality	Peg transfer task	Peters JH, Fried GM, Swanstrom LL, et al; SAGES FLS Committee. Development and validation of a comprehensive program of education and assessment of the basic fundamentals of laparoscopic surgery. Surgery. 2004;135(1):21-27.	10.1016/S0039-6060(03)00156-9	Ratio	Decrease
Task performance	Pang 2019	Work quality	Task Performance Questionnaire	Adapted from Williams, L. J., & Anderson, S. E. (1991). Job Satisfaction and Organizational Commitment as Predictors of Organizational Citizenship and In-Role	10.1177/014920639101700305	Ordinal	Increase

Domain	Study	construct	Scale	Authors	doi	Type of scale	Direction for improvement
				Behaviors. In Journal of Management (Vol. 17, Issue 3, pp. 601–617). SAGE Publications. https://doi.org/10.1177/014920639101700305			
Task performance	Roeser 2022	Work quality	CLASS: Classroom organisation	La Paro, K. M., Pianta, R. C., & Stuhlman, M. (2004). The Classroom Assessment Scoring System: Findings from the Prekindergarten Year. In The Elementary School Journal (Vol. 104, Issue 5, pp. 409–426). University of Chicago Press. https://doi.org/10.1086/499760	10.1086/499760	Ordinal	Increase
Task performance	Sampl 2017	Work quality	Grade point Average	Bespoke scale	NA	Ratio	Decrease
Task performance	Schroeder 2018	Work quality	Consumer Assessment of Healthcare Providers and Systems–Clinician and Group Adult Doctor Communication Composite	Adapted from Dyer, N., Sorra, J. S., Smith, S. A., Cleary, P. D., & Hays, R. D. (2012). Psychometric Properties of the Consumer Assessment of Healthcare Providers and Systems (CAHPS®) Clinician and Group Adult Visit Survey. In Medical Care (Vol. 50, pp. S28–S34). Ovid Technologies (Wolters Kluwer Health). https://doi.org/10.1097/mlr.0b013e31826cbc0d	10.1097/MLR.0b013e31826cbc0d	Ordinal	Increase
Task performance	Steinberg 2017	Work quality	Decreased ability to work	Bespoke scale	NA	Ratio	Decrease
Task performance	Takhdat 2024	Work quality	Team Emergency Assessment Measure	Maignan, M., Koch, F.-X., Chaix, J., Phellouzat, P., Binauld, G., Collomb Muret, R., Cooper, S. J., Labarère, J., Danel, V., Viglino, D., & Debaty, G. (2016). Team Emergency Assessment Measure (TEAM) for the assessment of non-technical skills during resuscitation: Validation of the French version. In Resuscitation (Vol. 101, pp. 115–120).	10.1016/j.resuscitation.2015.11.024	Ordinal	Increase
Task performance	Valley 2017	Work quality	Workplace cognitive failure	Adapted from Wallace, J. Craig., & Chen, G. (2005). Development and validation of a work-specific measure of cognitive failure: Implications for occupational safety. In Journal of Occupational and Organizational Psychology (Vol. 78, Issue 4, pp. 615–632). Wiley. https://doi.org/10.1348/096317905x37442	10.1348/096317905X37442	Ordinal	Decrease

Studies not meta-analysed due to lack of reports on outcomes

Table S 5. Studies not meta-analysed due to lack of reports on outcomes

Study	Domain	Timepoint	Control group	Reason for exclusion
Nassif 2023 study 1	Task performance	Up to 4 weeks post-intervention	Passive	Can't extract outcomes as only 3-way ANOVA results presented
Nassif 2023 study 2	Task performance	Up to 4 weeks post-intervention	Passive	Can't extract outcomes as only 3-way ANOVA results presented
Daigle 2018	Task performance	5-24 weeks post-intervention	Passive	Waitlist group data not collected
Glass 2019	Task performance	5-24 weeks post-intervention	Passive	Waitlist group data not reported
Baumgartner 2021	Task performance	5-24 weeks post-intervention	Active	Only partial eta reported, no info to convert to SMD
Baumgartner 2021	Task performance	5-24 weeks post-intervention	Passive	Only partial eta reported, no info to convert to SMD
van Berkel 2014, van Dongen 2016	Task performance	5-24 weeks post-intervention	Active	Data not reported
Nassif 2023 study 1	Task performance	5-24 weeks post-intervention	Passive	Only 3-way ANOVA reported
Nassif 2023 study 2	Task performance	5-24 weeks post-intervention	Passive	Only 3-way ANOVA reported
Glass 2019	Task performance		Passive	Data not reported
Steinberg 2017	Contextual performance	Up to 4 weeks post-intervention	Passive	No between-group comparison
Klatt 2015	Contextual performance	Up to 4 weeks post-intervention	Passive	No between-group comparison
Aikens 2014	Contextual performance	5-24 weeks post-intervention	Passive	Waitlist group data not collected
Klatt 2017	Contextual performance	5-24 weeks post-intervention	Passive	Waitlist group data not collected
Pipe 2009	Contextual performance		Placebo	Data not collected; study cut short
Valley 2017	Contextual performance		Passive	Waitlist group data not collected

Study	Domain	Timepoint	Control group	Reason for exclusion
Klatt 2015	Adaptive performance	Up to 4 weeks post-intervention	Passive	No between-group comparison reported
Barczak-Scarboro 2021	Adaptive performance	Up to 4 weeks post-intervention	Active	Data per arm not reported, data available at https://osf.io/d9qux/; data access requested but not granted
Shapiro 2019	Adaptive performance	Up to 4 weeks post-intervention	Active	Data not reported, data requested from author; received but no arm variable present in dataset, so cannot compute effect sizes. Requested for arm variable, response not received
Choi 2024	Adaptive performance	Up to 4 weeks post-intervention	Placebo	Data not reported, data requested from author but no response
Choi 2024	Adaptive performance	Up to 4 weeks post-intervention	Passive	Data not reported, data requested from author but no response
dos Santos 2024	Adaptive performance	Up to 4 weeks post-intervention	Passive	Only regression coefficients available, but no SD/SE so cannot impute effect size
Christopher 2024	Adaptive performance	Up to 4 weeks post-intervention	Placebo	Does not report SD or SE, so cannot compute nor impute effect size
Christopher 2024	Adaptive performance	Up to 4 weeks post-intervention	Passive	Does not report SD or SE, so cannot compute nor impute effect size
Chan 2021	Adaptive performance	5-24 weeks post-intervention	Passive	SDs not reported
Taylor 2016	Adaptive performance	5-24 weeks post-intervention	Passive	No data reported
Barczak-Scarboro 2021	Adaptive performance	5-24 weeks post-intervention	Active	Data per arm not reported, data available at https://osf.io/d9qux/; data access requested but not granted
Christopher 2024	Adaptive performance	5-24 weeks post-intervention	Placebo	SD/SE not reported
Christopher 2024	Adaptive performance	5-24 weeks post-intervention	Passive	SD/SE not reported
Fraiman 2022	Adaptive performance		Placebo	Data not reported

Summary statistics for each study

Table S 6. Summary statistics of studies: Task performance

	Baseline								Up to 4 weeks post-intervention								5-24 weeks post-intervention								More than 24 weeks post-intervention							
	MBP		Passive control		Placebo control		Active control		MBP		Passive control		Placebo control		Active control		MBP		Passive control		Placebo control		Active control		MBP		Passive control		Placebo control		Active control	
Study	n	M (SD)	n	M (SD)	n	M (SD)	n	M (SD)	n	M (SD)	n	M (SD)	n	M (SD)	n	M (SD)	n	M (SD)	n	M (SD)	n	M (SD)	n	M (SD)	n	M (SD)	n	M (SD)	n	M (SD)	n	M (SD)
Allexandre 2016	23	2.58 (0.47)	26	2.62 (0.64)		-		-	20	2.68 (0.44)	21	2.63 (0.49)		-		-	21	2.38 (0.63)	25	2.52 (0.65)		-		-		-		-		-		-
Asthana 2021		66.3 (10.2)		67.1 (10.4)		-		-		76.2 (12.4)		67.9 (11.3)		-		-		-		-		-		-		-		-		-		-
Augustus 2024		19.75 (4.2)		19.66 (4.04)		-		-		21.21 (3.66)		20.26 (2.76)		-		-		18.96 (4.28)		19.46 (3.22)		-		-		-		-		-		-
Baumgartner 2021		-		-		-		-		-		-		-		-		-		-		-		-		-		-		-		-
Braun 2020b		35.85 (33.26)		39.37 (29.7)		-		-		19.94 (28.48)		31.64 (33.21)		-		-		-		-		-		-		-		-		-		-
Calcagni 2021		5.06 (0.54)		5.12 (0.49)		-		-		5.21 (0.51)		4.89 (0.45)		-		-		-		-		-		-		-		-		-		-
Christodoulou 2024		22.4 (5.22)		22.97 (4.66)		-		22.64 (4.4)		25.12 (3.84)		24.04 (4.7)		-		25 (4.63)		26.46 (3.35)		24.18 (5.34)		-		25.37 (5.11)		-		-		-		-
Daigle 2018		-		-		-		-		-		-		-		-		-		-		-		-		-		-		-		-
Flook 2013		5.19 (0.58)		5.35 (0.77)		-		-		5.5 (0.45)		5.27 (1.11)		-		-		-		-		-		-		-		-		-		-
Galante 2018		-		-		-		-		-		-		-		-		-		-		-		-		-		-		-		-
Garrote-Caparrós 2022		42.82 (15.66)		-		38.61 (12.82)		-		34.72 (12.11)		-		39.55 (14.88)		-		36.14 (9.92)		-		39.56 (13.8)		-		-		-		-		-
Glass 2019		-		-		-		-		-		-		-		-		-		-		-		-		-		-		-		-
Grupe 2021		-		-		-		-		-		-		-		-		-		-		-		-		-		-		-		-
Hillhouse 2023		-		-		-		-		-		-		-		-		-		-		-		-		-		-		-		-
Huberty 2022	546	30.3 (27.93)	404	33.08 (28.44)		-		-	174	22.81 (24.08)	136	31.53 (28.94)		-		-		-		-		-		-		-		-		-		-
Janssen 2022	37	2.15 (0.71)	20	2.38 (0.61)		-		-		2.21 (0.68)		2.37 (0.81)		-		-		2.27 (0.7)		2.38 (0.66)		-		-		2.27 (0.73)		2.44 (0.74)		-		-

Study	Baseline MBP n	Baseline MBP M (SD)	Baseline Passive control n	Baseline Passive control M (SD)	Baseline Placebo control n	Baseline Placebo control M (SD)	Baseline Active control n	Baseline Active control M (SD)
Jennings 2017		4.86 (0.9)		4.97 (0.8)		-		-
Küchler 2023		63.22 (21.41)		63.09 (22.98)		-		-
Lebares 2019		103.83 (34.57)		-		-		129 (33.72)
Liu 2022		3.15 (0.84)		3.3 (0.84)		-		-
Nassif 2023 study 1		-		-		-		-
Nassif 2023 study 2		-		-		-		-
Nübold 2021	54	3.91 (0.75)	57	4.19 (0.72)		-	48	3.98 (0.75)
Pang 2019		5.84 (0.73)	19	5.91 (0.71)		-		-
Repo 2022		7.76 (1.05)		7.72 (1.4)		-		7.75 (1.48)
Roeser 2022		6.07 (0.68)		6.14 (0.75)		-		-
Sampl 2017		-		-		-		-
Schroeder 2018	16	9.12 (1.36)	17	9.01 (1.29)		-		-
Steinberg 2017		2.07 (2.84)		2.75 (7.4)		-		-
Takhdat 2024		-		-		-		-
Vainre 2024	66	77.4 (11.85)		-	53	77.32 (13.84)		-
Valley 2017		-		-		-		-
Verweij 2018	80	2.4 (0.6)	68	2.3 (0.5)		-		-
van Berkel 2014, van Dongen 2016		16.6 (2.3)		-		-		16.3 (2.6)

Study	Up to 4 weeks post-intervention MBP n	MBP M (SD)	Passive control n	Passive control M (SD)	Placebo control n	Placebo control M (SD)	Active control n	Active control M (SD)
Jennings 2017		-		-		-		-
Küchler 2023		69.7 (23.31)		66.14 (22.61)		-		-
Lebares 2019		96.92 (20.46)		-		-		122.44 (30.93)
Liu 2022		3.74 (0.85)		3.2 (0.8)		-		-
Nassif 2023 study 1		-		-		-		-
Nassif 2023 study 2		-		-		-		-
Nübold 2021	10	3.8 (0.61)	14	4.19 (0.55)		-	12	3.75 (1.15)
Pang 2019		5.9 (0.76)	16	5.91 (0.54)		-		-
Repo 2022		8 (1.16)		7.31 (1.41)		-		7.38 (1.86)
Roeser 2022		6.13 (0.67)		6.15 (0.61)		-		-
Sampl 2017	39	1.78 (0.53)	41	2.2 (0.77)		-		-
Schroeder 2018		-		-		-		-
Steinberg 2017		1.29 (1.89)		3.79 (10.52)		-		-
Takhdat 2024	12	33 (12.11)	8	25 (15.57)		-		-
Vainre 2024	36	82.43 (11.6)		-	32	80.18 (13.56)		-
Valley 2017		-		-		-		-
Verweij 2018		-		-		-		-
van Berkel 2014, van Dongen 2016		-		-		-		-

Study	5-24 weeks post-intervention MBP n	MBP M (SD)	Passive control n	Passive control M (SD)	Placebo control n	Placebo control M (SD)	Active control n	Active control M (SD)
Jennings 2017		5.13 (0.86)		5.01 (0.88)		-		-
Küchler 2023		70.07 (24.55)		68.94 (23.25)		-		-
Lebares 2019		-		-		-		-
Liu 2022		-		-		-		-
Nassif 2023 study 1		-		-		-		-
Nassif 2023 study 2		-		-		-		-
Nübold 2021		-		-		-		-
Pang 2019		6.04 (0.64)	16	6.15 (0.44)		-		-
Repo 2022		7.56 (1.81)		7.58 (1.64)		-		8.2 (1.26)
Roeser 2022		6.23 (0.58)		5.91 (0.84)		-		-
Sampl 2017		-		-		-		-
Schroeder 2018	13	9.19 (1.44)	13	9.04 (1.23)		-		-
Steinberg 2017		-		-		-		-
Takhdat 2024		-		-		-		-
Vainre 2024	33	82.49 (13.25)		-	29	78.26 (18.21)		-
Valley 2017		-		-		-		-
Verweij 2018	71	2.3 (0.6)	67	2.3 (0.6)		-		-
van Berkel 2014, van Dongen 2016		-		-		-		-

Study	More than 24 weeks post-intervention MBP n	MBP M (SD)	Passive control n	Passive control M (SD)	Placebo control n	Placebo control M (SD)	Active control n	Active control M (SD)
Jennings 2017		-		-		-		-
Küchler 2023		-		-		-		-
Lebares 2019		102.42 (16.64)		-		-		127.11 (41.11)
Liu 2022		-		-		-		-
Nassif 2023 study 1		-		-		-		-
Nassif 2023 study 2		-		-		-		-
Nübold 2021		-		-		-		-
Pang 2019		-		-		-		-
Repo 2022		-		-		-		-
Roeser 2022		-		-		-		-
Sampl 2017		-		-		-		-
Schroeder 2018		-		-		-		-
Steinberg 2017		-		-		-		-
Takhdat 2024	6	34 (16.44)	8	28 (30.28)		-		-
Vainre 2024		-		-		-		-
Valley 2017		-		-		-		-
Verweij 2018		-		-		-		-
van Berkel 2014, van Dongen 2016		-		-		-		-

Table S 7. Summary statistics of studies: Contextual performance

	Baseline								Up to 4 weeks post-intervention								5-24 weeks post-intervention								More than 24 weeks post-intervention							
	MBP		Passive control		Placebo control		Active control		MBP		Passive control		Placebo control		Active control		MBP		Passive control		Placebo control		Active control		MBP		Passive control		Placebo control		Active control	
**Study**	n	M (SD)	n	M (SD)	n	M (SD)	n	M (SD)	n	M (SD)	n	M (SD)	n	M (SD)	n	M (SD)	n	M (SD)	n	M (SD)	n	M (SD)	n	M (SD)	n	M (SD)	n	M (SD)	n	M (SD)	n	M (SD)
Aikens 2014		4.69 (1.01)		4.79 (1.13)		-		-		5.2 (0.89)		4.85 (1.03)		-		-		5.3 (0.78)		-		-		-		-		-		-		-
Baumgartner 2021		-		-		-		-		-		-		-		-		0.85 (0.35)		0.71 (0.46)		-		0.95 (0.21)		-		-		-		-
Bellosta-Batalla 2021		50 (12.55)		49.73 (14.07)		-		-		64.84 (17.05)		54.54 (14.33)		-		-		64.68 (15.14)		55.38 (13.25)		-		-		-		-		-		-
Benn 2012	19	3.84 (0.55)	16	3.8 (0.52)		-		-	19	4.06 (0.55)	9	3.64 (0.43)		-		-	14	4.28 (0.56)	9	3.33 (0.9)		-		-		-		-		-		-
Braun 2020a	76	2.82 (0.77)	88	2.81 (0.79)		-		-		-		-		-		-	75	3.23 (0.7)	85	2.82 (0.87)		-		-	69	3.19 (0.73)	75	2.86 (0.79)		-		-
Brown 2016	23	41.52 (10.29)		-	15	41.07 (9.79)		-	19	40.53 (10.81)		-	15	42.53 (11.03)		-	19	40.42 (10.32)		-	15	41.36 (10.92)		-		-		-		-		-
Calcagni 2021		4.18 (0.74)		4.2 (0.76)		-		-		4.69 (0.84)		4.17 (0.81)		-		-		-		-		-		-		-		-		-		-
Desai 2024	21	4.22 (1.96)		-		-	11	4.28 (1.12)	21	4.47 (2.02)		-		-	11	4.34 (1.46)		-		-		-		-		-		-		-		-
Erden 2023		48.42 (5.39)		48.85 (5.63)		-		-		59.52 (6.52)		47.66 (4.49)		-		-		-		-		-		-		-		-		-		-
Flook 2013		3.49 (0.5)		3.98 (0.6)		-		-		3.69 (0.54)		3.84 (1)		-		-		-		-		-		-		-		-		-		-
Garrote-Caparrós 2022		25.18 (3.24)		-		25.16 (2.94)		-		26.46 (2.08)		-		24.45 (3.37)		-		24.69 (3.59)		-		24.86 (3.95)		-		-		-		-		-
Hwang 2019		6.84 (1.15)		7.22 (1.06)		-		-		7.03 (1.07)		7.35 (1.26)		-		-		6.77 (1.13)		7.23 (1.28)		-		-		-		-		-		-
Jennings 2013		6.69 (1.09)		6.92 (1.12)		-		-		7.13 (1.05)		6.7 (1.04)		-		-		-		-		-		-		-		-		-		-
Jennings 2017		2.75 (0.67)		2.77 (0.71)		-		-		-		-		-		-		2.49 (0.65)		2.51 (0.65)		-		-		-		-		-		-
Karing 2021		26.57 (4.24)		27.92 (4.19)		-		-		29.87 (3.32)		29.18 (2.76)		-		-		29.66 (3.46)		28.61 (2.8)		-		-		-		-		-		-
Klatt 2015		-		-		-		-		-		-		-		-		-		-		-		-		-		-		-		-
Klatt 2017	27	4.55 (0.76)	30	4.59 (0.68)		-		-	26	4.71 (0.78)	30	4.57 (0.66)		-		-		-		-		-		-		-		-		-		-

	Baseline								Up to 4 weeks post-intervention								5-24 weeks post-intervention								More than 24 weeks post-intervention							
	MBP		Passive control		Placebo control		Active control		MBP		Passive control		Placebo control		Active control		MBP		Passive control		Placebo control		Active control		MBP		Passive control		Placebo control		Active control	
Study	n	M (SD)	n	M (SD)	n	M (SD)	n	M (SD)	n	M (SD)	n	M (SD)	n	M (SD)	n	M (SD)	n	M (SD)	n	M (SD)	n	M (SD)	n	M (SD)	n	M (SD)	n	M (SD)	n	M (SD)	n	M (SD)
Kuyken 2022		7.35 (1.06)		-		-		7.32 (1.05)	258	7.45 (0.92)		-		-	215	7.32 (1.12)	197	7.45 (1.03)		-		-	174	7.28 (1.17)	204	7.53 (0.94)		-		-	162	7.38 (1.03)
Küchler 2023		26.03 (4.53)		25.74 (4.43)		-		-		28.99 (4.69)		25.79 (4.82)		-		-		29.08 (4.99)		26.21 (5.15)		-		-		-		-		-		-
Lensen 2024		5.21 (0.59)		5.26 (0.6)		-		-		5.75 (0.59)		5.32 (0.6)		-		-		5.97 (0.59)		5.49 (0.6)		-		-		-		-		-		-
Modrego-Alarcón 2021		58.13 (15.1)		-		-	92	58.71 (14.07)		62.1 (15.97)		-		-	61	59.85 (14.3)		65.47 (16.23)		-		-	51	60.67 (13.73)		-		-		-		-
Phang 2015		30.49 (4.27)		29.03 (4.28)		-		-		32.15 (3.77)		28.36 (4.47)		-		-		31.81 (4.3)		28.97 (4.38)		-		-		-		-		-		-
Pipe 2009		0 (0.34)		-		0 (0.38)		-		0.19 (0.34)		-		0.19 (0.38)		-		-		-		-		-		-		-		-		-
Rich 2021	62	69.66 (11.73)	63	71.71 (10.89)		-		-		70.16 (11.53)		71.37 (12.14)		-		-		-		-		-		-		-		-		-		-
Sampl 2017		4.21 (0.16)		4.36 (0.87)		-		-		4.66 (0.16)		4.16 (1.09)		-		-		-		-		-		-		-		-		-		-
Steinberg 2017		-		-		-		-		-		-		-		-		-		-		-		-		-		-		-		-
Valley 2017		-		-		-		-		-		-		-		-		-		-		-		-		-		-		-		-
de Carvalho 2021		6.66 (0.87)		6.63 (0.76)		-		-		6.8 (0.83)		5.6 (1.27)		-		-		-		-		-		-		-		-		-		-
de Jong 2013		90.67 (13.07)		90.37 (13.33)		-		-		96.67 (9.08)		89.14 (17.01)		-		-		-		-		-		-		-		-		-		-
van Berkel 2014, van Dongen 2016	129	4.1 (0.8)		-		-	128	4 (0.9)		-		-		-		-	115	4 (0.9)		-		-	108	4 (0.9)	120	3.9 (0.9)		-		-	112	4 (0.9)

Table S 8. Summary statistics of studies included: Adaptive performance

	Baseline								Up to 4 weeks post-intervention								5-24 weeks post-intervention								More than 24 weeks post-intervention							
	MBP		Passive control		Placebo control		Active control		MBP		Passive control		Placebo control		Active control		MBP		Passive control		Placebo control		Active control		MBP		Passive control		Placebo control		Active control	
Study	n	M (SD)	n	M (SD)	n	M (SD)	n	M (SD)	n	M (SD)	n	M (SD)	n	M (SD)	n	M (SD)	n	M (SD)	n	M (SD)	n	M (SD)	n	M (SD)	n	M (SD)	n	M (SD)	n	M (SD)	n	M (SD)
Aikens 2014		68.5 (12.09)		71.02 (14.29)		-		-		76.11 (12.14)		67.71 (14.7)		-		-		76.71 (11.39)		-		-		-		-		-		-		-
AlQarni 2023		26.53 (7.63)		-		24.94 (8.62)		-		26.36 (8.33)		-		27.21 (9.28)		-		-		-		-		-		-		-		-		-
Asuero 2014	43	119.7 (12.8)	25	120.8 (10.1)		-		-	43	123 (9.2)	25	119 (10.7)		-		-		-		-		-		-		-		-		-		-
Barczak-Scarboro 2021		-		-		-		-		-		-		-		-		-		-		-		-		-		-		-		-
Bonde 2022	95	4.3 (0.9)	92	4.3 (0.8)		-		-		-		-		-		-	73	4.4 (0.9)	71	4.3 (0.9)		-		-		-		-		-		-
Can Gür 2020		95.11 (13.94)		94.09 (15.54)		-		-		101.14 (15.19)		96.25 (16.38)		-		-		-		-		-		-		-		-		-		-
Chan 2021		18.5 (3.27)		16.22 (5.21)		-		-		-		-		-		-		-		-		-		-		-		-		-		-
Choi 2024		-		-		-		-		-		-		-		-		-		-		-		-		-		-		-		-
Christopher 2018	31	81.48 (12.36)	30	76.1 (9.34)		-		-	24	83.66 (10.73)	26	77.07 (9.5)		-		-	24	83.2 (11.38)	25	77.48 (10.19)		-		-		-		-		-		-
Christopher 2024		-		-		-		-		-		-		-		-		-		-		-		-		-		-		-		-
Dvořáková 2017		3.8 (0.39)		3.78 (0.44)		-		-		3.8 (0.46)		3.76 (0.47)		-		-		-		-		-		-		-		-		-		-
Erogul 2014		78.1 (9.1)		76.3 (11)		-		-		80.5 (10)		77.1 (14.1)		-		-		82.4 (9.8)		77.3 (12.5)		-		-		-		-		-		-
Fazia 2023	174	66.56 (13.57)	188	65.66 (13.01)		-		-	174	69.01 (13.34)	188	64.32 (13.39)		-		-	43	71.44 (14.17)	44	61.66 (13.34)		-		-		-		-		-		-
Flook 2013		4.92 (0.57)		5.38 (0.49)		-		-		5.25 (0.76)		5.05 (0.7)		-		-		-		-		-		-		-		-		-		-
Fraiman 2022		18 (2)		-		17.9 (2.2)		-		-		-		-		-		-		-		-		-		-		-		-		-
Garrote-Caparrós 2022		82.86 (11.05)		-		83.81 (8.56)		-		84.04 (7.12)		-		80.19 (11.26)		-		81.62 (8.08)		-		79.65 (13.22)		-		-		-		-		-

	Baseline								Up to 4 weeks post-intervention								5-24 weeks post-intervention								More than 24 weeks post-intervention							
	MBP		Passive control		Placebo control		Active control		MBP		Passive control		Placebo control		Active control		MBP		Passive control		Placebo control		Active control		MBP		Passive control		Placebo control		Active control	
Study	n	M (SD)	n	M (SD)	n	M (SD)	n	M (SD)	n	M (SD)	n	M (SD)	n	M (SD)	n	M (SD)	n	M (SD)	n	M (SD)	n	M (SD)	n	M (SD)	n	M (SD)	n	M (SD)	n	M (SD)	n	M (SD)
Godara 2024		3.55 (0.76)		3.34 (0.84)		3.34 (0.79)		-		3.75 (0.73)		3.39 (0.88)		3.47 (0.81)		-		-		-		-		-		-		-		-		-
Grupe 2021		-		-		-		-		-		-		-		-		-		-		-		-		-		-		-		-
Gómez-Odriozola 2019		-		-		-		-		-		-		-		-		-		-		-		-		-		-		-		-
Hillhouse 2023		-		-		-		-		-		-		-		-		-		-		-		-		-		-		-		-
Huberty 2022	583	3.31 (0.8)	443	3.29 (0.88)		-		-	199	3.51 (0.78)	155	3.43 (0.99)		-		-		-		-		-		-		-		-		-		-
Jennings 2017		4.92 (0.8)		5 (0.7)		-		-		-		-		-		-		4.92 (0.76)		4.81 (0.74)		-		-		-		-		-		-
Jia-Yuan 2022		85.56 (3.19)		84.64 (4.05)		-		-		91 (3.12)		84.97 (3.92)		-		-		93.78 (2.81)		85.08 (3.34)		-		-		-		-		-		-
Juul 2021	34	3.59 (1.07)	33	3.72 (0.84)		-		-		-		-		-		-	28	3.74 (1.01)	28	0.72 (0.79)		-		-		-		-		-		-
Klatt 2015		-		-		-		-		-		-		-		-		-		-		-		-		-		-		-		-
Kor 2019	18	18.72 (5.21)		-	18	17.78 (4.11)		-	18	18.94 (5.21)		-	18	19.06 (3.95)		-	18	19.66 (5.07)		-	18	17.69 (3.47)		-		-		-		-		-
Kor 2020	56	14.59 (4.32)		-	57	14.53 (4.46)		-	51	13.33 (5.45)		-	53	14.04 (5.46)		-	50	15.65 (2.81)		-	51	14.09 (4.99)		-		-		-		-		-
Lebares 2019		31.33 (5.11)		-		-		31.44 (3.4)		32.58 (5.44)		-		-		30.89 (2.89)		-		-		-		-		32.75 (5.59)		-		-		31.33 (3.12)
Lin 2019	44	54.43 (11.46)	46	55.17 (11.85)		-		-	44	57.98 (11.58)	46	55.11 (12.8)		-		-	44	59.7 (11.87)	46	53.85 (16.21)		-		-		-		-		-		-
Liu 2023		7.03 (14.33)		6.19 (10.52)		-		-		11.89 (12.89)		7.55 (14.03)		-		-		-		-		-		-		-		-		-		-
Nadler 2020	37	3.29 (0.77)	65	3.63 (0.71)		-		-		3.78 (0.69)	65	3.41 (0.75)		-		-		-		-		-		-		-		-		-		-
Nielsen 2021	45	3.28 (0.77)	48	3.43 (0.72)		-		-	26	3.61 (0.82)	40	3.56 (0.85)		-		-	21	3.77 (0.68)	21	3.67 (0.66)		-		-		-		-		-		-
Orosa-Duarte 2021		122.93 (16.62)		123.57 (12.05)		-		-		122.83 (17.02)		122.43 (12.91)		-		-		-		-		-		-		-		-		-		-
Perez-Blasco 2016	22	2.51 (0.5)	23	2.87 (0.51)		-		-	20	3.36 (0.51)	23	2.86 (0.5)		-		-		-		-		-		-		-		-		-

	Baseline								Up to 4 weeks post-intervention								5-24 weeks post-intervention								More than 24 weeks post-intervention							
	MBP		Passive control		Placebo control		Active control		MBP		Passive control		Placebo control		Active control		MBP		Passive control		Placebo control		Active control		MBP		Passive control		Placebo control		Active control	
Study	n	M (SD)	n	M (SD)	n	M (SD)	n	M (SD)	n	M (SD)	n	M (SD)	n	M (SD)	n	M (SD)	n	M (SD)	n	M (SD)	n	M (SD)	n	M (SD)	n	M (SD)	n	M (SD)	n	M (SD)	n	M (SD)
Pérula-de Torres 2021		121.55 (14.02)		124.14 (8.53)		-		-		124.56 (12.34)		124.35 (8.4)		-		-		124.79 (16.19)		123.96 (8.06)		-		-		-		-		-		-
Repo 2022		5.72 (0.71)		-		-		5.72 (0.7)		5.72 (0.77)		-		-		5.56 (0.74)		5.65 (0.79)		-		-		5.27 (1.14)		-		-		-		-
Rodrigues de Oliveira 2021		54.24 (14.53)		-		-		59.05 (9.69)		71.71 (13.22)		-		-		59.1 (10.88)		-		-		-		-		-		-		-		-
Roeser 2022		4.38 (0.69)		4.32 (0.65)		-		-		4.34 (0.67)		4.3 (0.71)		-		-		4.27 (0.64)		4.24 (0.7)		-		-		-		-		-		-
Schroeder 2018	16	26.31 (4.51)	17	27 (4.97)		-		-	15	27.66 (3.22)	14	26.07 (4.73)		-		-	13	27.84 (4.09)	13	25.07 (5.85)		-		-		-		-		-		-
Shapiro 1998		-		-		-		-		-		-		-		-		-		-		-		-		-		-		-		-
Shapiro 2011		2.78 (0.49)		2.75 (0.6)		-		-		2.82 (0.6)		2.8 (0.54)		-		-		2.82 (0.62)		2.79 (0.6)		-		-		2.85 (0.6)		2.78 (0.58)		-		-
Shapiro 2019		-		-		-		-		-		-		-		-		-		-		-		-		-		-		-		-
Strauss 2021		83.78 (7.47)		84.22 (7.85)		-		-		83.97 (7.42)		83.24 (7.62)		-		-		-		-		-		-		-		-		-		-
Taylor 2016		3.77 (0.71)		3.65 (0.68)		-		-		3.89 (0.72)		3.73 (0.77)		-		-		-		-		-		-		-		-		-		-
Taylor 2022	1,094	4.78 (1.09)		-		-	1,085	4.77 (1.1)	504	4.69 (1.17)		-		-	518	4.5 (1.24)		-		-		-		-		-		-		-		-
Verweij 2018		5.85 (0.77)		5.78 (0.81)		-		-		-		-		-		-		5.9 (0.85)		5.85 (0.81)		-		-		-		-		-		-
Wang 2023		60.23 (8.6)		-		59.53 (7.39)		-		68.06 (8.55)		-		61.79 (8.38)		-		-		-		-		-		-		-		-		-
Watson-Singleton 2024		2.74 (0.85)		2.58 (0.89)		-		-		3.45 (0.97)		3.54 (0.92)		-		-		-		-		-		-		-		-		-		-
Wilson 2022		53 (12.77)		-		-		56 (9.54)		72 (14.99)		-		-		52 (10.63)		-		-		-		-		-		-		-		-
dos Santos 2024	18	87.22 (17.78)		99.64 (12.06)		-		-		-		-		-		-		-		-		-		-		-		-		-		-
van Dijk 2017		110.3 (10.3)		110.3 (9.3)		-		-		111.9 (9.7)		108.4 (10)		-		-		110.9 (11.5)		109.8 (8.6)		-		-		112 (11.6)		108.9 (11.2)		-		-

Table S 9. Summary statistics of studies included: Counterproductive work behaviour

	Baseline								Up to 4 weeks post-intervention								5-24 weeks post-intervention								More than 24 weeks post-intervention							
	MBP		Passive control		Placebo control		Active control		MBP		Passive control		Placebo control		Active control		MBP		Passive control		Placebo control		Active control		MBP		Passive control		Placebo control		Active control	
**Study**	n	M (SD)	n	M (SD)	n	M (SD)	n	M (SD)	n	M (SD)	n	M (SD)	n	M (SD)	n	M (SD)	n	M (SD)	n	M (SD)	n	M (SD)	n	M (SD)	n	M (SD)	n	M (SD)	n	M (SD)	n	M (SD)
Balci 2023		27.8 (2.4)		27.9 (2.05)		-		-		-		-		-		-		-		-		-		-		-		-		-		-
Bartlett 2017	20	2.94 (5.18)		-		-	66	1.62 (3.52)	20	1.88 (2.8)		-		-	66	2.59 (3.69)		-		-		-		-		-		-		-		-
Can Gür 2020		69.09 (7.82)		71.24 (7.09)		-		-		69.26 (7.22)		69.2 (7.8)		-		-		-		-		-		-		-		-		-		-
Choi 2022		3.69 (0.16)		3.68 (0.12)		3.75 (0.13)		-		3.79 (0.12)		3.58 (0.13)		3.55 (0.13)		-		-		-		-		-		-		-		-		-
Hunsinger 2019	31	0.84 (0.09)	30	0.82 (0.05)		-		-	24	0.83 (0.07)	26	0.82 (0.11)		-		-	24	0.86 (0.07)	25	0.83 (0.07)		-		-		-		-		-		-
Küchler 2023		5.7 (11.77)		6.31 (11.99)		-		-		6.1 (14.91)		6.02 (17.56)		-		-		4.86 (12.75)		4.52 (10.79)		-		-		-		-		-		-
Liu 2022		-		-		-		-		5.07 (0.44)		4.8 (0.39)		-		-		-		-		-		-		-		-		-		-
Ogino 2024		75.5 (22.81)		74.4 (21.56)		-		-		79.3 (22.5)		74.1 (21.25)		-		-		-		-		-		-		-		-		-		-
Rad 2023		40.93 (5.15)		40.4 (4.86)		-		-		29.46 (5.36)		40.66 (5.55)		-		-		-		-		-		-		-		-		-		-
Roeser 2013		3.03 (3.67)		3.85 (4.67)		-		-		-		-		-		-		-		-		-		-		-		-		-		-
Steinberg 2017		1.06 (1.88)		2.56 (7.39)		-		-		0.78 (1.35)		0.69 (1.08)		-		-		-		-		-		-		-		-		-		-
Strauss 2021	77	5.31 (6.04)	91	4.53 (6.21)		-		-	77	4.36 (5.87)	91	4.46 (5.48)		-		-		-		-		-		-		-		-		-		-
Taylor 2022	1,095	2.35 (7.08)		-		-	1,086	2.44 (7.45)	593	2.23 (7.99)		-		-	573	2.04 (6.86)		-		-		-		-		-		-		-		-
van Berkel 2014, van Dongen 2016		7.1 (25.7)		-		-		3.8 (9.5)		-		-		-		-		-		-		-		-		-		-		-		-

Main outcome

Passive control groups

```
## Review:     Mindfulness interventions for task performance
##
##                           SMD             95%-CI %W(random)
## Allexandre 2016    -0.0658 [-0.2146;  0.0830]        6.7
## Asthana 2021        0.6762 [-0.0147;  1.3671]        3.5
## Augustus 2024       0.2762 [-0.1261;  0.6785]        5.2
## Braun 2020b         0.3917 [-2.2417;  3.0250]        0.4
## Calcagni 2021       0.6012 [ 0.2968;  0.9057]        5.9
## Christodoulou 2024 -0.2749 [-0.5059; -0.0439]        6.3
## Flook 2013          0.2376 [-0.1100;  0.5851]        5.6
## Glass 2019          0.0000                           0.0
## Grupe 2021          0.2116 [-0.3091;  0.7323]        4.5
## Hillhouse 2023      0.5749 [ 0.0831;  1.0666]        4.7
## Huberty 2022        0.3466 [-1.9531;  2.6463]        0.6
## Janssen 2022        0.2768 [ 0.0750;  0.4785]        6.5
## Küchler 2023        0.1525 [-0.1251;  0.4302]        6.1
## Liu 2022            0.5853 [ 0.2951;  0.8755]        6.0
## Nübold 2021        -0.7280 [-0.9911; -0.4648]        6.1
## Pang 2019           0.0130 [-0.3126;  0.3385]        5.7
## Repo 2022           0.5194 [ 0.2302;  0.8085]        6.0
## Roeser 2022         0.0091 [-0.3667;  0.3848]        5.4
## Sampl 2017          0.7384 [ 0.2060;  1.2708]        4.4
## Steinberg 2017      0.3033 [-0.3215;  0.9280]        3.8
## Takhdat 2024        0.5750 [ 0.1578;  0.9922]        5.1
## Valley 2017         1.5038 [ 0.1366;  2.8711]        1.4
##
## Number of studies: k = 21
##
##                                   SMD            95%-CI    t p-value
## Random effects model (HK-SE) 0.2496 [ 0.0559; 0.4433] 2.69  0.0141
## Prediction interval                 [-0.5081; 1.0073]
##
## Quantifying heterogeneity:
##  tau^2 = 0.1224 [0.0490; 0.2845]; tau = 0.3499 [0.2214; 0.5334]
##  I^2 = 81.5% [72.6%; 87.5%]; H = 2.32 [1.91; 2.83]
##
## Test of heterogeneity:
##       Q d.f.  p-value
##  108.01   20 < 0.0001
##
## Details on meta-analytical method:
## - Inverse variance method
```

```
## - Restricted maximum-likelihood estimator for tau^2
## - Q-Profile method for confidence interval of tau^2 and tau
## - Hartung-Knapp adjustment for random effects model (df = 20)
## - Prediction interval based on t-distribution (df = 19)
```

Active control groups

```
summary(model_TPspec_upto4w)

## Review:     Mindfulness interventions for task performance
##
##                        SMD              95%-CI %W(random)
## Christodoulou 2024 -0.0394 [ -0.2322;  0.1535]       34.4
## Lebares 2019        1.1801 [-10.5666; 12.9269]        0.0
## Nübold 2021         0.0299 [ -0.1356;  0.1954]       36.2
## Repo 2022           0.4198 [  0.1529;  0.6866]       29.4
##
## Number of studies: k = 4
##
##                                 SMD            95%-CI    t p-value
## Random effects model (HK-SE) 0.1212 [-0.3035; 0.5458] 0.91  0.4308
## Prediction interval                 [-0.9317; 1.1741]
##
## Quantifying heterogeneity:
##  tau^2 = 0.0421 [0.0000; 0.6431]; tau = 0.2051 [0.0000; 0.8019]
##  I^2 = 62.9% [0.0%; 87.5%]; H = 1.64 [1.00; 2.83]
##
## Test of heterogeneity:
##     Q d.f. p-value
##  8.08    3  0.0444
##
## Details on meta-analytical method:
## - Inverse variance method
## - Restricted maximum-likelihood estimator for tau^2
## - Q-Profile method for confidence interval of tau^2 and tau
## - Hartung-Knapp adjustment for random effects model (df = 3)
## - Prediction interval based on t-distribution (df = 2)
```

Subgroup analyses

Length of intervention

To investigate the effect of MBP duration on the outcome, we converted the duration into hours of guided content. The duration of self-help MBPs was calculated by multiplying the duration of guided meditation multiplied by that number of days a week the participants were asked to practice meditation and the number of weeks the intervention was intended to last. We excluded the duration of unguided mediation, as studies rarely quantified its duration. For face-to-face or other human-

taught synchronously delivered programmes, we only included synchronously delivered sessions to estimate duration, that is, leaving out independent home practice.

```
Mixed-Effects Model (k = 21; tau^2 estimator: REML)
##
##   logLik  deviance       AIC       BIC      AICc
## -10.7302   21.4605   27.4605   30.2938   29.0605
##
## tau^2 (estimated amount of residual heterogeneity):     0.1085 (SE = 0.0501)
## tau (square root of estimated tau^2 value):             0.3295
## I^2 (residual heterogeneity / unaccounted variability): 79.06%
## H^2 (unaccounted variability / sampling variability):   4.78
## R^2 (amount of heterogeneity accounted for):            11.34%
##
## Test for Residual Heterogeneity:
## QE(df = 19) = 85.2786, p-val < .0001
##
## Test of Moderators (coefficient 2):
## F(df1 = 1, df2 = 19) = 3.3255, p-val = 0.0840
##
## Model Results:
##
##                                    estimate      se    tval  df    pval    ci.lb

## intrcpt                              0.0018  0.1579  0.0115  19  0.9909  -0.3287

## duration_hrs_taught_intervention    0.0168  0.0092  1.8236  19  0.0840  -0.0025

##                                     ci.ub
## intrcpt                            0.3323
## duration_hrs_taught_intervention  0.0361  .
##
## ---
## Signif. codes:  0 '***' 0.001 '**' 0.01 '*' 0.05 '.' 0.1 ' ' 1
```

Setting

```
## Review:     Mindfulness interventions for task performance
##
##                         SMD             95%-CI %W(random) Sample.context
## Allexandre 2016    -0.0658 [-0.2146;  0.0830]        6.7   Occupational
## Asthana 2021        0.6762 [-0.0147;  1.3671]        3.5    Educational
## Augustus 2024       0.2762 [-0.1261;  0.6785]        5.2    Educational
## Braun 2020b         0.3917 [-2.2417;  3.0250]        0.4    Educational
## Calcagni 2021       0.6012 [ 0.2968;  0.9057]        5.9   Occupational
## Christodoulou 2024 -0.2749 [-0.5059; -0.0439]        6.3   Occupational
## Flook 2013          0.2376 [-0.1100;  0.5851]        5.6   Occupational
```

```
## Glass 2019         0.0000                              0.0    Educational
## Grupe 2021         0.2116 [-0.3091;  0.7323]        4.5   Occupational
## Hillhouse 2023     0.5749 [ 0.0831;  1.0666]        4.7   Occupational
## Huberty 2022       0.3466 [-1.9531;  2.6463]        0.6   Occupational
## Janssen 2022       0.2768 [ 0.0750;  0.4785]        6.5   Occupational
## Küchler 2023       0.1525 [-0.1251;  0.4302]        6.1    Educational
## Liu 2022           0.5853 [ 0.2951;  0.8755]        6.0   Occupational
## Nübold 2021       -0.7280 [-0.9911; -0.4648]        6.1   Occupational
## Pang 2019          0.0130 [-0.3126;  0.3385]        5.7   Occupational
## Repo 2022          0.5194 [ 0.2302;  0.8085]        6.0    Educational
## Roeser 2022        0.0091 [-0.3667;  0.3848]        5.4   Occupational
## Sampl 2017         0.7384 [ 0.2060;  1.2708]        4.4    Educational
## Steinberg 2017     0.3033 [-0.3215;  0.9280]        3.8   Occupational
## Takhdat 2024       0.5750 [ 0.1578;  0.9922]        5.1    Educational
## Valley 2017        1.5038 [ 0.1366;  2.8711]        1.4   Occupational
##
## Number of studies: k = 21
##
##                                   SMD            95%-CI    t p-value
## Random effects model (HK-SE) 0.2496 [ 0.0559; 0.4433] 2.69  0.0141
## Prediction interval                 [-0.5081; 1.0073]
##
## Quantifying heterogeneity:
##  tau^2 = 0.1224 [0.0490; 0.2845]; tau = 0.3499 [0.2214; 0.5334]
##  I^2 = 81.5% [72.6%; 87.5%]; H = 2.32 [1.91; 2.83]
##
## Test of heterogeneity:
##       Q d.f.  p-value
##  108.01   20 < 0.0001
##
## Results for subgroups (random effects model (HK-SE)):
##                                   k    SMD            95%-CI  tau^2    tau     Q
## Sample.context = Occupational  14 0.1603 [-0.0964; 0.4170] 0.1432 0.3784 85.01
## Sample.context = Educational    7 0.4282 [ 0.1848; 0.6716] 0.0186 0.1365  6.91
##                                   I^2
## Sample.context = Occupational 84.7%
## Sample.context = Educational  13.1%
##
## Test for subgroup differences (random effects model (HK-SE)):
##                    Q d.f. p-value
## Between groups 2.99    1  0.0838
##
## Details on meta-analytical method:
## - Inverse variance method
## - Restricted maximum-likelihood estimator for tau^2
## - Q-Profile method for confidence interval of tau^2 and tau
```

```
## - Hartung-Knapp adjustment for random effects model (df = 20)
## - Prediction interval based on t-distribution (df = 19)
```

Reporter type

One study (Allexandre et al., 2016) did not clearly report who provided the task performance ratings. This study was excluded from the analysis.

```
## Review:     Mindfulness interventions for task performance. Subgroup analysi ..
.
##
##                          SMD             95%-CI %W(random) report.type_recoded
## Asthana 2021          0.6762 [-0.0147;  1.3671]        3.8   Not self-reported
## Augustus 2024         0.2762 [-0.1261;  0.6785]        5.6       Self-reported
## Braun 2020b           0.3917 [-2.2417;  3.0250]        0.5       Self-reported
## Calcagni 2021         0.6012 [ 0.2968;  0.9057]        6.3       Self-reported
## Christodoulou 2024 -0.2749 [-0.5059; -0.0439]        6.8       Self-reported
## Flook 2013            0.2376 [-0.1100;  0.5851]        6.0   Not self-reported
## Glass 2019            0.0000                           0.0   Not self-reported
## Grupe 2021            0.2116 [-0.3091;  0.7323]        4.8       Self-reported
## Hillhouse 2023        0.5749 [ 0.0831;  1.0666]        5.0       Self-reported
## Huberty 2022          0.3466 [-1.9531;  2.6463]        0.6       Self-reported
## Janssen 2022          0.2768 [ 0.0750;  0.4785]        6.9       Self-reported
## Küchler 2023          0.1525 [-0.1251;  0.4302]        6.5       Self-reported
## Liu 2022              0.5853 [ 0.2951;  0.8755]        6.4       Self-reported
## Nübold 2021          -0.7280 [-0.9911; -0.4648]        6.6       Self-reported
## Pang 2019             0.0130 [-0.3126;  0.3385]        6.2   Not self-reported
## Repo 2022             0.5194 [ 0.2302;  0.8085]        6.4       Self-reported
## Roeser 2022           0.0091 [-0.3667;  0.3848]        5.8   Not self-reported
## Sampl 2017            0.7384 [ 0.2060;  1.2708]        4.7   Not self-reported
## Steinberg 2017        0.3033 [-0.3215;  0.9280]        4.2       Self-reported
## Takhdat 2024          0.5750 [ 0.1578;  0.9922]        5.5   Not self-reported
## Valley 2017           1.5038 [ 0.1366;  2.8711]        1.5       Self-reported
##
## Number of studies: k = 20
##
##                                   SMD            95%-CI    t p-value
## Random effects model (HK-SE) 0.2732 [ 0.0698; 0.4766] 2.81  0.0112
## Prediction interval                 [-0.4999; 1.0463]
##
## Quantifying heterogeneity:
##  tau^2 = 0.1260 [0.0483; 0.2950]; tau = 0.3549 [0.2197; 0.5431]
##  I^2 = 81.0% [71.5%; 87.3%]; H = 2.29 [1.87; 2.80]
##
## Test of heterogeneity:
##      Q d.f.  p-value
##  99.76   19 < 0.0001
```

```
##
## Results for subgroups (random effects model (HK-SE)):
##                                              k    SMD            95%-CI  tau^2
## report.type_recoded = Not self-reported   6 0.3142 [-0.0230; 0.6514] 0.0532
## report.type_recoded = Self-reported      14 0.2508 [-0.0301; 0.5318] 0.1613
##                                             tau     Q   I^2
## report.type_recoded = Not self-reported 0.2306 10.63 52.9%
## report.type_recoded = Self-reported     0.4016 87.96 85.2%
##
## Test for subgroup differences (random effects model (HK-SE)):
##                   Q d.f. p-value
## Between groups 0.12    1  0.7315
##
## Details on meta-analytical method:
## - Inverse variance method
## - Restricted maximum-likelihood estimator for tau^2
## - Q-Profile method for confidence interval of tau^2 and tau
## - Hartung-Knapp adjustment for random effects model (df = 19)
## - Prediction interval based on t-distribution (df = 18)
```

Secondary outcome analyses

Task performance

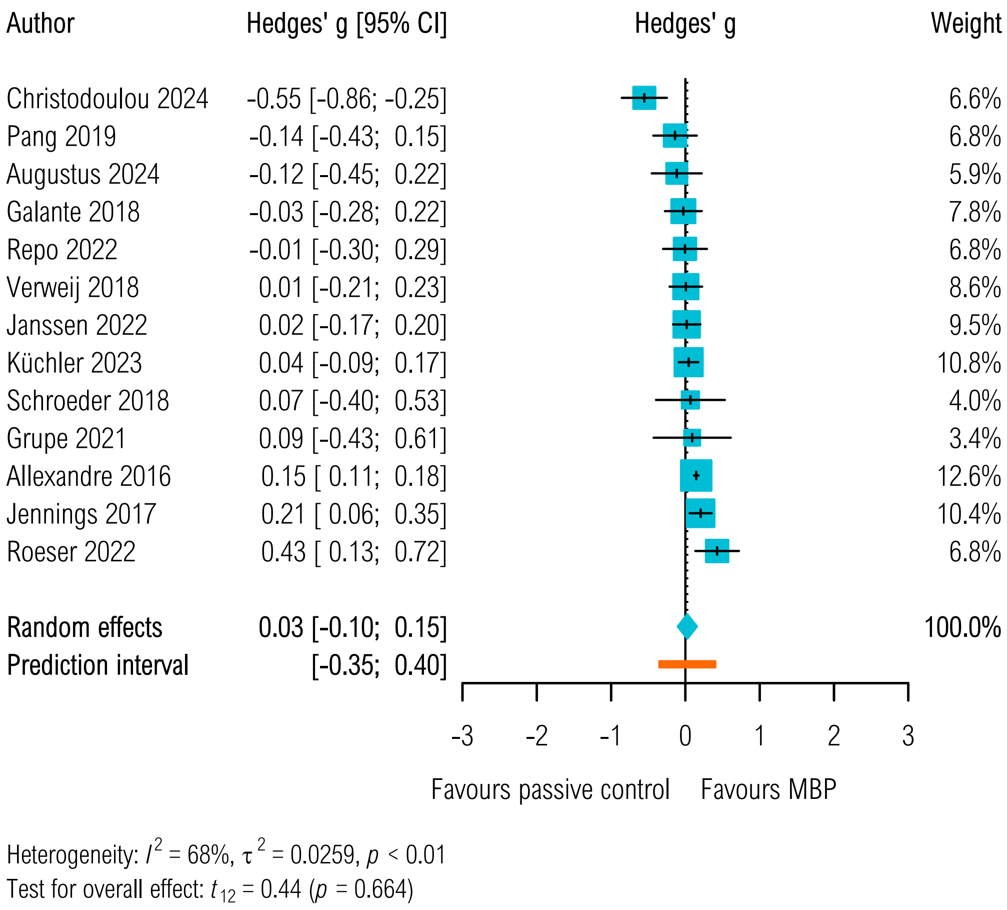

Figure S 3. Task performance 5-24 weeks post-intervention. Only passive control groups

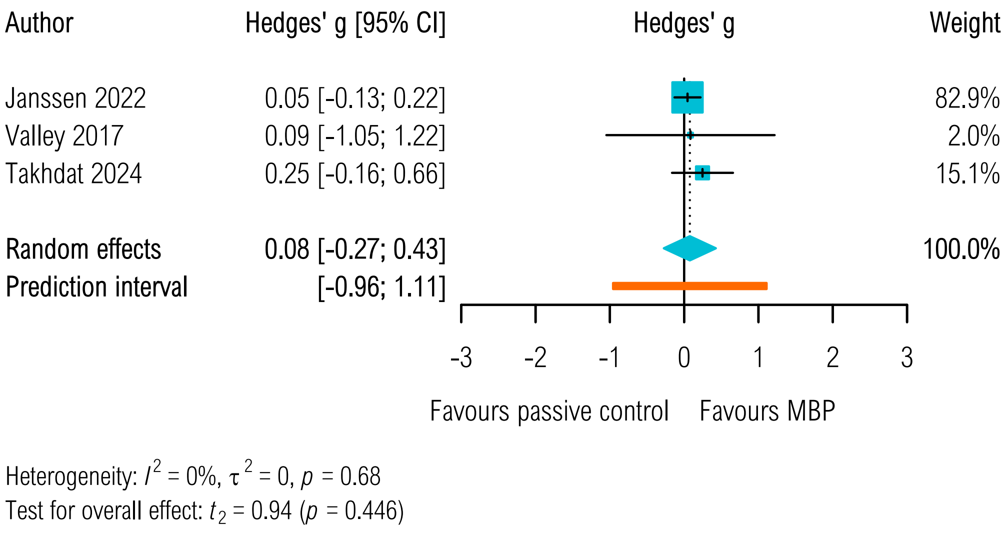

Figure S 4. Task performance more than 24 weeks post-intervention. Only passive control groups.

Contextual performance

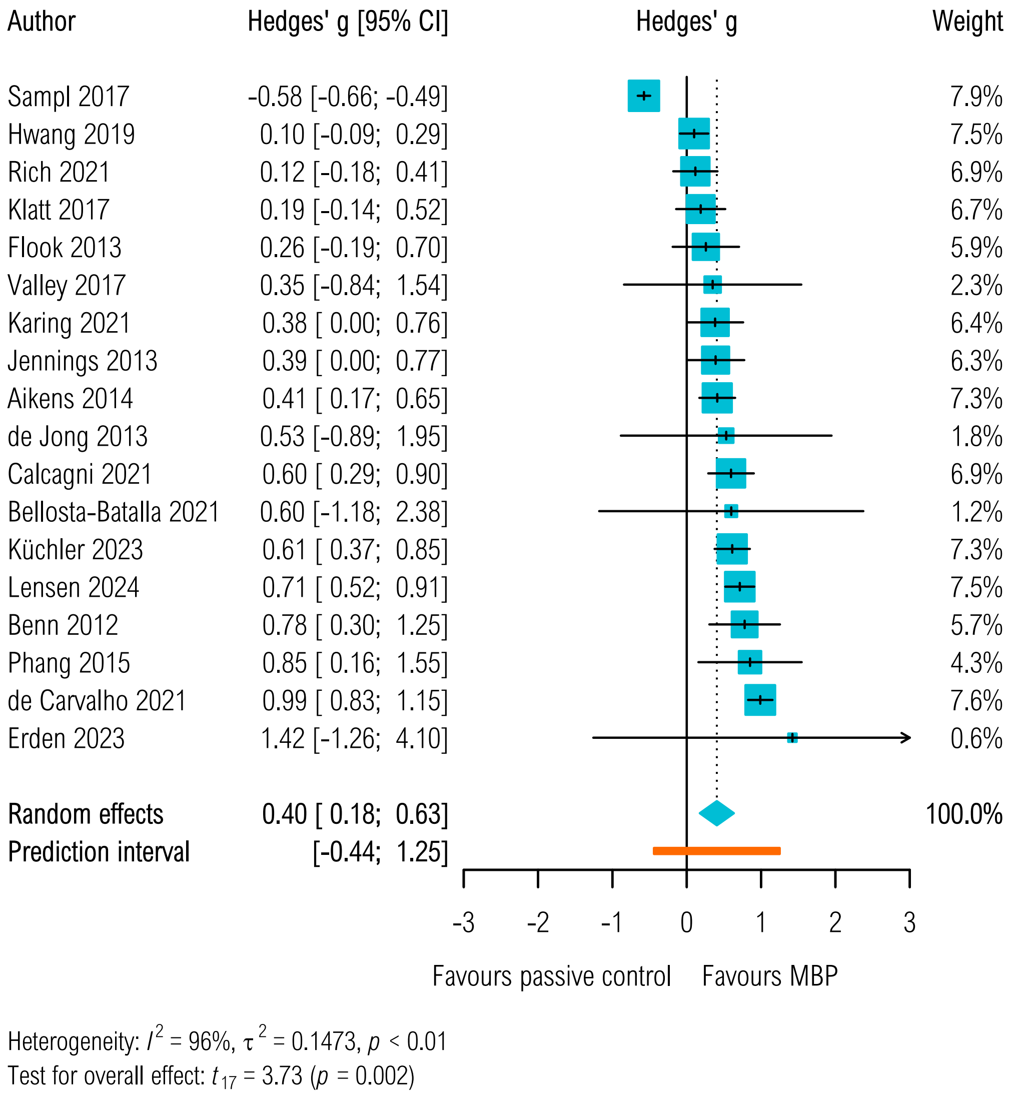

Figure S 5. Contextual performance up to 4 weeks post-intervention. Only passive control groups

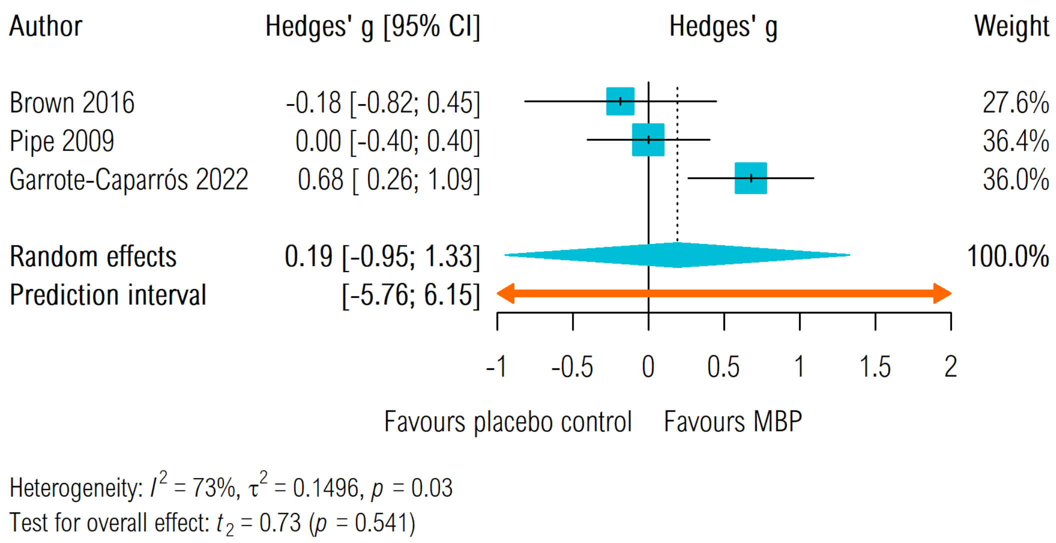

Figure S 6. Contextual performance up to 4 weeks post-intervention. Only placebo control groups

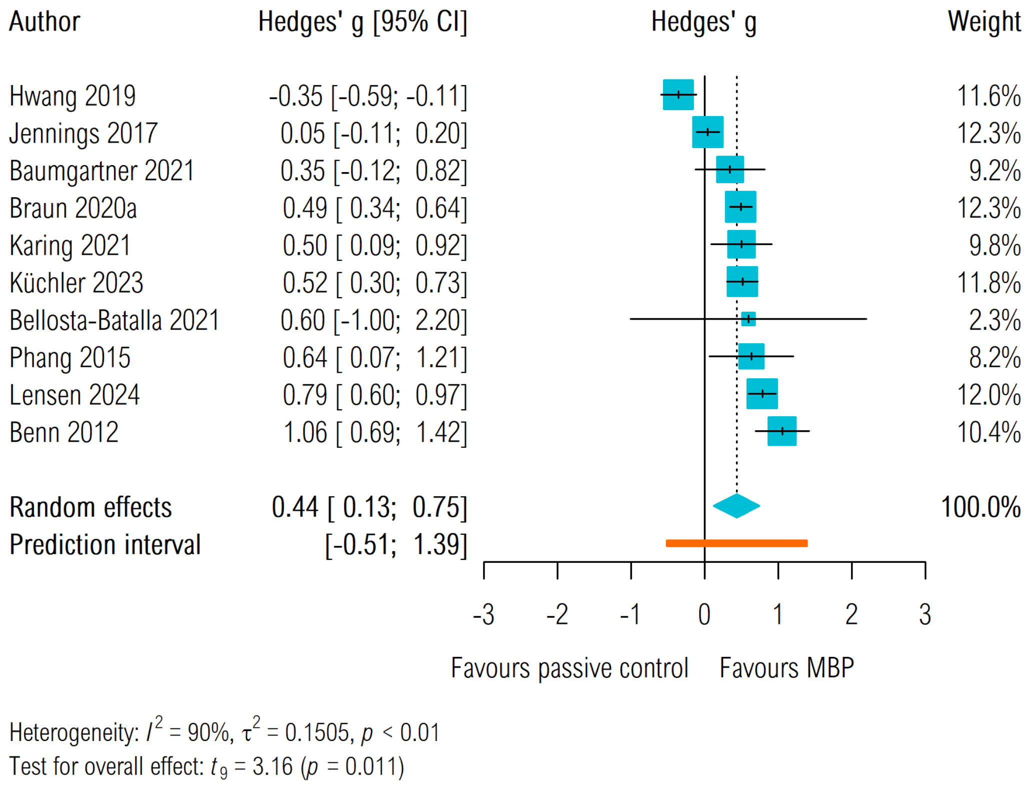

Figure S 7. Contextual performance 5-24 weeks post-intervention. Only passive control groups

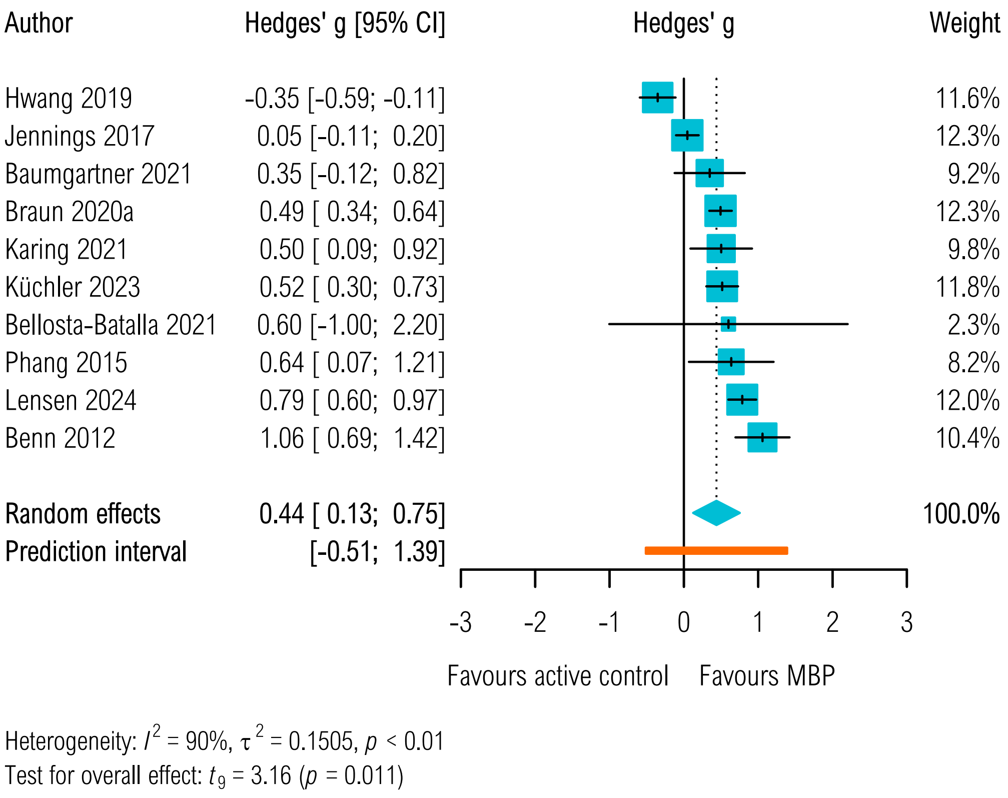

Figure S 8. Contextual performance 5-24 weeks post-intervention. Only active control groups

Adaptive performance

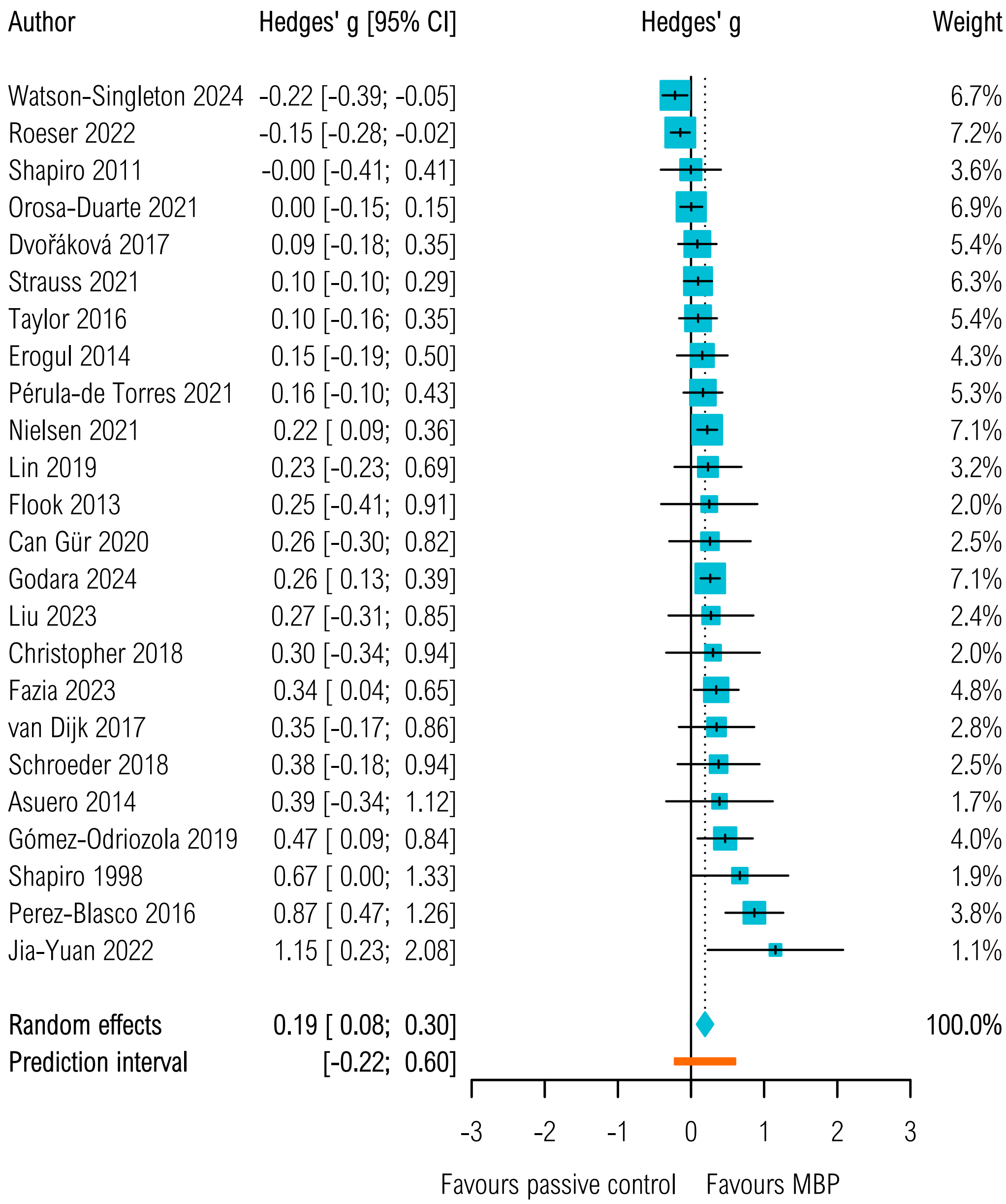

Heterogeneity: I^2 = 68%, τ^2 = 0.0366, p < 0.01
Test for overall effect: t_{23} = 3.54 (p = 0.002)

Figure S 9. Adaptive performance up to 4 weeks post-intervention. Only passive control groups

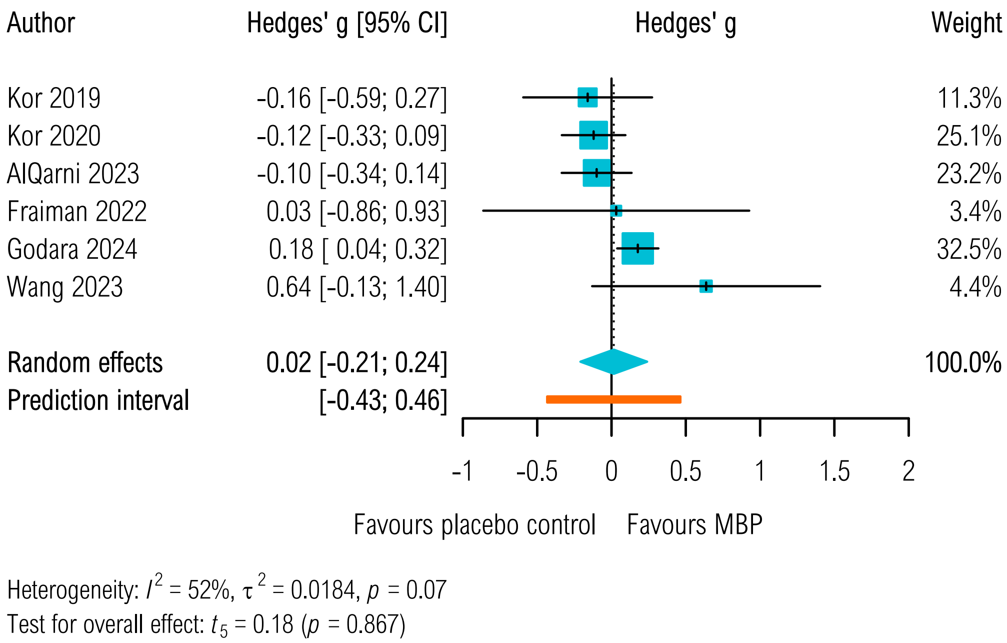

Figure S 11. Adaptive performance up to 4 weeks post-intervention. Only placebo control groups

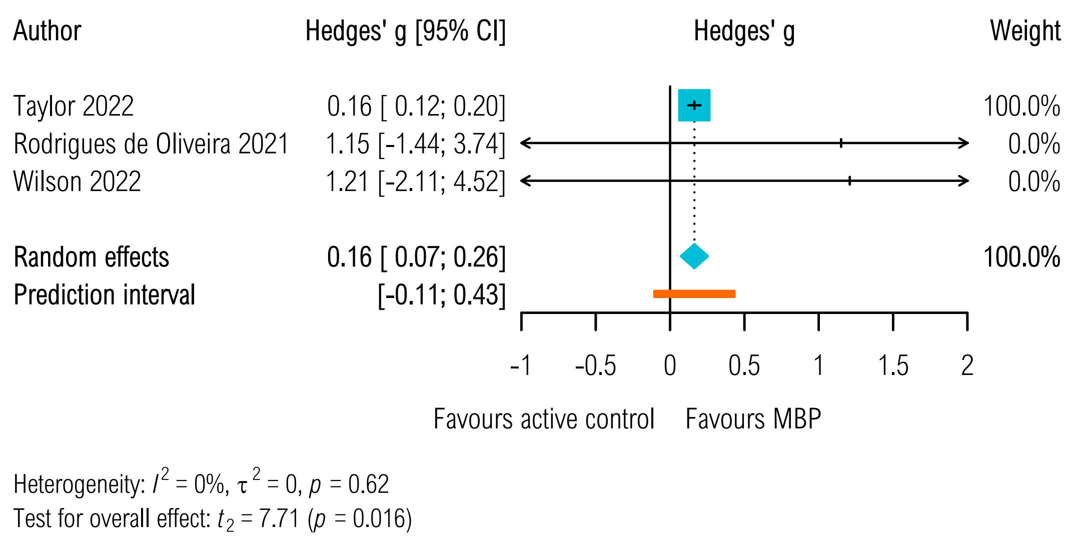

Figure S 12. Adaptive performance up to 4 weeks post-intervention. Only active control groups

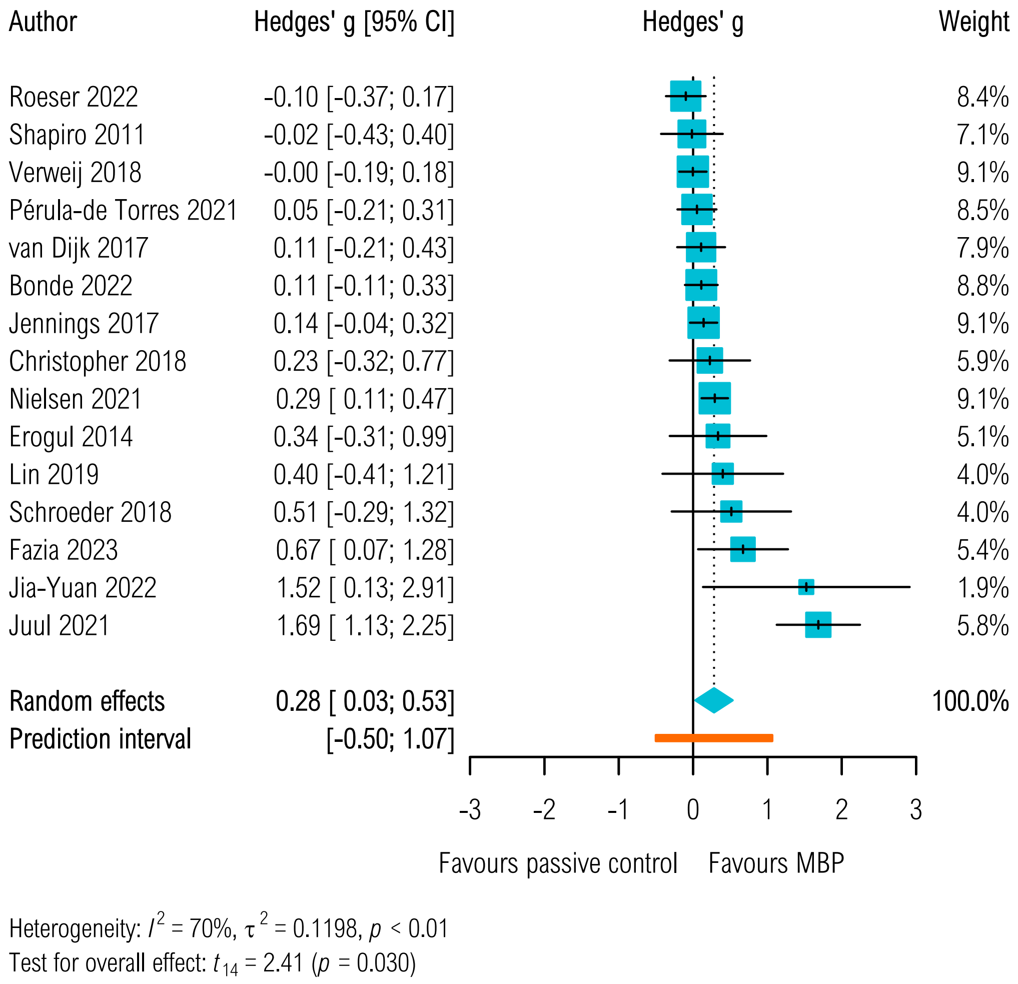

Figure S 13. Adaptive performance 5-24 weeks post-intervention. Only passive control groups

Counterproductive work behaviour

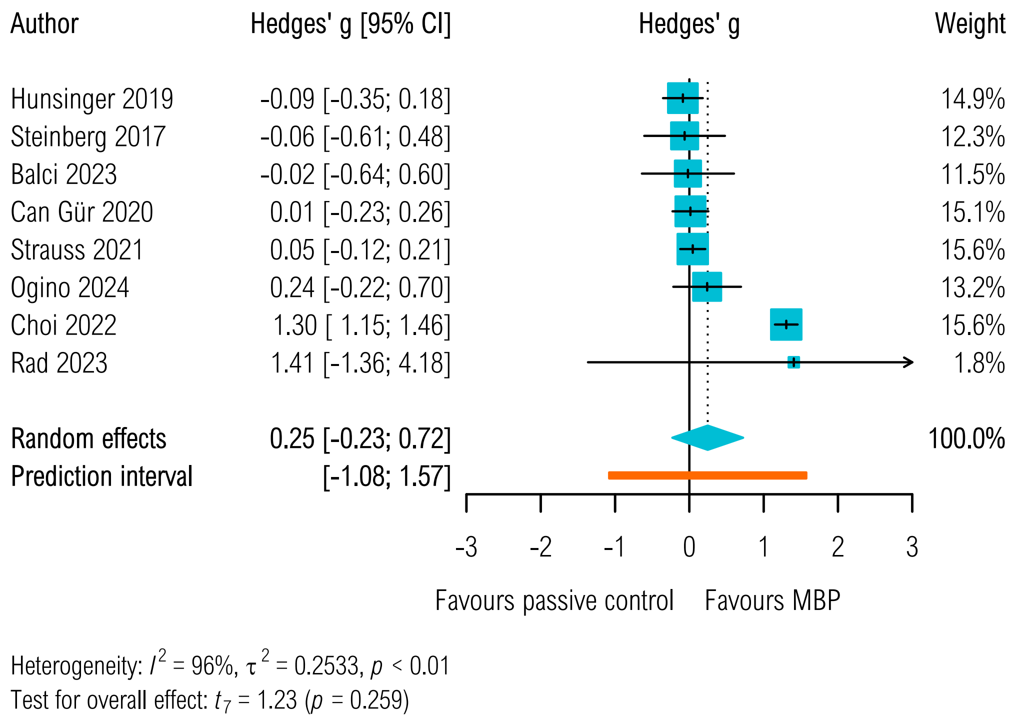

Figure S 14. Counterproductive work behaviour up to 4 weeks post-intervention. Only passive control groups

References

Allexandre, D., Bernstein, A. M., Walker, E., Hunter, J., Roizen, M. F., & Morledge, T. J. (2016). A Web-Based Mindfulness Stress Management Program in a Corporate Call Center: A Randomized Clinical Trial to Evaluate the Added Benefit of Onsite Group Support. *Journal of Occupational and Environmental Medicine*, *58*(3), 254–264.

John, L. K., Loewenstein, G., & Prelec, D. (2012). Measuring the prevalence of questionable research practices with incentives for truth telling. *Psychological Science*, *23*(5), 524–532. https://doi.org/10.1177/0956797611430953
